# Ripple-locked coactivity of stimulus-specific neurons and human associative memory

**DOI:** 10.1038/s41593-023-01550-x

**Published:** 2024-02-16

**Authors:** Lukas Kunz, Bernhard P. Staresina, Peter C. Reinacher, Armin Brandt, Tim A. Guth, Andreas Schulze-Bonhage, Joshua Jacobs

**Affiliations:** 1 Department of Epileptology, University Hospital Bonn, Bonn, Germany; 2 Epilepsy Center, Medical Center – University of Freiburg, Faculty of Medicine, University of Freiburg, Freiburg, Germany; 3 Department of Experimental Psychology, University of Oxford, Oxford, UK; 4 Oxford Centre for Human Brain Activity, Wellcome Centre for Integrative Neuroimaging, Department of Psychiatry, University of Oxford, Oxford, UK; 5 Department of Stereotactic and Functional Neurosurgery, Medical Center – University of Freiburg, Faculty of Medicine, University of Freiburg, Freiburg, Germany; 6 Fraunhofer Institute for Laser Technology, Aachen, Germany; 7 Department of Biomedical Engineering, Columbia University, New York, NY USA; 8 Department of Neurological Surgery, Columbia University Medical Center, New York, NY USA

**Keywords:** Hippocampus, Spatial memory, Neurophysiology, Psychology, Neural circuits

## Abstract

Associative memory enables the encoding and retrieval of relations between different stimuli. To better understand its neural basis, we investigated whether associative memory involves temporally correlated spiking of medial temporal lobe (MTL) neurons that exhibit stimulus-specific tuning. Using single-neuron recordings from patients with epilepsy performing an associative object–location memory task, we identified the object-specific and place-specific neurons that represented the separate elements of each memory. When patients encoded and retrieved particular memories, the relevant object-specific and place-specific neurons activated together during hippocampal ripples. This ripple-locked coactivity of stimulus-specific neurons emerged over time as the patients’ associative learning progressed. Between encoding and retrieval, the ripple-locked timing of coactivity shifted, suggesting flexibility in the interaction between MTL neurons and hippocampal ripples according to behavioral demands. Our results are consistent with a cellular account of associative memory, in which hippocampal ripples coordinate the activity of specialized cellular populations to facilitate links between stimuli.

## Abstract (web summary)

Using single-neuron recordings in patients with epilepsy, Kunz et al. show that stimulus-specific neurons activate together during hippocampal ripples when humans encode and retrieve associative memories.

## Main

**Fig. 1:**
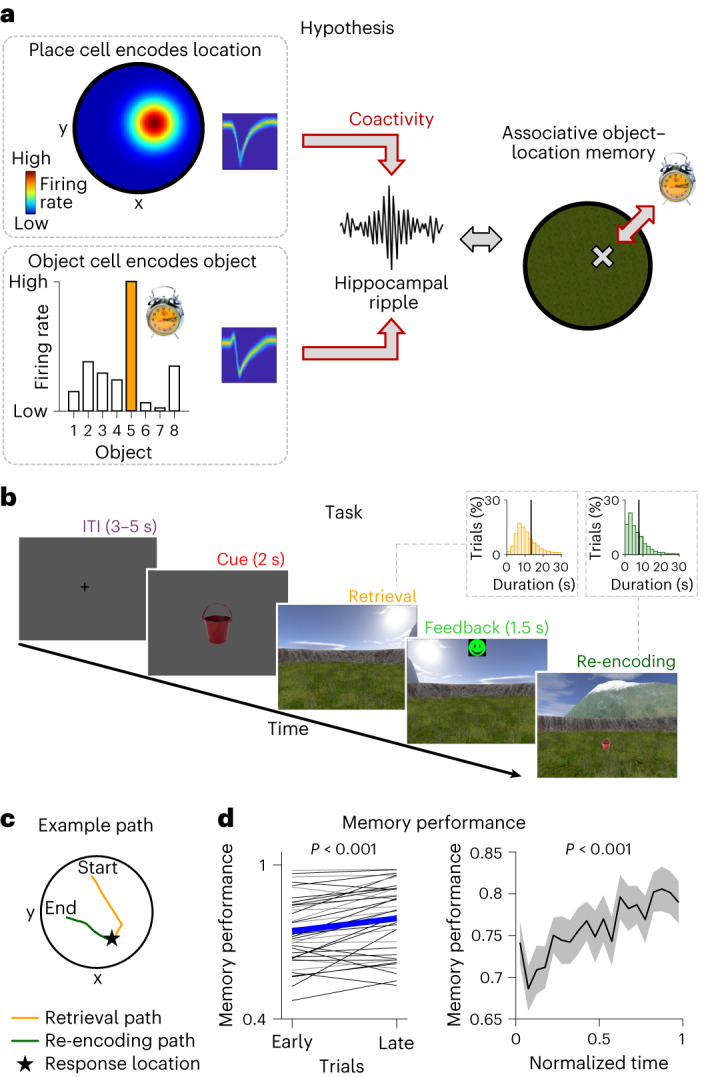
Hypothesis and associative object–location memory task. **a**, Illustration of the hypothesis that human associative object–location memory is linked to the coactivity of object cells and place cells during hippocampal ripples. We propose that the coactivity is specific to pairs of object and place cells that encode ‘associative information’, which are those cell pairs in which the location of the preferred object of the object cell is inside the place field of the place cell. **b**, Participants performed an associative object–location memory task while navigating freely in a virtual environment. After collecting eight different objects from their associated locations during an initial encoding period, participants performed a series of test trials. At the beginning of each test trial, after an inter-trial interval (‘ITI’), one of the eight objects was presented (‘Cue’), which the participant placed as accurately as possible at its associated location during retrieval (‘Retrieval’). Participants received feedback depending on the accuracy of their response (‘Feedback’) and collected the then-visible object from its correct location (‘Re-encoding’). Insets show histograms of the self-paced durations of retrieval (yellow) and re-encoding (green) periods. Black vertical lines indicate mean durations. **c**, Example paths during retrieval (yellow) and re-encoding (green) in one trial. Start, start location during retrieval. End, end location during re-encoding. The participant’s response location is indicated by a star. **d**, Left, memory performance during early versus late trials (median split) showing that participants improved their memories over time (two-sided paired *t*-test). Blue thick line indicates mean across sessions; thin lines indicate session-wise data (black, sessions with single-neuron recordings). Right, memory performance as a function of normalized time (two-sided Pearson correlation). Black, mean across sessions; gray shading, ±s.e.m. across sessions.

Associative memory is an essential cognitive function for everyday life that allows us to learn and remember relations between different stimuli^1^. Impairments in associative memory caused by aging and memory disorders^2,3^ are, thus, a growing problem for society, which makes it important to identify its underlying working principles in the brain. A large body of research has implicated the hippocampus and neighboring medial temporal lobe (MTL) regions in the encoding and retrieval of associative memories^4,5^, but its neural foundations remain far from understood. We thus aimed at elucidating possible mechanisms underlying associative memory in the human MTL at the single-cell level. Given prior theories on temporally precise neural binding in perception and memory^6–8^, we considered that the individual stimuli contributing to particular associative memories are encoded by separate sets of functionally specialized neurons and that these neurons interact transiently when individuals encode and retrieve the memories (Fig. 1a).

We examined this hypothesis on the neural basis of associative memory in the setting of object–location associations, which are particularly critical to behavioral functioning in everyday life by allowing us to know where important items are located in our spatial environments. We specifically investigated whether the encoding and retrieval of such object–location memories is correlated with the simultaneous activation of object cells, which represent specific objects^9,10^, and place cells, which code for particular spatial locations^11^. We predicted that these coactivations would occur in a temporally confined manner during hippocampal high-frequency oscillations, termed ‘ripples’^12–14^, which are considered important for synchronizing neural activity across brain regions^15–18^. Such ripple-locked coactivity of object and place cells could potentially underlie the encoding and retrieval of associative object–location memories by inducing and (re)activating synaptic connections between the object and place cells that represent the different memory elements^19^. In addition, ripple-locked coactivity of stimulus-specific neurons could elicit conjunctive memory representations in downstream neurons that respond only to the unique combination of all memory elements^20–22^.

Previous studies in both animals and humans discovered that hippocampal ripples are relevant to various cognitive functions^12–14^. Neural recordings in patients with epilepsy revealed that ripples correlate with memory encoding, retrieval and consolidation^23–28^. Rodent studies demonstrated that ripples are linked to precisely organized multicellular activity in the service of learning, memory and planning^12–14^. In particular, place cell sequences during hippocampal ripples were found to reflect contiguous navigation paths^29–32^, showing that ripple-locked single-neuron activity directly reflects behavior in a spatiotemporally meaningful way. It has remained unknown, however, whether hippocampal ripples also play a role in interconnecting functionally different types of neurons and whether they could thus help binding different mental contents into associative memories.

To investigate this idea, we conducted single-neuron and intracranial electroencephalographic (EEG) recordings from the MTL of human patients with epilepsy performing an associative object–location memory task in a virtual environment^9^. In line with our hypothesis that human hippocampal ripples support the formation and retrieval of associative memories by defining time windows for the coactivity of stimulus-specific neurons, we show that object-specific and place-specific neurons that represent the separate elements of particular object–location associations activate together at moments close to hippocampal ripples. These findings are consistent with the idea that ripple-locked coactivity of stimulus-specific neurons provides a neural mechanism for the formation and retrieval of associative memories and, more broadly, constitutes a key property of information processing in the human brain.

## Results

### Human hippocampal ripples and object–location memory

To study the neural mechanisms underlying human associative memory, we recorded single-neuron activity and intracranial EEG from the MTL of patients with epilepsy (Methods, Supplementary Table 1 and Supplementary Fig. 1). During the recordings, participants performed an associative object–location memory task in a virtual environment (Fig. 1b,c). In this task^9^, participants encoded the locations of eight different objects once during an initial encoding period and then performed a series of test trials that included periods for retrieving and re-encoding the object–location associations. Each test trial started with an inter-trial interval (ITI), followed by a cue period in which the participant viewed one of the eight objects that they had encountered during initial encoding. Then, in the retrieval phase, participants navigated to the remembered location of this object and received feedback depending on their response accuracy. After feedback, in the re-encoding phase, the object appeared in its correct location, and participants traveled to this location, allowing them to update their associative memory for this object–location pair. Thirty participants contributed a total of 41 sessions and performed 103 trials per session on average (for detailed information on all statistics in the main text, see Supplementary Table 2). They successfully formed associative memories between the objects and their corresponding locations, as their memory performance increased over the course of the task (paired *t*-test: *t*(40) = −4.788, *P* < 0.001; Fig. 1d and Supplementary Figs. 2 and 3).

**Fig. 2:**
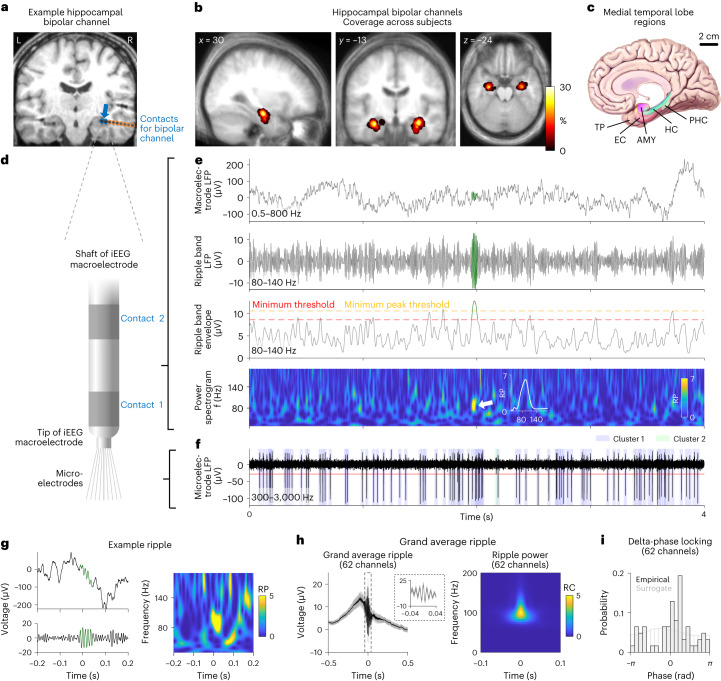
Ripples in the human hippocampus. **a**, Location of an example bipolar hippocampal channel (blue arrow). Blue circles, electrode contacts contributing to the bipolar channel; orange circles, other contacts. **b**, Probability distribution of all bipolar hippocampal channels, overlaid on the participants’ average MRI scan. **c**, MTL regions used for the recordings of LFPs and single-neuron activity. **d**, Illustration of the two innermost electrode contacts of an intracranial EEG macroelectrode with microelectrodes protruding from its tip. **e**, Analysis procedure for identifying ripples. Top to bottom: raw macroelectrode LFP; macroelectrode LFP filtered in the 80–140-Hz ripple band; smoothed envelope of the ripple band macroelectrode LFP; and spectrogram of the macroelectrode LFP. The power spectrum of each ripple event had to exhibit a global peak between 80 Hz and 140 Hz (white inset in bottom panel); otherwise, it was discarded as a false positive. **f**, Action potentials of two clusters from a microelectrode simultaneously recorded with the macroelectrode data. **g**, Raw voltage trace of an example hippocampal ripple (green) in the time domain (left) and its relative power spectrogram in the time–frequency domain (right). Time 0, ripple peak. **h**, Grand average voltage trace of hippocampal ripples across all channels in the time domain (left) and their power spectrogram in the time–frequency domain (right). Ripples were first averaged per channel and then across channels. Voltage traces are baseline corrected with respect to ±3 s around the ripple peak. Error bands, ±s.e.m. Ripple power is shown as the relative change with respect to the average power within ±3 s around the ripple peak. Time 0, ripple peak. **i**, Delta phase locking (0.5–2 Hz) of hippocampal ripples. Black histogram, ripple-associated delta phase for each channel. Gray histogram, delta phases of surrogate ripples. AMY, amygdala; EC, entorhinal cortex; HC, hippocampus; PHC, parahippocampal cortex; RC, relative change; RP, relative power; TP, temporal pole.

We identified human hippocampal ripples during the task by examining local field potentials (LFPs) from bipolar macroelectrode channels, which were located mostly in the anterior hippocampus (Fig. 2a–d and Supplementary Fig. 4). Following previous ripple detection algorithms^24,27,33^, we recorded a total of 35,948 ripples across all sessions (Fig. 2e–h). Preceding ripple detection, we conservatively excluded interictal epileptic discharges (IEDs; Supplementary Fig. 5) to help interpret our ripples and ripple-related findings as physiological^34^. We characterized the identified ripples with regard to various properties and confirmed that they reflected time periods with strongly elevated power at approximately 90 Hz (Fig. 2h and Supplementary Fig. 6), consistent with previous human studies^24,27,33^.

These periodic high-frequency events are presumably the human homolog of rodent sharp-wave ripples, although their overlap with human high gamma or broadband gamma activity^35,36^ and gamma or epsilon oscillations^37,38^, as well as their relation to ripples in animals, is not yet clear^34^. Because previous studies suggested that ripples are more strongly phase locked to low-frequency activity than gamma^15^, we examined the relation between ripples and low-frequency (delta, 0.5–2 Hz) activity of the LFPs in our data. We found that ripples were preferentially locked to a mean delta phase of 34° (Rayleigh test: *z* = 5.614, *P* = 0.003; Fig. 2i and Supplementary Fig. 7). Thus, consistent with previous results^15,23,39^, ripples in this dataset generally appeared at the descending phase of hippocampal slow oscillations, which may have a role in triggering the ripples^40^.

**Fig. 3:**
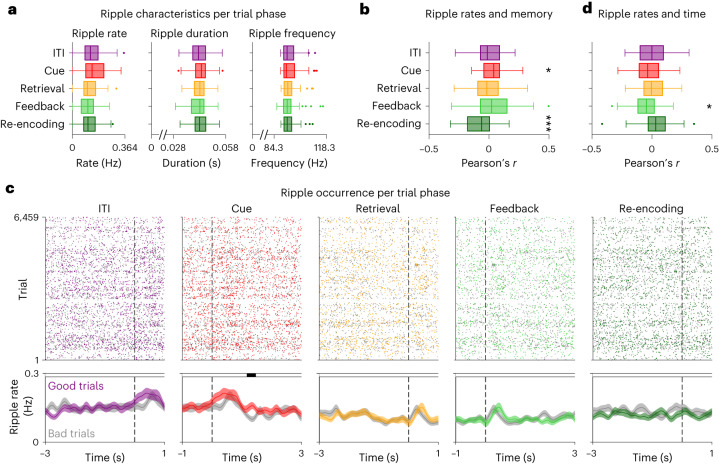
Ripples in the human hippocampus are linked to behavioral state and memory performance in an associative object–location memory task. **a**, Ripple characteristics during the different trial phases (*n* = 62 channels). As compared to ITI periods, ripple rates were increased during cue periods and reduced during retrieval, feedback and re-encoding periods (repeated-measures ANOVA: *P* < 0.001). Ripple durations showed a similar pattern as ripple rates (*P* = 0.046) but no significant post hoc comparisons. Ripple frequency was not modulated by trial phase (*P* = 0.690). **b**, Correlations between ripple rates and memory performance (*n* = 62 channels). For each channel, we computed across-trial correlations between ripple rates in a given trial phase and memory performance during the retrieval phase, and we tested the correlation values against 0 across channels (two-sided *t*-tests with Bonferroni correction for multiple comparisons). Increased ripple rates during cue periods were associated with better memory performance in the subsequent retrieval periods (*P*_corr._ = 0.038), and retrieval periods with poorer performance were followed by increased ripple rates during subsequent re-encoding periods (*P*_corr._ < 0.001). **c**, Time-resolved ripple occurrence across all trials as a function of absolute time relative to the onset (cue and feedback) or offset (ITI, retrieval and re-encoding) of a given trial phase (dashed vertical lines). Each dot is a ripple (colored dots, ripples during good-performance trials; gray dots, ripples during bad-performance trials). Colored lines and shadings, ripple rates during good-performance trials (‘good trials’; mean ± s.e.m. across channels); gray lines and shadings, ripple rates during bad-performance trials (‘bad trials’; mean ± s.e.m. across channels). Black shadings at top indicate time periods with significant differences between good and bad trials (two-sided cluster-based permutation tests: *P* < 0.025). **d**, Correlations between ripple rates and trial index (*n* = 62 channels). We tested the correlation values against 0 afterward (two-sided *t*-tests with Bonferroni correction). Ripple rates during feedback decreased over time (*P*_corr._ = 0.012). Box plots in **a**, **b** and **d** show center line, median; box limits, upper and lower quartiles; whiskers, minimum and maximum; and points, outliers. *P_corr._ < 0.05 and ***P_corr._ < 0.001.

We then asked how properties of hippocampal ripples related to the participants’ behavioral state and memory performance in our associative memory task (Fig. 3 and Methods). We observed that ripple rates varied as a function of trial phase (Fig. 3a); that increased ripple rates during cue periods were associated with better performance in the subsequent retrieval periods (Fig. 3b,c); and that retrieval periods in which participants showed poorer performance were followed by increased ripple rates during re-encoding (Fig. 3b). These associations between hippocampal ripples and behavior in our object–location memory task extend previously established links between hippocampal ripples and various memory processes in humans, which together suggest that hippocampal ripples are functionally important for encoding and retrieving memories^23,25–28,41^.

### Neural signature of hippocampal ripples across the human MTL

**Fig. 4:**
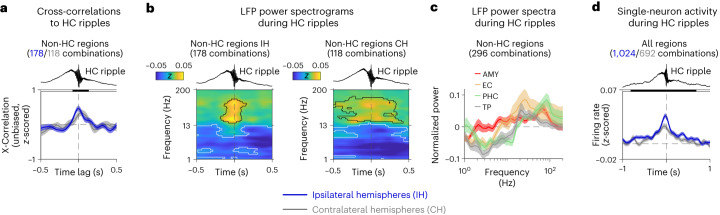
Hippocampal ripples are associated with changes in LFP power and firing rates across the human MTL. **a**, Cross-correlations between hippocampal ripples and ripples in extra-hippocampal MTL regions (temporal pole, amygdala, entorhinal cortex and parahippocampal cortex). Blue and gray numbers indicate the number of ipsilateral and contralateral channel pairs, respectively. Time 0, peak of hippocampal ripples. Cross-correlations are smoothed with a Gaussian kernel of 0.2-s duration and normalized by *z*-scoring cross-correlation values over time lags of ±0.5 s. Shaded region, mean ± s.e.m. across channel pairs. Black shading at top indicates cross-correlations from both ipsilateral and contralateral channel pairs significantly above 0 (one-sided cluster-based permutation test: *P* < 0.05). **b**, Time–frequency-resolved LFP power (*z*-scored relative to the entire experiment) in extra-hippocampal MTL regions during hippocampal ripples. Power values are smoothed over time with a Gaussian kernel of 0.2-s duration. Time 0, ripple peak. Black contours, significantly increased power; white contours, significantly decreased power (two-sided cluster-based permutation tests: *P* < 0.025). **c**, Normalized LFP power extracted from the time periods of hippocampal ripples and averaged over time. Error bands, ±s.e.m. **d**, Single-neuron firing rates (*z*-scored relative to the entire experiment) in hippocampal and extra-hippocampal regions during hippocampal ripples. Firing rates are smoothed over time with a Gaussian kernel of 0.2-s duration. Error bands, ±s.e.m. Blue and gray numbers indicate the number of ipsilateral and contralateral neuron–ripple channel pairs, respectively. Black shading at top indicates firing rates of ipsilateral and contralateral pairs significantly above 0 (one-sided cluster-based permutation test: *P* < 0.05). For region-specific and trial-phase-specific results, see Supplementary Figs. 8 and 9. AMY, amygdala; CH, contralateral hemispheres; EC, entorhinal cortex; HC, hippocampus; IH, ipsilateral hemispheres; PHC, parahippocampal cortex; TP, temporal pole; X-Correlation, cross-correlation.

Hippocampal ripples are neural events with brain-wide effects that are considered beneficial for establishing or strengthening synaptic connections (by means of Hebbian or other forms of synaptic plasticity)^13,15,42–44^. We thus reasoned that hippocampal ripples could support associative memory by triggering brain states in which otherwise separate neural representations become linked. To assess the general potential of awake hippocampal ripples to modulate neural activity across the human MTL, we performed analyses to quantify the effects of hippocampal ripples on single-neuron spiking and LFP changes in various regions of the human MTL (temporal pole, entorhinal cortex, amygdala, hippocampus and parahippocampal cortex; Fig. 4 and Supplementary Figs. 8 and 9).

We first tested whether ripple events that appeared in extra-hippocampal MTL regions were coupled to hippocampal ripples. We identified ripples in extra-hippocampal MTL regions using the same procedure as for hippocampal ripples and found that extra-hippocampal MTL ripples occurred in temporal proximity with hippocampal ripples using cross-correlation analyses (cluster-based permutation test: *P* < 0.001; Fig. 4a and Supplementary Figs. 8b and 9a). High-frequency oscillatory events, therefore, seem to be synchronized across the human MTL, in line with previous studies showing that hippocampal ripples are temporally coupled to ripples in various other brain areas^17,23,26,45^. This interregional ripple coupling may help bind separate groups of neurons together.

We also examined how hippocampal ripples related to brain state changes as reflected in the power of LFP oscillations. Across all macroelectrode channels in both ipsilateral and contralateral extra-hippocampal MTL regions, we found that the normalized LFP power at higher frequencies (>20 Hz) increased during hippocampal ripples (cluster-based permutation test across extra-hippocampal MTL channels ipsilateral to hippocampal ripple channels: *P* = 0.018; contralateral: *P* < 0.001). Inversely, normalized power at lower frequencies decreased during hippocampal ripples (ipsilateral: *P* < 0.001; contralateral: *P* < 0.001; Fig. 4b,c and Supplementary Figs. 8c and 9b). These MTL-wide power changes were strongest at the moments when hippocampal ripples occurred but started before and ended after them (see also ref. ^46^). Given that increased high-frequency power and decreased low-frequency power are indicators of elevated neuronal excitation^47^, these results suggest that hippocampal ripples are associated with an excitatory state of the human MTL and further indicate that hippocampal ripples may support brain states suitable for inducing and activating synaptic connections between otherwise segregated neurons.

In a third analysis of the large-scale effects of hippocampal ripples, we examined single-neuron spiking across the MTL at the moments of hippocampal ripples. Across all 27 sessions with single-neuron recordings, we recorded a total of 1,063 neurons across multiple regions (Fig. 2c,f and Supplementary Fig. 1), including temporal pole, entorhinal cortex, amygdala, hippocampus and parahippocampal cortex. Overall, neuronal firing rates increased when hippocampal ripples occurred (cluster-based permutation test: *P* < 0.001), whereby neuronal firing rates started to rise about 0.25 s before the ripples peaked (Fig. 4d). This increased spiking during hippocampal ripples was strongest for neurons in the hippocampus itself and the amygdala but was also present in other regions (Supplementary Figs. 8d and 9c). Behavior-related analyses showed that ripple-locked firing rate increases were similar across the different trial phases and for varying levels of memory performance (Supplementary Figs. 9 and 10).

Together, these results show that hippocampal ripples are associated with broad changes in neural activity across the human MTL. When hippocampal ripples occurred, there was an increased probability of ripple-like events in extra-hippocampal MTL regions; a shift in LFP power from lower to higher frequencies in these regions; and an increase in MTL-wide neuronal spiking. Related long-range effects of hippocampal ripples have been described in both animals and humans^15,16,18,26,46^. Through their excitatory effects, hippocampal ripples may play a key role in the formation and retrieval of associative memories by combining diverse patterns of neural activity from multiple regions, although studies with causal manipulations are needed to further evaluate this hypothesis.

### Neurons in the human MTL are tuned to objects and locations

To examine whether ripples in the human hippocampus are linked to synchronized activity of object-specific and place-specific cells during associative memory processes, we next tested for these cell types in our data. We identified neurons as object cells if they increased their firing rates in response to a particular object^9,10^, and we considered neurons as place cells if they activated when the participant was at a particular spatial location in the virtual environment^11^.

**Fig. 5:**
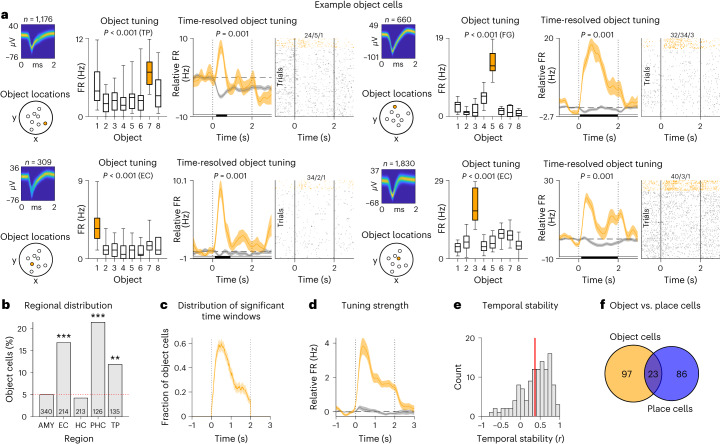
Neurons in the human MTL encode specific objects. **a**, Example object cells. For each cell, from left to right: action potentials as density plot; locations of the objects in the environment; absolute firing rates in response to the different objects during the cue period (statistics are from one-sided permutation tests, using a total of *n* = 1,176, 660, 309 and 1,830 action potentials during all cue periods, respectively); time-resolved firing rates (baseline corrected relative to −1 s to 0 s before cue onset) for the preferred and the unpreferred objects (error bands, ±s.e.m.); and spike raster for all trials. Time 0, cue onset. Orange, data for the preferred object; gray, data for unpreferred objects. Box plots show center line, median; box limits, upper and lower quartiles; and whiskers, minimum and maximum. Black shading below the time-resolved firing rates, significant difference between firing rates (one-sided cluster-based permutation test: *P* < 0.05). **b**, Distribution of object cells across brain regions; red line, 5% chance level. Object cells were significantly prevalent in the entorhinal cortex, parahippocampal cortex and temporal pole (two-sided binomial tests versus chance with Bonferroni correction for multiple comparisons: *P*_corr._ < 0.001, *P*_corr._ < 0.001 and *P*_corr._ = 0.006, respectively). **c**, Distribution of significant time windows across object cells (mean ± s.e.m.). **d**, Tuning strength across object cells (mean ± s.e.m.). Orange, data for the preferred object; gray, data for unpreferred objects. **e**, Temporal stability of object cell tuning. Red line, mean. **f**, Overlap between object and place cells. AMY, amygdala; EC, entorhinal cortex; FG, fusiform gyrus; HC, hippocampus; PHC, parahippocampal cortex; TP, temporal pole. **P_corr._ < 0.01 and ***P_corr._ < 0.001.

Each object cell had a particular ‘preferred object’ in response to which it activated most strongly during the cue period. For example, the first object cell shown in Fig. 5a exhibited its highest firing when the participant viewed object 7. We observed 120 object cells (11% of all neurons; binomial test: *P* < 0.001; Supplementary Table 1), which were most prevalent in the entorhinal cortex, parahippocampal cortex and temporal pole (Fig. 5b). Object cells activated most strongly in response to their preferred object during the first second after cue onset (Fig. 5c); their firing rates returned to baseline shortly after the object disappeared from screen (Fig. 5d); and their tuning curves were overall stable over time (one-sample *t*-test: *t*(118) = 10.387, *P* < 0.001; Fig. 5e). Most object cells were ‘pure’ object cells in the sense that they did not also meet the criteria for being place cells (81% of all object cells; Fig. 5f).

**Fig. 6:**
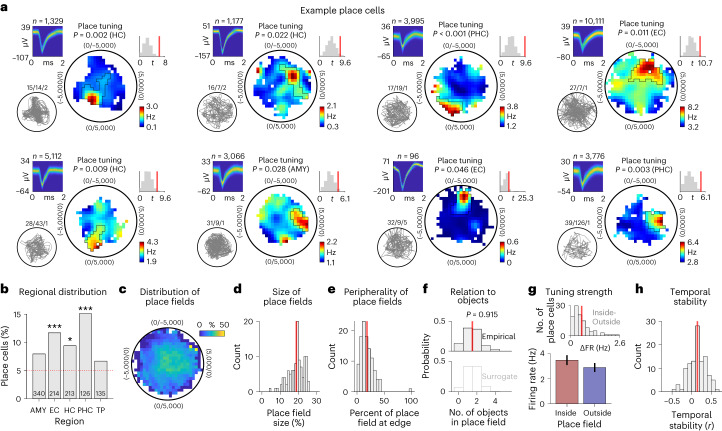
Neurons in the human MTL encode specific spatial locations. **a**, Example place cells. For each cell, from left to right: action potentials as density plot; navigation path of the participant through the environment (gray line); smoothed firing rate map (unvisited areas are shown in white); empirical *t*-statistic (red line) and surrogate *t*-statistics (gray histogram) from two-sided unpaired *t*-tests (using a total of *n* = 1,329, 1,177, 3,995, 10,111, 5,112, 3,066, 96 and 3,776 action potentials, respectively); and color bar, firing rate. **b**, Distribution of place cells across brain regions; red line, 5% chance level. Place cells were significantly prevalent in the entorhinal cortex, hippocampus and parahippocampal cortex (two-sided binomial tests versus chance with Bonferroni correction for multiple comparisons: *P*_corr._ < 0.001, *P*_corr._ = 0.034 and *P*_corr._ < 0.001, respectively). **c**, Spatial distribution of the place fields of all place cells (in percent relative to the spatial distribution of the firing rate maps). **d**, Histogram of place field sizes. Place field sizes are expressed in percent relative to the sizes of the firing rate maps by dividing the number of spatial bins being part of the place field by the number of spatial bins being part of the firing rate map. Unoccupied spatial bins are ignored. Red line, mean. **e**, Histogram of place field fractions that were next to the edges of the firing rate maps, quantifying the peripherality of the place fields. Red line, mean. **f**, Histogram of the number of objects inside place fields. The number of objects inside empirical place fields (top) is not increased as compared to surrogate place fields (bottom; two-sided two-sample Kolmogorov–Smirnov test). Red line, mean. **g**, Firing rate of place cells inside versus outside the place fields (*n* = 109 place cells). Bars and error bars show mean ± s.e.m. Histogram at top shows the distribution of cell-wise differences in firing rates inside minus outside the place fields (ΔFR). **h**, Temporal stability of the firing rate maps of place cells; red line, mean. AMY, amygdala; EC, entorhinal cortex; HC, hippocampus; PHC, parahippocampal cortex; TP, temporal pole. *P_corr._ < 0.05 and ***P_corr._ < 0.001.

Each place cell activated preferentially when the participant was at a particular location in the virtual environment (Fig. 6a). Over the past decades, studies in rodents have provided ample evidence for such place tuning in neurons of the hippocampus and surrounding brain areas^11,48^. Across all neurons, we identified 109 place cells (10%; binomial test: *P* < 0.001) and found them at significant levels in several regions, including the entorhinal cortex, hippocampus and parahippocampal cortex (Fig. 6b), consistent with previous work^49^. The firing fields of place cells were broadly distributed across the virtual environment (Fig. 6c–f), showing that all parts of the environment were neurally represented. The cells’ firing rates were about 20% higher inside versus outside the place fields (Fig. 6g), and their firing patterns were stable over time (one-sample *t*-test: *t*(108) = 7.080, *P* < 0.001; Fig. 6h), indicating that spatial information was robustly encoded by the place cells of our dataset. Place and object tuning was largely independent of each other because place and object cells only marginally overlapped with conjunctive cells, which exhibited significant place tuning solely during trials with one particular object (Supplementary Fig. 11). Object and place cells showed some variability with regard to different electrophysiological properties and, accordingly, were present among both putative pyramidal cells and putative interneurons (Supplementary Fig. 12).

Together, these results show that cells in the human MTL encoded the separate elements of to-be-established and to-be-remembered object–location memories in our task: object cells encoded individual objects, and place cells encoded particular spatial locations. This allowed us to next probe the coactivity of object and place cells during hippocampal ripples as a potential neural correlate of associative memory.

### Ripple-locked cellular coactivity and object–location memory

Having examined hippocampal ripples and stimulus-specific (object and place) neurons separately, we next tested our principal hypothesis that object and place cells activate together during the same hippocampal ripples when individuals form and retrieve associative memories that link the separate elements that these neurons encode. We reasoned that coactivity during memory formation could facilitate synaptic connections between the stimulus-specific neurons^43,50^ (through Hebbian or other forms of synaptic plasticity or as a byproduct of a more fundamental process, such as neurotransmitter-dependent upregulation of neuronal excitability^13,15,42–44^) and that coactivity during memory retrieval could reflect their reciprocal activation through their previously established synaptic connections.

**Fig. 7:**
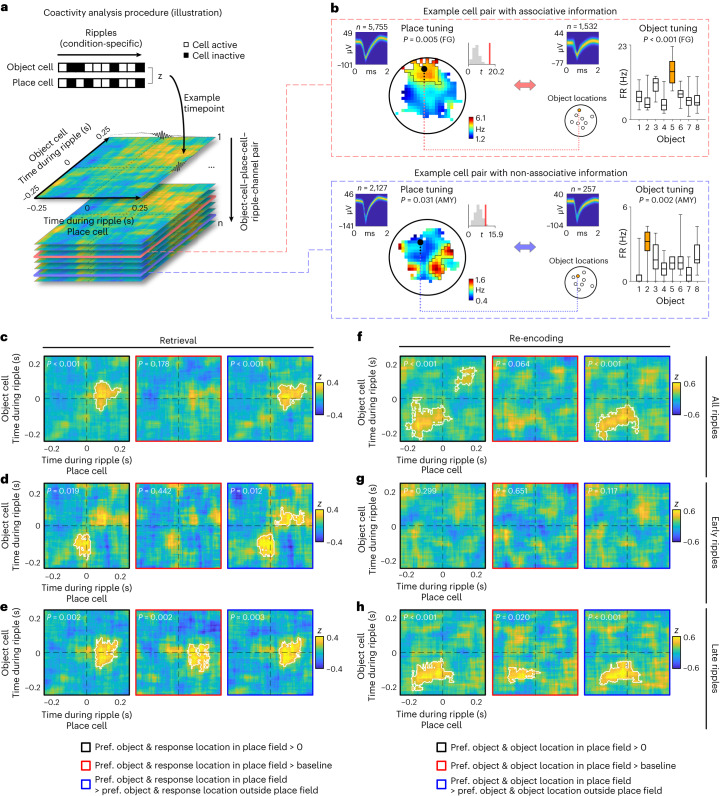
Ripple-locked coactivity of object and place cells during the retrieval and formation of associative memories. **a**, Analysis of ripple-locked coactivity of object and place cells (illustration). **b**, Example pairs of object and place cells with associative and non-associative information. For both place cells, from left to right: action potentials as density plot; smoothed firing rate map (unvisited areas are shown in white); empirical *t*-statistic (red line) and surrogate *t*-statistics (gray histogram) from two-sided unpaired *t*-tests (using a total of *n* = 5,755 and 2,127 action potentials, respectively); and color bar, firing rate. For both object cells, from left to right: action potentials as density plot; locations of the objects in the environment; and absolute firing rates in response to the different objects during the cue period (statistics are from one-sided permutation tests, using a total of *n* = 1,532 and 257 action potentials during all cue periods, respectively). Orange, data for the preferred object; gray, data for unpreferred objects. Box plots show: center line, median; box limits, upper and lower quartiles; and whiskers, minimum and maximum. **c**, Coactivity maps estimated using all ripples during retrieval periods, considering only trials in which the participant is asked to remember the location of the preferred object of the object cell and in which the participant’s response location is inside the place field of the place cell. Left, comparison of the coactivity maps against chance (that is, 0). Middle, comparison against baseline coactivity maps. Right, comparison against coactivity maps estimated using ripples from trials in which the participant is asked to remember the location of the preferred object of the object cell and in which the participant’s response location is outside the place field of the place cell. **d**, Same as in **c** for early retrieval-related hippocampal ripples. **e**, Same as in **c** for late retrieval-related hippocampal ripples. **f**, Coactivity maps estimated using all ripples from the re-encoding periods, considering only trials in which the participant is asked to re-encode the correct location of the preferred object of the object cell and in which the object’s correct location is inside the place cell’s place field. Left, comparison of the coactivity maps against chance (that is, 0). Middle, comparison against baseline coactivity maps. Right, comparison against coactivity maps estimated using ripples from trials in which the participant is asked to re-encode the location of the preferred object of the object cell and in which the object’s correct location is outside the place cell’s place field. **g**, Same as in **f** for early re-encoding-related hippocampal ripples. **h**, Same as in **f** for late re-encoding-related hippocampal ripples. White lines in **c**–**h** delineate significant clusters based on one-sided cluster-based permutation tests, which control for multiple comparisons and whose *P* values are stated at the upper left (see the main text and Supplementary Table 2 for Bonferroni-corrected *P* values). AMY, amygdala; FG, fusiform gyrus; pref., preferred.

We thus analyzed whether simultaneously recorded object and place cells were active during the same ripples and performed this analysis separately for ripples during retrieval and during re-encoding. For each combination of an object cell, a place cell and a ripple channel, we computed coactivity scores between the two cells across ripples that indicated how often both cells were (in)active during the same ripples (where the coactivity scores controlled for the overall activity level of both cells). We systematically and independently varied the ripple-locked time bin for determining the underlying activity of both cells and thus obtained a two-dimensional time-by-time coactivity map for each cell pair. These coactivity maps showed the coactivity scores in various time bins between −0.25 s and +0.25 s around the ripple peaks (101 time bins; 100-ms duration per bin; 95% overlap between neighboring bins), where high coactivity scores indicated consistent across-ripple coactivations of both cells in specific time windows relative to the ripple peaks (Fig. 7a and Supplementary Fig. 13).

Based on our principal hypothesis (Fig. 1a), our analysis focused on the ripple-locked coactivity of object and place cell pairs in which the preferred object of the object cell was located inside the place field of the place cell. Cells in these ‘associative cell pairs’ represented the different pieces of information that the participants were asked to associate with each other (Fig. 7b). For retrieval periods, the location of the preferred object of the object cell was defined by the participant’s response locations, whereas, during re-encoding periods, it was defined by the object’s true location. We hypothesized that coactivations of associative object and place cells during retrieval would indicate that the participant remembered a particular location given a specific object, whereas their coactivations during re-encoding would indicate that the participant aimed at (re)learning or updating the correct location of a given object. Overall, we thus reasoned that the ripple-locked coactivity of associative cell pairs would be linked to the encoding and retrieval of particular associative memories.

To evaluate the statistical significance of the coactivity for pairs of object and place cells encoding associative information on each trial, we designed a series of three complementary statistical tests (Supplementary Fig. 14). These tests contrasted the coactivity maps (1) against chance to see whether the cell pairs showed a positive increase in coactivity; (2) against coactivity maps from a baseline period to show that the coactivity increases were specific to the timing of ripples; and (3) against the coactivity maps of non-associative object and place cell pairs (where the preferred object of the object cell was located outside the place cell’s place field) to demonstrate that the coactivity increases were unique to cell pairs representing the exact components of associative memories. Together, these three tests provided robust information about whether associative pairs of object and place cells exhibited coactivity during hippocampal ripples.

We first examined ripple-locked coactivity of associative object cell–place cell pairs during retrieval (Fig. 7c–e and Supplementary Figs. 15a–c and 16a). Across all retrieval periods, we found indications of ripple-locked coactivity in associative cell pairs, as their coactivity scores were significantly positive and significantly greater than in control cell pairs that did not encode associative information (cluster-based permutation test versus chance: *P* < 0.001; versus non-associative cell pairs: *P* < 0.001; Fig. 7c). The comparison against the coactivity maps from the baseline period was not significant (*P* = 0.178), however, which indicates that ripple-locked coactivity of object and place cells during retrieval was not fully pronounced when estimated across the entire task. We therefore performed this analysis separately for ‘early’ hippocampal ripples (the first *n* / 2 individual ripples per session, where *n* is the total number of ripples per session) and ‘late’ ripples (the last *n* / 2 ripples per session), because we hypothesized that retrieval-related coactivity would be expressed more strongly during late ripples, after the participants had already formed associations between the objects and their corresponding locations (Fig. 1d). Indeed, when considering only late ripples, we found clear ripple-locked coactivity of object cell–place cell pairs representing associative information, as the coactivity maps of these cell pairs were significant for all three statistical tests (versus chance: *P* = 0.004; versus baseline: *P* = 0.004; versus non-associative cell pairs: *P* = 0.006; *P* values are Bonferroni corrected for analyzing both early and late ripples; Fig. 7e). Similarly strong effects were not present when considering early ripples (versus chance: *P* = 0.038; versus baseline: *P* = 0.884; versus non-associative cell pairs: *P* = 0.025; Bonferroni corrected; Fig. 7d), which explains why ripple-locked coactivity during retrieval was not fully pronounced when considering all ripples.

These findings demonstrate that ripples from later retrieval periods were associated with coactivations in object and place cells that represented associative information of to-be-retrieved associative memories. We assume that the synaptic connections between object and place cells gradually developed over time, which is why the ripple-locked coactivity of associative object and place cells was not robustly visible during early retrieval periods. Follow-up analyses confirmed these results by showing that ripple-locked coactivity was present at the level of individual cell pairs (Supplementary Fig. 17) and that the same effects were also evident with another method for estimating coactivity maps (Supplementary Fig. 18) or when considering only the data from first experimental sessions (Supplementary Fig. 19). Adding the cue periods to the analysis resulted in qualitatively identical ripple-locked coactivations as for retrieval phases alone (Supplementary Fig. 20a), indicating that similar ripple-related neural processes occurred during cue and retrieval (although we did not have enough data to examine ripple-locked coactivity during cue periods alone).

When inspecting the coactivity maps, we observed that the increased coactivations occurred at the moment of the ripple peaks for object cells and slightly after the ripple peaks for place cells (Fig. 7e and Supplementary Fig. 21a). This observation may have two implications: it may suggest that the neuronal connections are directed, such that object cells activate slightly earlier than place cells, and it may also indicate that hippocampal ripples can help trigger coactivations of MTL neurons, in line with the broader idea that information propagates from the hippocampus to extra-hippocampal regions during retrieval^51^.

Next, to understand the correlation between ripple-coordinated single-neuron activity and the formation of associative memories, we examined ripple-locked coactivity of the object cell–place cell pairs that represented associative information during the task’s re-encoding periods (Fig. 7f–h and Supplementary Figs. 15d–f and 16b). Similar to our retrieval-related results, we found indications of increased coactivity in associative object cell–place cell pairs when considering ripples from all re-encoding periods, whereby the comparisons against chance and non-associative cell pairs were significant, but the comparison against the baseline period was not significant (versus chance: *P* < 0.001; versus baseline: *P* = 0.064; versus non-associative cell pairs: *P* < 0.001; Fig. 7f). Parallel to our coactivity analyses during retrieval, we therefore examined re-encoding-related coactivations separately for early and late ripples. Indeed, we found again that, when only late ripples were considered, associative pairs of object and place cells showed robust increases in coactivity that were significant for all three statistical tests (versus chance: *P* < 0.001; versus baseline: *P* = 0.040; versus non-associative cell pairs: *P* < 0.001; *P* values are Bonferroni corrected for performing this analysis on both early and late ripples; Fig. 7h and Supplementary Figs. 17–19). In contrast, such coactivations were not present for early re-encoding-related ripples (versus chance: *P* = 0.599; versus baseline: *P* = 1; versus non-associative cell pairs: *P* = 0.235; Bonferroni corrected; Fig. 7g), which again explains why ripple-locked coactivity during re-encoding was not fully pronounced when measured across the entire session.

These results demonstrate that ripple-locked coactivity between associative object and place cells occurred when participants re-encoded object–location associations during later periods of the task. During these later task periods, the participants were already familiar with the associations. We therefore propose that ripple-locked coactivity during re-encoding is more closely related to the stabilization, updating or early consolidation of associative object–location memories rather than to their initial formation^6,12^. Extending this analysis to both the re-encoding and their subsequent ITI phases, we found similar coactivations as for re-encoding phases alone (Supplementary Fig. 20b), suggesting that the neural activity during ITI phases extended the neural processes from re-encoding. In comparison to the timing of ripple-locked coactivity during retrieval, the increased coactivations during late re-encoding-related ripples shifted to slightly precede the hippocampal ripples (Fig. 7h and Supplementary Fig. 21b), which may relate to the propagation of information from extra-hippocampal regions to the hippocampus during memory encoding.

To further investigate whether the distinction between early and late ripples paralleled a distinction between ripples occurring before versus after the initial formation of the object–location memories, we estimated the time of strongest improvement in memory performance for each object. We reasoned that this time reflected the moment when the object–location associations emerged initially. We then grouped the ripples according to whether they occurred before or after this time of strongest memory improvement. Ripple-locked coactivations of object and place cells before and after initial memory formation appeared very similar to their coactivity patterns during early and late ripples, respectively (Supplementary Fig. 22). This suggests that the coactivations during late ripples from both retrieval and re-encoding periods were (at least partly) dependent upon an initial formation of the object–location associations.

Studies in rodents highlighted the functional relevance of ripples during immobility^12,14^. We thus differentiated between ripples during movement versus non-movement and examined whether the coactivity effects appeared preferentially during non-movement-related ripples. As hypothesized, the increased ripple-locked coactivity between associative object and place cells was driven by ripples during non-movement periods (Supplementary Fig. 23). This indicates that the relevance of ripples for human memory changes between different movement states of the individual. Supplemental control analyses showed that the neurons’ brain regions and average firing rates were not systematically related to their coactivity scores (Supplementary Fig. 24).

Overall, these results provide empirical support for our principal hypothesis that human hippocampal ripples are linked to coactivations of stimulus-specific neurons (which represent particular objects and locations in this study) during the formation and retrieval of associative memories.

## Discussion

Associative memory allows us to learn and later retrieve links between previously unrelated stimuli^1^. In this study, we conducted neural recordings in patients with epilepsy performing an object–location memory task in a virtual environment to investigate possible roles of stimulus-specific single neurons and hippocampal ripples for human associative memory. Object-specific and place-specific neurons exhibited correlated activity fluctuations during hippocampal ripples when participants formed and retrieved associative object–location memories. This phenomenon of ripple-locked coactivity of stimulus-specific neurons is consistent with a cellular account of how humans interconnect previously unrelated stimuli and how they recall a stimulus from memory after being cued with another stimulus. Hippocampal ripples may support these functions by binding distinct cellular populations together.

### Neural mechanisms of associative memory

Different theories suggest explanations of how neural circuits encode associative memories^1,8^. Following the ‘conjunctive hypothesis’, the relevant neural circuits may contain conjunctive representations as a substrate for associative memories, in which neurons encode the unique combination of two or more stimuli^20^. Such conjunctive neurons do not respond to the different stimuli in isolation but only when the stimuli are encoded or retrieved together^20–22^. In contrast, following the ‘coactivity hypothesis’, associative memories are enabled by stimulus-specific neurons that encode the individual elements of associative memories and that coactivate temporarily when individuals encode or recall these memories^6–8^. Coactivity during memory encoding would establish synaptic connections between the stimulus-specific neurons^42,50^, and coactivity during retrieval would reflect the mutual activation of stimulus-specific neurons based on their previously established synaptic connections.

In line with the ‘conjunctive hypothesis’, several studies described the existence of conjunctive cells in the MTL. In rodents, the spiking of hippocampal neurons encodes particular object–location associations, and this conjunctive code strengthens with learning^22^. Neurons in the monkey hippocampus change their firing when monkeys learn particular scene–eye movement associations^21^, and, in humans, hippocampal neurons seem to respond to unique combinations of several stimuli^52^. We obtained further evidence for conjunctive coding by observing neurons that represented locations only during trials with a particular object. These findings support the view that associative memory is linked to conjunctive neural coding.

Here we provide empirical support for the ‘coactivity hypothesis’ of associative memory by identifying single-neuron representations of the separate memory elements and by demonstrating how these separate neural representations activate together at particular timepoints. Specifically, our results show that stimulus-specific object and place cells coactivate during hippocampal ripples when humans encode or retrieve object–location associations. We found that this ripple-locked coactivity was specific to object and place cells jointly representing associative information, that it was significantly expressed only during later parts of the task and that it occurred mostly after initial memory formation had taken place. These results support the ‘coactivity hypothesis’ by indicating that cellular coactivations play a role in human associative memory. Given that not only associative memories require an interplay between different mental contents, we propose that ripple-locked coactivations could be relevant to various cognitive functions that involve transient interactions between otherwise independent neural representations. For example, episodic memories comprise event, time and place information and may, thus, rely on transient interactions between concept cells containing semantic information^10^, time cells coding temporal information^53^ and spatially modulated cells representing locations and directions^9,11^.

The ‘conjunctive hypothesis’ and the ‘coactivity hypothesis’ are not mutually exclusive, and various other mechanisms, such as fan cells in the lateral entorhinal cortex, may contribute to associative memory as well (Supplementary Fig. 11). One possible scenario is that neural activity in line with the ‘coactivity hypothesis’ leads to the emergence of neural activity proposed by the ‘conjunctive hypothesis’. For instance, coactivity in object and place cells may induce conjunctive object–place coding in downstream neurons, potentially with the help of plateau potentials^54^.

### Hippocampal ripples and cognition

Previous work suggests that hippocampal ripples serve multiple cognitive functions, including memory encoding, consolidation and retrieval^12–14^. For example, findings in rodents demonstrated that the suppression of sharp-wave ripples during post-training sleep impairs spatial memory^55^, providing evidence for a role of ripples in memory consolidation. Studies in humans showed that ripple rates increase when individuals encode new memories^25,28^ and when they freely recall memories^25,28,41^, implicating ripples in both memory encoding and retrieval. Ripple-associated place cell sequences depict the animal’s future paths through an environment, which indicates that hippocampal ripples help plan future behavior^13,30,31^.

In the present study, we extended the evidence for important roles of hippocampal ripples in cognition by examining their possible involvement in human associative memory. Specifically, hippocampal ripples were associated with broad increases in neural activity across the human MTL (Supplementary Fig. 8) and appeared to define time windows for coactive spiking by stimulus-specific neurons that represented different types of information. During the retrieval of object–location memories, we observed this ripple-locked coactivity when the participant was asked to recall the location of the preferred object of the object cell and in which the participant’s response location was inside the place field of the place cell. This result implicates human ripples in the retrieval of associative memories and also supports their implication in planning future behavior, as the coactivity occurred on trials in which the participant’s response location, which is the location that the participant was heading to, was located inside the place field of the place cell. As we observed significant coactivations only during late ripples of each session, it suggests that participants first had to establish some intuition about the locations of the different objects before ripple-locked coactivity during retrieval could emerge.

During re-encoding periods of our object–location memory task, we observed coactivity of object and place cells on trials when the participant was asked to re-encode the correct location of the preferred object of the object cell and when the correct location of the object was inside the place field of the place cell. This coactivity of object and place cells during re-encoding may thus have helped the participants to build or stabilize accurate associations between the different objects and their locations, implicating ripples in memory formation. Notably, we again observed significant ripple-locked coactivity of object and place cells only during late ripples of the recording sessions. We therefore propose that ripple-locked coactivity during re-encoding relates more closely to the stabilization, updating or early consolidation of associative memories rather than to their initial formation^6,12,56^. The differences among memory initialization, stabilization, updating and early consolidation are not exactly defined, however, and hippocampal ripples may be involved in all of these cognitive operations^14^. Future studies may present individuals with various versions of a task that differ with regard to their duration and complexity to clarify the exact role of ripple-locked cellular coactivity for memory. Furthermore, going beyond the correlational nature of our results, future studies may manipulate ripple-locked coactivations to better understand to what extent they are causally relevant.

Our results suggest that the ripple-locked timing of neuronal coactivity shifts between behavioral states. During retrieval, the strongest object cell–place cell coactivity occurred shortly after the hippocampal ripple peaks, whereas, during re-encoding, the coactivations mainly preceded them. This timing shift indicates some flexibility in the interaction between MTL neurons and hippocampal ripples and may potentially reflect task-related changes in the direction of information flow during hippocampal ripples. Ripples during retrieval may induce the coactivity of stimulus-specific neurons and may support the propagation of information from the hippocampus to extra-hippocampal regions. Conversely, during re-encoding, ripples may be triggered by stimulus-specific neuronal activity and may be involved in information transfer from extra-hippocampal regions to the hippocampus. This interpretation is speculative, however, and requires further investigation. Our finding of object cell–place cell coactivity starting before hippocampal ripples is in line with results in rodents showing that place cell sequences can start approximately 100 ms earlier than the hippocampal ripple^29,30^ and that the reactivation of brain-wide neural representations of word pairs, faces and scenes emerges already approximately 100 ms before hippocampal ripples in humans^25,26^. This temporal delay raises the question of whether hippocampal ripples actually trigger the (re)activation of stimulus-specific neural representations or whether another mechanism—for example, the excitatory sharp wave^13,14^—is in fact the trigger event for both the (re)activation of stimulus-specific neural representations and the hippocampal ripples. Sharp waves, which rodent studies typically record from the CA1 stratum radiatum during ripples in the stratum pyramidale^13,34^, reflect the excitatory depolarization of the apical dendrites of pyramidal cells and are presumably generated by the hippocampal CA3 region when the suppression effects of subcortical neuromodulators are removed^13^. It is conceivable that sharp waves rather than ripples are the mesoscopic events that drive cellular coactivations, and further studies are required to understand whether ripple-locked coactivity is better described as being triggered by sharp waves.

Another way in which our study implicates hippocampal ripples in human associative memory is by showing that ripple rates correlated with the participants’ behavioral state and memory performance. Ripple rates increased during memory cues before good memory retrieval and during re-encoding periods after poor memory retrieval. These observations extend previous reports that hippocampal ripples are important for the accurate encoding and retrieval of human memories^23,25–28,41^ and help translate the functional role of ripples from the rodent to the human brain^34^. Of note, we recorded ripples solely from the anterior hippocampus, and future studies are needed to investigate whether ripples in different parts of the human hippocampus have similar or different functional roles (Supplementary Fig. 6). Furthermore, there is an ongoing debate whether the high-frequency events that we and others identified as ‘ripples’ are indeed the human homolog of rodent ripples^34^. Their relation to and/or overlap with gamma oscillations^38^, epsilon oscillations^37^, high gamma^35^, broadband gamma^36^ and pathological ripples^13^ is non-trivial and requires further investigation (Supplementary Fig. 6). As an attempt to ensure that the signals that we identified are true ripples, we required each event to exhibit a minimum of three cycles and a global peak in the power spectrum that falls into the ripple band. Despite these efforts, it may be that the ripples in our study are broadband gamma events, which have been shown to result from enhanced synaptic and spiking activity^57^. Future studies are needed to better understand the diversity of human high-frequency events. Moving forward, these insights may have clinical relevance, as augmenting ripples^32^ might be a target for improving cognitive performance in patients with memory disorders.

### Limitations of the study

It remains unclear whether ripple-locked coactivity has a causal role in the formation of new synaptic connections between neuronal assemblies or whether it is a side effect of more fundamental processes. Future studies may, thus, monitor modulatory brain systems, including neurotransmitters (for example, glutamate), to test whether these systems are driving factors of ripple-locked coactivity. Given the known effects of these neurotransmitters on neural excitability and plasticity, it may be that they simultaneously enhance ripples and neuronal activity, thus constituting the actual origin of strengthened synaptic connections between stimulus-specific neurons that may underlie the formation of new associative memories. It also remains elusive whether ripple-locked coactivity reflects direct synaptic connections between the participating object and place cells or whether it is a byproduct of shared input to the hippocampal formation. Upstream mechanisms of associative memory may activate both object and place cells in a temporally coordinated manner, resulting in coactivations between these cell types without direct connections between them.

Other open questions arise from our computation of cellular coactivations in relatively broad time windows of 100 ms. This temporal resolution occupies an intermediate level between typical studies of synaptic plasticity, in which cellular synchrony is estimated with millisecond precision using, for instance, spike cross-correlations, and studies of correlated trial-to-trial fluctuations of neuronal activity (‘noise correlations’), in which firing rates are usually estimated in second-long time windows^58^. It remains unclear whether and how cellular coactivations at time scales of ~100 ms may support synaptic plasticity, as the time windows for spike-timing-dependent plasticity are typically less than a few tens of milliseconds^59^. To answer this question, it may be useful to consider the possibility of a more relaxed time window for spike-timing-dependent plasticity in humans^60^ and other forms of synaptic plasticity, including behavioral time scale synaptic plasticity^43,44^.

## Methods

Experimental procedures were approved by the Ethics Committee of the University of Freiburg in Freiburg im Breisgau, Germany, and all participants provided written informed consent. Data collection and analysis were not performed blinded to the conditions of the experiment. No statistical methods were used to pre-determine sample sizes, but our sample sizes are similar to those reported in previous publications^9,25,61,62^.

### Human participants

We tested *n* = 35 human participants, who were patients with epilepsy undergoing treatment for pharmacologically intractable epilepsy at the Freiburg Epilepsy Center in Freiburg im Breisgau, Germany. Of these, five patients had to be excluded because of technical issues (*n* = 1); no hippocampal electrode contacts (*n* = 2); hippocampal channels that were close to the resection border of a previous surgery (*n* = 1); and a very low number of ripples (*n* = 1). This resulted in a final sample of *n* = 30 patients (16 female; age range, 19–61 years; mean age ± s.e.m., 36 ± 2 years), contributing a total of *n* = 41 experimental sessions with intracranial EEG recordings including the left and/or right hippocampus (*n* = 62 hippocampal bipolar channels). For 20 of these 30 patients, additional single-neuron recordings from various MTL regions were available (*n* = 27 sessions and *n* = 43 hippocampal bipolar channels). All participants were in the same experimental group and did not undergo randomization. Participants were not compensated for their participation in the study. Further participant information is presented in Supplementary Table 1.

### Neurophysiological recordings

Participants were surgically implanted with intracranial depth electrodes in the MTL for diagnostic purposes to isolate their epileptic seizure focus for potential subsequent surgical resection. The exact electrode numbers and locations varied across participants and were determined solely by clinical needs. Electrodes were provided by Ad-Tech. Macroelectrode recordings were performed at a sampling rate of 2 kHz using a Compumedics system. Microelectrode recordings were performed using Behnke–Fried electrodes (Ad-Tech) at a sampling rate of 30 kHz using a NeuroPort system (Blackrock Microsystems). Each Behnke–Fried electrode contained a bundle of nine platinum–iridium microelectrodes with a diameter of 40 µm that protruded from the tip of the macroelectrode^63,64^ (Fig. 2d). The first eight microelectrodes were used to record action potentials and LFPs. The ninth microelectrode served as reference. Microelectrode coverage included amygdala, entorhinal cortex, fusiform gyrus, hippocampus, insula, parahippocampal cortex, temporal pole and visual cortex. Temporal pole refers to a broader area in the anterior temporal lobe, situated ventral and anterior to the amygdala and entorhinal cortex.

### Associative object–location memory task

During the invasive neural recordings, participants sat in their hospital bed and performed an associative object–location memory task in a virtual environment on a laptop computer (Fig. 1), which was adapted from previous studies^9,65–67^. Before performing the first task session, participants had never been exposed to this task before. The fact that they performed above chance already early during the task (Fig. 1d) is due to the initial encoding period in which they collected each object from its correct location once, giving the participants a rough idea where the objects were placed in the environment. The task was developed using Unreal Engine 2 (Epic Games).

During the task, participants first learned the locations of eight everyday objects by collecting each object from its location once (by running over it). The navigation path of an example participant during this initial encoding period is shown in Supplementary Fig. 3e. This resulted in eight initial encoding events per session (one per object–place association). On average, the initial encoding periods had a duration of 3.326 ± 0.352 minutes (mean ± s.e.m.; Supplementary Fig. 3f). Because of the low number of initial encoding events (eight per session) and the short duration of the initial encoding period, we did not consider them as a focus of our analyses a priori. Basic analyses on average ripple rates during the initial encoding period showed that they were positively correlated with the average ripple rates during the main task (Pearson’s *r* = 0.797, *P* < 0.001, *n* = 62 channels; Supplementary Fig. 3g) and that they were not significantly different from those during the main task (paired *t*-test: *t*(61) = 0.760, *P* = 0.450). When we analyzed time-resolved ripple rates around the initial encoding events, we did not find significant ripple rate increases at particular moments relative to these events (Supplementary Fig. 3h). This observation is similar to the null effect toward the end of the re-encoding phase (Fig. 3c) and may again result from the self-paced nature of the task, due to which actual encoding processes may be less closely locked to particular task events than in tasks that are temporally more tightly controlled (for example, see ref. ^25^ for ripple rate increases when individuals encoded novel pictures of faces or places).

After the initial encoding period, participants completed variable numbers of test trials depending on compliance. Each test trial started with an ITI of 3–5-s duration (uniformly distributed). Participants were then shown one of the eight objects (‘cue’; duration of 2 s). During the subsequent retrieval period (‘retrieval’; self-paced), participants navigated to the remembered object location and indicated their arrival with a button press. Next, participants received feedback on the accuracy of their response using one of five different emoticons (‘feedback’; duration of 1.5 s). The retrieved object then appeared in its correct location, and participants collected it from there to further improve their associative object–location memories (‘re-encoding’; self-paced). Across all trials, the average duration of the retrieval periods was 13.415 ± 0.218 s (mean ± s.e.m.), and the average duration of the re-encoding periods was 7.685 ± 0.158 s (mean ± s.e.m.). The participants could use several different strategies to retrieve the locations of the objects, including allocentric, egocentric and landmark-based strategies^9^. The choice of this object–location memory task, including self-paced navigation in a virtual environment, was guided by previous human single-neuron studies showing single-neuron responses to objects and spatial locations in different regions of the human MTL (for example, see refs. ^10,61^). The task mimicked the setup of navigation studies in rodents that showed robust spatial coding in the rodent hippocampus and neighboring regions (for example, see ref. ^48^) and gave the patients a sense of movement through space. Due to the self-paced nature of the task, the task was engaging and presumably enhanced the presence of neurons being tuned to different aspects of the task. The use of emoticons as feedback may have strengthened single-neuron responses in the amygdala, which have previously been shown to be involved in integrating spatial and motivational information in monkeys^68,69^.

The virtual environment comprised a grassy plain with a diameter of approximately 10,000 virtual units (vu), surrounded by a cylindrical cliff. There were no landmarks within the environment. The background scenery comprised a large and a small mountain, clouds and the sun. All distal landmarks were rendered at infinity and remained stationary throughout the task. Participants were asked to complete up to 160 trials but were instructed to pause or quit the task whenever they wanted. Participants navigated the virtual environment using the arrow keys of the laptop computer (forward, turn left and turn right). Instantaneous virtual locations and heading directions (which are identical to viewing directions in our task) were sampled at 50 Hz. We aligned the behavioral data with the macroelectrode and microelectrode data using visual triggers, which were detected by a phototransistor attached to the screen of the laptop computer. The phototransistor signal was recorded together with the macroelectrode and microelectrode data at temporal resolutions of 2 kHz and 30 kHz, respectively.

### General information on statistics

All analyses were carried out in MATLAB 2020b and 2021b (MathWorks) using MATLAB toolboxes, the CircStat toolbox^70^ (version 1.21.0.0), FieldTrip (version 20210614; http://fieldtriptoolbox.org)^71^ and custom MATLAB scripts.

Unless otherwise indicated, we considered results statistically significant when the corresponding *P* value fell below an alpha level of α = 0.05. Analyses were two-sided, unless otherwise specified. Binomial tests evaluated the significance of proportions of neurons relative to a chance level of 5%, unless otherwise specified. Surrogate statistics were one-sided to assess whether an empirical test statistic significantly exceeded (or fell below) a distribution of surrogate statistics, unless otherwise specified. The use of statistical tests with surrogate statistics avoided the assumption of normality when evaluating significance. When using parametric statistical tests, data distributions were assumed to be normal, but this was not formally tested. Correction for multiple comparisons was applied when necessary. All cluster-based permutation tests controlled for multiple comparisons across all relevant data dimensions. If not otherwise specified, we plotted the following information in box plots: median as center line; upper and lower quartiles as box limits; minimum and maximum (after removing the outliers) as whiskers; and outliers as points. Outliers were identified as elements more than 1.5 interquartile ranges above the third quartile or more than 1.5 interquartile ranges below the first quartile.

### Behavioral analysis

For each trial, we quantified the participant’s associative memory performance by calculating the Euclidean distance between the participant’s response location and the object’s correct location (‘drop error’). Drop errors were transformed into memory performance values by ranking each drop error within a distribution of surrogate drop errors (*n* = 10^7^ surrogate drop errors). Surrogate drop errors were generated synthetically as the distances between the trial-specific correct object location and random locations within the virtual environment. The transformation into memory performance values accounted for the fact that the possible range of drop errors is smaller for objects located in the center of the virtual environment as compared to objects located in the periphery of the virtual environment^9,72^: For objects in the environment center, the possible drop errors are in the range between [0, *r*], whereas they are in the range between [0, 2 × *r*] for objects at the boundary of the arena, where *r* is the arena radius. For objects placed anywhere else in the environment, the drop error is somewhere between the ranges [0, *r*] and [0, 2 × *r*]. Using the transformation procedure, performance values are mapped onto a range between [0, 1], irrespective of whether the associated objects are located in the center or the periphery of the environment. A memory performance value of 1 represents the smallest possible drop error, whereas a memory performance value of 0 represents the largest possible drop error. A given drop error leads to higher memory performance values the closer the correct object location is toward the boundary of the environment. Supplementary Fig. 2 shows an illustration of the procedure of converting drop errors into memory performance values using three examples in which the correct object location is in the center of the environment, at its boundary or halfway between the center and the boundary.

To quantify performance increases within sessions, we computed the change in memory performance between early and late trials (averaged across trials), where early trials are trials 1 to *n* / 2 of a session; late trials are the remaining trials; and *n* is the total number of trials. We observed a significant increase in memory performance across all participants and also when considering only participants with microelectrodes (Supplementary Table 2). We also estimated memory performance values per normalized time bin (20 bins, averaged across trials falling into a given normalized time bin) and calculated a Pearson correlation between bin index and average memory performance afterwards (averaged across sessions).

### Intracranial EEG: pre-processing

Intracranial macroelectrode recordings were performed to identify ripples on hippocampal channels and to examine the MTL-wide effects of hippocampal ripples on LFPs. Signals were sampled at 2 kHz, and initial recordings were referenced to a common surface EEG contact (Cz). We visually inspected the data from each channel and removed channels without reasonable signals (for example, because they were located outside the brain). This resulted in a total number of 2,897 intracranial EEG channels across all 41 sessions (519 out of 3,416 channels were removed because of artifactual data).

To eliminate potentially system-wide artifacts or noise and to better sense ripples locally, we then applied bipolar re-referencing between pairs of neighboring contacts^26,41,73^. After bipolar re-referencing, we used band-stop filters to perform line noise removal at 50, 100, 150 and 200 Hz (±2 Hz; two-pass 4th-order Butterworth filter) in FieldTrip. To remove time periods with ripple-like artifacts that were present on the majority of all intracranial EEG channels, we computed the grand average signal across all intracranial EEG channels^25,74^. This grand average signal was also band-stop filtered at 50, 100, 150 and 200 Hz to remove line noise.

### Intracranial EEG: electrode locations

To identify which bipolar channels were located inside the hippocampus and could, thus, be used to detect hippocampal ripples, we visually inspected all hippocampal electrodes on the post-implantation magnetic resonance imaging (MRI) scans (Supplementary Fig. 4). Following the hippocampal segmentation in ref. ^25^, we assigned the hippocampal bipolar channels to putative hippocampal subregions, which suggested that most bipolar channels were located in CA1. We similarly inspected all amygdala, entorhinal cortex, parahippocampal cortex and temporal pole electrodes to identify which bipolar channels were located in these regions (to examine the MTL-wide effects of hippocampal ripples). The locations of all bipolar channels in the different regions are displayed in Supplementary Fig. 8a.

To show a summary of all hippocampal bipolar channels, we determined the location of each macroelectrode channel in MNI space using PyLocator (http://pylocator.thorstenkranz.de/), following our previous procedure^75^, and estimated the location of each bipolar channel as the mean of the MNI coordinates of the two contributing channels. We display the distribution of all hippocampal bipolar channels as a probability map on the group average MRI scan (Fig. 2b).

### Intracranial EEG: IEDs

To reduce the probability that the detected ripples were a result of IEDs^73^, we identified IEDs before ripple detection using an automated procedure, which we double-checked with visual inspection. We automatically detected IEDs following previously established methods^23,24,33^. The raw data were filtered using a high-pass filter of 0.5 Hz (two-pass 5th-order Butterworth filter) to remove slow-frequency drifts and a low-pass filter of 150 Hz (two-pass 6th-order Butterworth filter) in FieldTrip. A timepoint was considered belonging to an IED if (1) its amplitude exceeded four interquartile ranges above or below the median amplitude calculated across the entire recording; if (2) the gradient to the next timepoint exceeded four interquartile ranges above or below the median gradient; or if (3) the sum power across the frequencies 1–60 Hz (30 logarithmically spaced frequencies; time–frequency decomposition using Morlet wavelets with seven cycles, followed by taking the natural logarithm and frequency-specific *z*-scoring across time) exceeded four interquartile ranges above the median sum power. The rationale behind these criteria was that IEDs exhibit high amplitudes, sharp amplitude changes and power increases across a broad frequency range. We used interquartile ranges instead of standard deviations to reduce the influence of outliers. We inspected the output of our automated IED detection, which appeared suitable for detecting IEDs. Example IEDs are shown in Supplementary Fig. 5a.

### Intracranial EEG: relationship between IEDs and behavior

To investigate systematic relationships between hippocampal IEDs and behavior, we estimated the prevalence of IEDs per trial phase. Using a repeated-measures ANOVA (dependent variable: IED prevalence, averaged across trials; independent variable: trial phase; Tukey–Kramer correction for multiple comparisons), we tested whether the occurrence of IEDs varied as a function of trial phase.

To understand whether IEDs were associated with memory performance, we performed partial correlations between IED prevalence and memory performance values across trials, separately for each trial phase (controlling for trial index and the interaction between memory performance and trial index). We then computed one-sample *t*-tests across channels to see whether the correlations were significantly above or below 0, including Bonferroni correction for the number of trial phases. Moreover, to test whether IEDs increased or decreased over the course of the task, we performed partial correlations between IED prevalence and trial indices across trials, separately for each trial phase (controlling for memory performance and the interaction between memory performance and trial index). To corroborate the results from the partial correlations, we also computed linear mixed models with IED prevalence as dependent variable and various independent variables (Supplementary Table 3).

### Intracranial EEG: ripples

We detected hippocampal ripples on bipolar channels of hippocampal macroelectrodes. If a participant was implanted bilaterally, hippocampal channels from both hemispheres were used for ripple detection. We first ensured reasonable signals on each hippocampal ripple channel by visually inspecting the raw intracranial EEG traces during pre-processing. If the signal of the most medial bipolar hippocampal channel did not have sufficient quality (which was the case in five of the 62 hippocampal channels), we used the second-most medial bipolar hippocampal channel for ripple detection. In one participant, the hippocampal electrode was implanted from posterior to anterior along the longitudinal axis of the hippocampus—in this case, we selected the two most anterior hippocampal channels so that the resulting bipolar channel was located in a similar hippocampal subregion as the channels from all other participants (that is, in the anterior hippocampus). For each bipolar hippocampal channel, we visually verified that it was located inside the hippocampus. Most hippocampal channels were putatively located in the CA1 region (Supplementary Fig. 4). In total, 62 hippocampal channels were included (33 from the right hemisphere). Forty-three of these channels were from participants with microelectrode recordings.

Ripple detection was preceded by a detection of IEDs (see above) and ripple-like events in the grand average signal to exclude those time periods from ripple detection. To reduce the probability that the detected ripples were a result of artifacts, we conservatively excluded ±1 s around each detected IED^24^ and each timepoint that co-occurred with a ripple-like event in the grand average signal.

To detect ripple candidates, we filtered the raw LFP between 80 Hz and 140 Hz (two-pass 4th-order Butterworth filter) and computed the instantaneous analytic amplitude within that band using a Hilbert transform^26,73^. We then smoothed the amplitudes using a smoothing time window of 20 ms^33^. Timepoints with artifacts were excluded (that is, set to NaNs) in the smoothed amplitude time series. Next, we computed the mean and standard deviation of the smoothed amplitudes across the entire recording and detected candidate ripple events as time periods in which the signal exceeded 2 standard deviations above the mean^26,27,73^. Each candidate event then had to fulfill additional criteria to qualify as a putatively physiological ripple: (1) the peak of the smoothed amplitude had to exceed 3 standard deviations above the mean^26,73^; (2) the duration had to last longer than 20 ms (ref. ^25^) and be shorter than 500 ms^27^; (3) the band-pass signal needed to have at least three peaks and at least three troughs^33^; and (4) the power spectrum, computed for frequencies between 30 Hz and 190 Hz in steps of 2 Hz (using Morlet wavelets with seven cycles) and divided by the power spectrum estimated across the entire recording, had to exhibit a global peak between 80 Hz and 140 Hz. Only candidate events that fulfilled all these criteria were considered as ripples.

Next, for each ripple, we extracted its peak time as the timepoint at which the band-pass signal was highest. Ripple duration was defined as the time difference between the start and end time of a given ripple. The frequency of a ripple was estimated based on the average temporal delay between the peaks and troughs in the band-pass signal. To show the time domain signal, the frequency domain power spectrum (2–200 Hz in steps of 4 Hz) and the time–frequency domain power spectrogram of the ripples (2–200 Hz in steps of 4 Hz), we extracted the raw LFP within ±3 s around each ripple. Supplementary Fig. 6 shows various ripple characteristics, including ripple rates, durations, frequencies, power spectra and inter-ripple intervals.

To compare the putatively physiological ripples against an identical number of ‘surrogate ripples’ (Fig. 2i and Supplementary Fig. 6), we selected a random timepoint within ±60 s of each putatively physiological ripple (excluding time periods with artifacts), which we denoted as the peak time of the corresponding surrogate ripple^33^.

### Intracranial EEG: delta phase locking of hippocampal ripples

To investigate whether ripples were locked to particular phases of low-frequency oscillations^15,23^, we filtered the hippocampal intracranial EEG with a two-pass finite impulse response (FIR) filter using MATLAB’s designfilt and filtfilt functions (filter order, 8,000; lower cutoff frequency, 0.5 Hz; upper cutoff frequency, 2 Hz). We then estimated the phases of this delta band filtered data using a Hilbert transform. For each ripple, we extracted its corresponding delta phase at the ripple peak time and averaged the ripple-locked delta phases afterwards. Across channels, we tested whether the average delta phases were clustered using a Rayleigh test^70^.

To assess statistical significance of ripple–phase coupling, we compared the empirical Rayleigh *z* value against 1,001 surrogate Rayleigh *z* values, which we generated by performing the same steps as described above with the only difference that the inter-ripple intervals were randomly shuffled. We computed the *P* value of the empirical Rayleigh *z* value in comparison to the surrogate Rayleigh *z* values as *P* = 1 − *rank*, where *rank* is the fraction of surrogate Rayleigh *z* values that were smaller than the empirical Rayleigh *z* value. This analysis showed that the delta phase locking of empirical ripples was significantly stronger than in surrogate ripples with shuffled inter-ripple intervals (*P* = 0.017). To also test whether the empirical average delta phases (one per channel) were different from the surrogate average delta phases (one per channel in each of 1,001 surrogate rounds), we performed a two-sample Kuiper’s test^70^. This showed that the empirical preferred delta phases were significantly different from the preferred delta phases of surrogate ripples (Kuiper’s test: *k* = 1,132,988.000, *P* = 0.001).

### Intracranial EEG: extra-hippocampal ripple detection

To characterize the MTL-wide effects of hippocampal ripples, we detected ripples on bipolar channels from the amygdala, entorhinal cortex, parahippocampal cortex and temporal pole using the same procedure as for hippocampal ripples (see above). We then performed cross-correlations between the ripple time series of a given hippocampal channel and the ripple time series of another channel (where each ripple time series is a vector of zeros and ones, with values of 1 indicating ripple periods). We considered maximum time lags of ±5 s between both time series and used unbiased estimations of the cross correlations by means of MATLAB’s ‘xcorr’ function. We smoothed the pairwise cross-correlations with a Gaussian filter (kernel length of 0.2 s) and *z*-scored the cross-correlations across time lags. To evaluate whether the *z*-scored cross-correlations were significantly positive, we then performed a cluster-based permutation test against 0 across channels (1,001 surrogates).

In this cluster-based permutation test, we first applied a one-sample *t*-test to the empirical data, separately for each time lag, and identified contiguous clusters of time lags in which the uncorrected *P* value of the *t*-test was significant (α = 0.05) and the *t* value was positive. For each cluster, we computed an empirical cluster statistic by summing up all *t* values that were part of that cluster (*t*_cluster-empirical_). We then compared the empirical cluster statistics against surrogate cluster statistics, which we obtained by inverting the sign of the cross-correlation values of a random subset of the cross-correlation series^72^, performing exactly the same steps as described above for the empirical data and keeping only the maximum cluster statistic (resulting in 1,001 *t*_max-cluster-surrogate_ values). We considered an empirical cluster statistic (*t*_cluster-empirical_) significant if it exceeded the 95th percentile of all surrogate maximum cluster statistics (*t*_max-cluster-surrogate_).

### Intracranial EEG: LFP power during hippocampal ripples

To characterize the MTL-wide effects of hippocampal ripples, we computed ripple-aligned time–frequency-resolved power spectrograms in different MTL regions. Across the 41 sessions with macroelectrode recordings, 400 combinations of electrodes in the left/right hippocampus and electrodes in the left/right amygdala, left/right entorhinal cortex, left/right hippocampus, left/right parahippocampal cortex or left/right temporal pole were available (240 in ipsilateral and 160 in contralateral hemispheres). Forty-five macroelectrode channels were located in the left amygdala (26 ipsilateral to their co-recorded hippocampal channel), 48 in the right amygdala (29 ipsilateral), 11 in the left entorhinal cortex (seven ipsilateral), 33 in the right entorhinal cortex (21 ipsilateral), 50 in the left hippocampus (29 ipsilateral), 54 in the right hippocampus (33 ipsilateral), 33 in the left parahippocampal cortex (18 ipsilateral), 31 in the right parahippocampal cortex (20 ipsilateral), 45 in the left temporal pole (26 ipsilateral) and 50 in the right temporal pole (31 ipsilateral).

For each macroelectrode channel, we computed the time–frequency spectrogram across the entire recording (using Morlet wavelets with seven cycles at 50 logarithmically spaced frequencies between 1 Hz and 200 Hz). Power values at timepoints with IEDs were excluded (that is, set to NaN). Power values were then *z*-scored across time (using MATLAB’s ‘normalize’ function), separately for each frequency. Around each hippocampal ripple (±3 s), we extracted the *z*-scored power values, averaged across ripples in each channel and smoothed the average spectrograms with a Gaussian filter across time (kernel length, 0.2 s). Spectrograms were then truncated to ±0.5 s around the ripple peak timepoint. Next, we averaged the *z*-scored power spectrograms across channels for depiction and performed cluster-based permutation tests (1,001 surrogates) across channels to statistically evaluate whether hippocampal ripples were associated with significant changes in LFP power in other MTL regions.

In these cluster-based permutation tests, we first applied a one-sample *t*-test to the empirical data, separately for each time–frequency bin, and identified contiguous clusters of time–frequency bins in which the uncorrected *P* value of the *t*-test was significant (α = 0.025). For each cluster, we computed an empirical cluster statistic by summing up all *t* values being part of that cluster (*t*_cluster-empirical_). We then compared the empirical cluster statistics against surrogate cluster statistics, which we obtained by flipping the sign of the power values of a random subset of the power spectrograms, performing exactly the same steps as described above for the empirical data and keeping only the maximum cluster statistic (resulting in 1,001 *t*_max-cluster-surrogate_ values). We considered an empirical cluster statistic (*t*_cluster-empirical_) significant if it exceeded the 97.5th percentile or if it fell below the 2.5th percentile of all surrogate maximum cluster statistics (*t*_max-cluster-surrogate_). We used a first-level alpha of α = 0.025 (for identifying significant time–frequency bins in the power spectrograms) and a second-level alpha of α = 0.025 (for identifying significant clusters of significant time–frequency bins), because these cluster-based permutation tests tested for both increases and decreases in power.

### Intracranial EEG: relationship between hippocampal ripples and behavior

Previous studies demonstrated that characteristics of hippocampal ripples can vary between different cognitive tasks, between different components of the same task and as a function of the participants’ behavioral performance^23,25–28,41,73,74^. Hence, we aimed at understanding whether hippocampal ripples were related to the participants’ behavioral state and memory performance in our object–location memory task.

To test whether ripple characteristics varied as a function of trial phase (that is, ITI, cue, retrieval, feedback and re-encoding), we estimated their rate, duration and frequency in each trial phase. We then tested for significant associations between ripple characteristics and behavior using repeated-measures ANOVAs (dependent variable: ripple characteristic, averaged across trials; independent variable: trial phase; Tukey–Kramer correction for multiple comparisons) and linear mixed models (Supplementary Tables 4 and 5). In line with previous results^25,27^, ripple rates were increased during the ITI and cue periods, when participants rested and viewed pictures of the objects that cued them to remember particular locations in the environment, respectively (repeated-measures ANOVA: *F*_4,244_ = 19.942, *P* < 0.001; post hoc comparisons between ITI and retrieval, feedback or re-encoding: all *P*_Tukey–Kramer_ < 0.024; post hoc comparisons between cue and retrieval, feedback or re-encoding: all *P*_Tukey–Kramer_ < 0.001; Fig. 3a). Ripple durations showed a similar modulation pattern (repeated-measures ANOVA: *F*_4,236_ = 2.463, *P* = 0.046; Fig. 3a), but post hoc comparisons were non-significant (all *P*_Tukey–Kramer_ > 0.138). Ripple frequency was not modulated by trial phase (repeated-measures ANOVA: *F*_4,236_ = 0.562, *P* = 0.690; Fig. 3a). These results demonstrate that the rate of human hippocampal ripples changed with the participants’ behavioral state in our associative object–location memory task.

We next examined the memory relevance and temporal stability of hippocampal ripple rates. To test whether ripple rates were correlated with memory performance, we computed a partial correlation between trial-wise ripple rate and memory performance for each channel, separately for each trial phase (controlling for trial index and the interaction between memory performance and trial index). Across channels, we then tested whether the correlation values were significantly different from 0 using one-sample *t*-tests including Bonferroni correction for the number of trial phases. During the cue period, ripple rates correlated positively with memory performance—that is, with more frequent ripples predicting better memory performance in the subsequent retrieval period (one-sample *t*-test against 0: *t*(60) = 2.763, *P* = 0.038, Bonferroni corrected for five tests; Fig. 3b). During re-encoding, ripple rates correlated negatively with memory performance, meaning that higher ripple rates followed memory responses with lower memory performance (one-sample *t*-test against 0: *t*(60) = −4.181, *P* < 0.001, Bonferroni corrected for five tests; Fig. 3b). This result implicates hippocampal ripples in the formation, or updating, of associative memories as participants corrected/updated their memories by viewing the objects in their true locations during the re-encoding periods.

We also tested whether ripple rates were associated with trial index (that is, time within the task) using partial correlations as described above for memory performance (controlling for memory performance and the interaction between memory performance and trial index). This showed that ripple rates were largely stable across the course of the experiment, with a decrease of ripples over time during the feedback period (one-sample *t*-test against 0: *t*(59) = −3.178, *P* = 0.012, Bonferroni corrected for five tests; Fig. 3d).

To corroborate these results from the partial correlations (Fig. 3), we performed follow-up analyses using linear mixed models where we also controlled for the trial-wise prevalence of artifacts and obtained qualitatively identical results (Supplementary Tables 4 and 5). In these linear mixed models, ripple rates were entered as the dependent variable, and memory performance, trial index and trial phase were modeled as fixed effects. We included channel index as a random effect. In the second linear mixed model (Supplementary Table 5), the trial-wise and phase-wise prevalence of artifacts (that is, IEDs and ripple-like events in the grand average signal) was included as a covariate. Both linear mixed models showed that ripple rates were higher during the cue period and lower during the retrieval, feedback and re-encoding periods as compared to the ITI period. As compared to the ITI period, ripple rates and memory performances were more positively correlated with each other during the cue period and more negatively correlated with each other during the re-encoding period. Furthermore, ripple rates during the cue period and the feedback period decreased with increasing trial index.

To describe the time course of ripple rates during the trial phases, we computed peristimulus time histograms for the occurrence of ripples relative to the start and end time of the different trial phases (that is, relative to the start for the cue and the feedback periods and relative to the end for the ITI, retrieval and re-encoding periods). For each hippocampal ripple channel, we computed the time course of instantaneous ripple rates during trials with good memory performance and bad memory performance (based on a median split of each participant’s memory performance values) and used two-sided cluster-based permutation tests in FieldTrip to evaluate whether time-resolved ripple rates changed during particular periods of the different trial phases between trials with good versus bad memory performance. Although the ITI, retrieval and re-encoding periods had variable durations across trials, we restricted our analysis of their time-resolved ripple rates to −3 s to +1 s relative to the end of these periods to examine ripple rates as a function of absolute time.

These time-resolved analyses showed that, during the cue period, the difference in ripple rates between good and bad trials (defined by a median split of each participant’s memory performance values) was strongest at 1.162–1.470 s after cue onset (cluster-based permutation test: *t*_cluster_ = 1,023.281, *P* = 0.011), whereas the difference during re-encoding was broadly distributed over time (Fig. 3c). Ripple rates did not correlate with memory performance during the retrieval period, and ripple rates did not increase at a fixed interval before successful retrieval (Fig. 3b,c), which is presumably a result of the self-paced nature of the task.

These findings demonstrate that the rate of human hippocampal ripples is associated with the participants’ behavioral state and memory performance in our associative object–location memory task. Increased ripple rates during the cue period preceded the successful retrieval of associative memories, which implicates hippocampal ripples in retrieval processes. Increased ripple rates during the re-encoding period followed the unsuccessful retrieval of associative memories, suggesting an additional role for hippocampal ripples in establishing or updating associative memories. These observations thus extend the previously established links between hippocampal ripples and memory processes in awake humans^23,25–28,41,73,74^.

### Single-neuron recordings: spike detection and sorting

Neuronal spikes were detected and sorted using Wave_Clus 3 (ref. ^76^). We used default settings with the following exceptions^9^: ‘template_sdnum’ was set to 1.5 to assign unsorted spikes to clusters in a more conservative manner; ‘min_clus’ was set to 60 and ‘max_clus’ was set to 10 to avoid over-clustering; and ‘mintemp’ was set to 0.05 to avoid under-clustering. All clusters were visually inspected and judged based on their spike shape and its variance, inter-spike interval (ISI) distribution and the presence of a plausible refractory period. If necessary, clusters were manually adjusted or excluded. Spike waveforms are shown as density plots in all figures. Spike times were aligned to the macroelectrode time axis using the trigger timestamps to investigate the relationship of single-neuron activity to events in the macroelectrode and behavioral data.

In total, we identified *n* = 1,063 clusters (also referred to as ‘units,’ ‘neurons’ or ‘cells’ throughout the manuscript) across 27 experimental sessions from 20 participants who had microelectrodes implanted. We localized the tips of the depth electrodes to brain regions based on post-implantation MRI scans to assign neurons recorded from the corresponding microelectrodes to these regions (for example, Supplementary Fig. 1). We recorded *n* = 340 neurons from the amygdala, *n* = 214 neurons from the entorhinal cortex, *n* = 24 neurons from the fusiform gyrus, *n* = 213 neurons from the hippocampus, *n* = 2 neurons from the insula, *n* = 126 neurons from the parahippocampal cortex, *n* = 135 neurons from the temporal pole and *n* = 9 from the visual cortex. Due to low numbers of neurons in fusiform gyrus, insula and visual cortex, we excluded these regions from region-specific analyses. Fourteen microelectrode participants in this study were also part of a previous study^9^.

For recording quality assessment (Supplementary Fig. 1), we calculated the number of units recorded on each microelectrode (for all microelectrodes with at least one unit); the ISI refractoriness of each unit; the mean firing rate of each unit; and the waveform peak signal-to-noise ratio (SNR) of each unit^9^. The ISI refractoriness was assessed as the percentage of ISIs with a duration of <3 ms. The waveform peak SNR was determined as SNR = A_peak_ /SD_noise_, where A_peak_ is the absolute amplitude of the peak of the mean waveform, and SD_noise_ is the standard deviation of the raw data trace (filtered between 300 Hz and 3,000 Hz).

### Single-neuron recordings: neuronal activity during hippocampal ripples

We were interested in understanding how the firing rates of neurons in various MTL regions behaved during hippocampal ripples (Fig. 4d). Thus, in participants with single-neuron recordings, we computed instantaneous firing rates across the entire experiment (smoothed with a Gaussian filter with a kernel length of 0.2 s) and *z*-scored the firing rates across time. We then extracted the smoothed and *z*-scored firing rates during ±3 s relative to the hippocampal ripple peak timepoints and averaged across ripples afterward (separately for each neuron–ripple channel combination).

To test whether neuronal firing rates were significantly elevated during hippocampal ripples, we performed cluster-based permutation tests (1,001 surrogates). In these cluster-based permutation tests, we first performed a one-sample *t*-test against 0, separately for each time bin. We then identified contiguous clusters of time bins with uncorrected *P* values of *P* < 0.05 and calculated the sum *t* value for each cluster (*t*_cluster-empirical_). To create surrogate cluster statistics, we inverted the sign of a random subset of the neuronal firing rates 1,001 times. Using each set of surrogate data, we performed exactly the same steps as described above (time bin-wise one-sample *t*-tests against 0; identification of contiguous clusters of time bins with uncorrected *P* values of *P* < 0.05; and calculation of the sum *t* value for each cluster). In each surrogate round, we kept the maximum sum *t* value (*t*_max-cluster-surrogate_). We then considered *t*_cluster-empirical_ significant if it exceeded the 95th percentile of all *t*_max-cluster-surrogate_ values. We separately tested for significantly elevated neuronal firing rates during hippocampal ripples depending on the regions in which the neurons were located (Supplementary Fig. 8d).

We also examined the ripple-associated firing rates in different trial phases, separately for trials with good or bad memory performance (Supplementary Fig. 10). We tested whether the ripple-locked firing rates were significantly different between trials with good versus bad memory performance using cluster-based permutation tests (1,001 surrogates) in FieldTrip^71^, by calculating surrogate cluster statistics based on surrogate data that we created by randomly re-assigning ripple-locked firing rates to trials with good or bad memory performance.

### Single-neuron recordings: object cells

We designed the analysis of object cells to identify neurons that exhibited significant firing rate increases in response to one particular object during the cue period. Hence, each object cell had to fulfill two criteria. (1) The absolute average firing rates during cue periods with the cell’s ‘preferred’ object had to be significantly higher than the absolute average firing rates during cue periods with the other, ‘unpreferred’ objects. (2) The time-resolved firing rates during cue periods with the preferred object had to exhibit a significant cluster of timepoints in which the relative firing rates (relative to a 1-s baseline period immediately before the onset of the cue period) were significantly higher than during trials with the unpreferred objects. We used criterion (2) in addition to criterion (1) to ensure that the cell’s preferred object was associated with a circumscribed firing rate increase during the cue period.

To evaluate criterion (1), we performed the following steps. We computed the average firing rate of the cell during each cue period; we designated the object with the highest grand average firing rate as the ‘preferred object’; we performed a two-sample *t*-test between the average firing rates from cue periods with the preferred object versus the average firing rates from cue periods with the unpreferred objects (*t*_empirical_); we created 1,001 surrogate statistics (*t*_surrogate_) by performing the previous steps on randomly shuffled average firing rates (breaking up the assignment between average firing rates and object identity); and we then considered a cell as fulfilling criterion (1) if *t*_empirical_ exceeded the 95th percentile of *t*_surrogate_.

To evaluate criterion (2), we performed the following steps. We computed the time-resolved firing rates of the cell during each cue period (temporal resolution, 0.01 s; smoothing with a Gaussian filter with a kernel length of 0.5 s); we baseline corrected the time-resolved firing rates relative to a 1-s baseline period (immediately preceding the onset of the cue period); and we then used a cluster-based permutation test in FieldTrip^71^ to examine whether there was a time window during the cue period in which the baseline-corrected, time-resolved firing rates were significantly higher during cue periods with the preferred object as compared to cue periods with the unpreferred objects (1,001 surrogates; one-sided α = 0.05). We then labeled a neuron as an object cell if both criteria were fulfilled. The cells’ preferred objects are indicated by orange color in all object cell-related figures.

To characterize object cells in greater detail (Fig. 5b–e), we calculated the percentage of object cells in the different MTL regions. To further understand their tuning, we calculated the sum of all significant time windows across object cells and their average time-resolved firing rates in response to the preferred and unpreferred objects. To examine the temporal stability of their tuning, we estimated each object cell’s time-resolved firing rate in response to the cell’s preferred object in the first and second half of the data and then compared the two tuning curves using a Pearson correlation across time. We present the results regarding the tuning strength and temporal stability of object cells mainly for illustration purposes because these analyses are not fully independent from our procedure of identifying the object cells.

### Single-neuron recordings: place cells

We designed the analysis of place cells to identify cells that exhibited significant firing rate increases when the participant was at a particular location of the virtual environment. Similar to previous single-neuron studies in humans^9,49,61,62,77–79^, the firing rate profiles of our human place cells were less specific than those of place cells in rodent studies, which is why human place cells are sometimes referred to as ‘place-like cells’^9,79^. Our analysis nevertheless ensured that our human place cells exhibited distinct place fields in the virtual environment in which the cells’ firing rates were significantly higher than in the other parts of the environment. The weaker spatial tuning of human place cells is presumably due to several different reasons, including the fact that the patients with epilepsy did not physically navigate but, rather, performed virtual navigation.

To identify place cells in our dataset, we first resampled the behavioral information about the participant’s *x*–*y* position in the environment to a time resolution of 10 Hz (refs. ^9,77^) and calculated the neuronal firing rate (Hz) in each 0.1-s time bin. We then estimated the average firing rate within each bin of a 25 × 25 grid overlaid onto the environment (edge length of 400 vu) and excluded areas of the environment that the participant traversed fewer than two times. Time periods in which the participant did not move or did not turn around for more than 2 s were excluded from this firing rate map (to exclude periods when the participant was idle). The firing rate map was then smoothed with a Gaussian kernel (kernel size, 5; standard deviation, 1.5; using MATLAB’s ‘fspecial’ and ‘conv2’ functions). Next, we thresholded the firing rate map at the 75th percentile of the firing rate values and considered contiguous bins with firing rates above this threshold as candidate place fields. We kept the candidate place field with the highest sum firing rate as the potential place field of this cell. We quantified the strength of this potential place field as the *t*-statistic of a two-sample *t*-test comparing the firing rates when the participant was inside the place field with the firing rates when the participant was outside the place field. We considered the empirical *t*-statistic (*t*_empirical_) significant if it exceeded the 95th percentile of 1,001 surrogate *t-*statistics (*t*_surrogate_), which we obtained by performing exactly the same procedure as described above, with the only difference that we circularly shifted the firing rates relative to the behavioral data by a random lag (with the end of the session wrapped to the beginning), following previous studies (for example, refs. ^9,62^). If *t*_empirical_ was above the 95th percentile of all *t*_surrogate_ values, we considered the place field significant and designated the cell as a place cell.

To empirically estimate the false-positive rate of place cells in our data^9^, we applied this exact analysis procedure to surrogate data (obtained by circularly shifting the firing rates relative to the behavioral data by a random lag with the end of the session wrapped to the beginning). We obtained 4.6% (*n* = 49) statistically significant outcomes (that is, false positives), which confirmed the a priori chosen alpha level of 5%.

To characterize place cells (Fig. 6b–h), we estimated the percentage of place cells in the different MTL regions. We also calculated the cumulative distribution of place fields across the virtual environment (relative to the cumulative distribution of the firing rate maps across the environment) and the size of the place fields across all place cells (expressed as percentages relative to the spatial extent of the firing rate maps). We quantified the ‘peripherality’ of place fields by calculating, for each place cell, the percentage of place field bins that were located at the edge of the firing rate map (relative to the total number of place field bins). The higher this percentage, the more peripheral the place field relative to the firing rate map. We also tested whether place fields were biased toward object locations by counting, for each place field, the number of objects whose locations were inside the place fields. To test for significance, we compared the empirical counts of objects inside place fields with surrogate counts that we obtained by calculating how often the objects were located inside surrogate place fields. For each empirical place field, we created 1,001 surrogate place fields that were of the same size as the empirical place field but covered a different part of the corresponding firing rate map. We furthermore estimated the average firing rate of place cells inside versus outside the place fields and quantified the temporal stability of the place cells by performing a Pearson correlation between the firing rate map estimated using the first half of the data and the firing rate map estimated using the second half of the data. We assessed the statistical significance of the temporal stability values by performing a one-sample *t*-test of the correlation values against 0. We present the results regarding the tuning strength and temporal stability of place cells mainly for illustration because these analyses are not fully independent from our procedure of identifying place cells.

### Single-neuron recordings: conjunctive object–place cells

We defined conjunctive object–place cells as cells that exhibited significant place tuning only during trials with one particular object (but not during trials with any other object). To identify conjunctive object–place cells, we performed the place cell analysis (as described above) eight times for each cell, each time considering only the trials with a particular object (for an example, see Supplementary Fig. 11a). If a cell exhibited significant place tuning at a Bonferroni-corrected alpha level of α = 0.05/8 for exactly one object, we considered the cell as a conjunctive object–place cell.

To characterize conjunctive cells (Supplementary Fig. 11b–d), we estimated the percentage of conjunctive cells in different MTL regions and estimated their overlap with object cells and place cells. To provide supplemental evidence for the fact that conjunctive cells exhibited spatial tuning that was specific to trials with one particular object—instead of demonstrating overall spatial tuning as in place cells—we estimated the pairwise similarity of the spatial firing rate maps between trials with different objects using Pearson correlations (and averaged the similarity values across all pairwise comparisons afterward). We then performed one-sample *t*-tests against 0 to show that these similarity values were not significantly above 0 for conjunctive cells (in line with spatial tuning being specific to trials with a particular object) but significantly above 0 for place cells (in line with spatial tuning being stable across time and, thus, independent of particular objects).

### Single-neuron recordings and intracranial EEG: coactivity of object cells and place cells during hippocampal ripples

Our main hypothesis pertained to the question of whether object and place cells activated together during the same hippocampal ripples. To answer this question, we performed the following analysis. For each neuron–ripple channel combination (*n* = 1,716), we first estimated whether the neuron was active in various time bins relative to the ripple peaks (301 time bins at −0.75 s to 0.75 s relative to the ripple peaks with a bin width of 0.1 s and a step size of 0.005 s; 95% overlap between neighboring time bins). In total, there were 192 neuron–ripple channel combinations in which the neuron was an object cell, and there were 182 neuron–ripple channel combinations in which the neuron was a place cell.

Next, for each simultaneously recorded pair of an object cell and a place cell, we quantified the coactivity of both cells using a previously developed coactivity *z*-score^80^. For a given combination of time bins used to estimate the activations of both cells, we calculated the coactivity *z*-score as:

$$z=\frac{{n}_{{{\mathrm{AB}}}}-\frac{{n}_{{\mathrm{A}}}{n}_{{\mathrm{B}}}}{N}}{\sqrt{\frac{{n}_{{\mathrm{A}}}{n}_{{\mathrm{B}}}\left(N-{n}_{{\mathrm{A}}}\right)\left(N-{n}_{{\mathrm{B}}}\right)}{{N}^{2}\left(N-1\right)}}}$$

where *N* is the total number of ripples; *n*_A_ is the number of ripples in which cell A spiked; *n*_B_ is the number of ripples in which cell B spiked; and *n*_AB_ is the number of ripples in which both cells spiked. An illustration of the coactivity score and simulations regarding the relationship between coactivity scores and cellular firing rates are shown in Supplementary Fig. 13. Because we estimated the activity of each cell at various timepoints relative to the ripple peaks (see above), we were able to compute the coactivity *z*-score for various combinations of time bins (that is, for all possible time bins *i*_A_ and *j*_B_ of cell A and cell B, respectively). By considering all possible time bin combinations, this procedure resulted in a two-dimensional time-by-time coactivity map for each cell pair that showed the cells’ coactivity at various timepoints relative to the hippocampal ripple peaks. Example coactivity maps of individual cell pairs are shown in Supplementary Fig. 17.

We estimated the coactivity maps for different conditions by computing the coactivity *z*-scores across those ripples that occurred during that condition. Specifically, for estimating coactivity during retrieval, we selected those ripples that occurred in the time periods between the onsets of the retrieval phases and the onsets of the feedback phases (across both movement and non-movement periods). To estimate coactivity during re-encoding, we selected those ripples that occurred in the time periods between the onsets of the re-encoding phases and the onsets of the next ITI phases (again across both movement and non-movement periods). We proceeded the same way for all other conditions (for example, for ‘retrieval late ripples’, we only used ripples *n* / 2 + 1 to *n* that occurred during the retrieval periods, where *n* is the total number of ripples).

To statistically evaluate the coactivity maps across all associative object cell–place cell pairs, we used a series of cluster-based permutation tests. During retrieval, associative cell pairs were selected as cell pairs in which the participant’s response location in response to the object cell’s preferred object was inside the place field of the place cell. During re-encoding, associative cell pairs were selected as the pairs in which the correct location of the preferred object of the object cell was inside the place field of the place cell. We used the cluster-based permutation tests to compare the coactivity maps (±0.25 s around the ripple peaks) against chance (denoted as ‘> 0’ or as ‘> surrogates’ in the figure legends); against coactivity maps from a baseline period (0.75 s to 0.25 s before the ripple peaks and 0.25 s to 0.75 s after the ripple peaks, averaged across these two time windows, denoted as ‘> baseline’ in the figure legends); and against the coactivity maps of object cell–place cell pairs encoding non-associative information (denoted as ‘> pref. object and response location outside place field’ or ‘> pref. object and object location outside place field’ in the figure legends). We reasoned that the combination of these three tests would provide robust information about the significance of ripple-locked coactivity of object and place cells.

The different cluster-based permutation tests are illustrated in Supplementary Fig. 14. When using cluster-based permutation tests to compare the coactivity maps against chance, we proceeded as follows. We performed a one-sample *t*-test of the coactivity *z* values against zero across all object cell–place cell–ripple channel combinations, separately for each bin of the two-dimensional time-by-time coactivity maps. We next identified clusters of contiguous bins, for which the uncorrected *P* value was significant (α = 0.05) and the *t*-statistic was above zero (given our a priori hypothesis of increased object cell–place cell coactivity during hippocampal ripples). For each cluster, we then computed the sum of all *t* values (*t*_cluster-empirical_) and compared these empirical cluster statistics against 2,001 surrogate statistics. To obtain each of the surrogate statistics, we inverted the sign of the coactivity *z* values of a random subset of the empirical coactivity maps. We then estimated the surrogate cluster statistics exactly as for the empirical data and kept the maximum surrogate cluster statistic (*t*_max-cluster-surrogate_; *n* = 2,001). For each empirical cluster statistic (*t*_cluster-empirical_), we finally tested whether it exceeded the 95th percentile of all maximum surrogate cluster statistics (*t*_max-cluster-surrogate_). If so, the empirical cluster was considered significant. We also performed a variant of this cluster-based permutation test against chance (Supplementary Fig. 15), where we subtracted a surrogate coactivity map from each corresponding empirical coactivity map before calculating the statistics across cell pairs. The surrogate coactivity map of a given cell pair was estimated by circularly shifting the ripple-locked activity levels of the object cell relative to the ripple-locked activity levels of the place cell by a random latency before calculating the two-dimensional coactivity map. The statistics across associative cell pairs were then performed as described above for the comparison against zero.

When using cluster-based permutation tests to compare the coactivity maps of associative object cell–place cell–ripple channel combinations (set A) against baseline data or against the coactivity maps of non-associative object cell–place cell–ripple channel combinations (set B), we proceeded as follows. We performed a two-sample *t*-test between the coactivity *z* values of the two sets of coactivity maps, separately for each bin of the two-dimensional time-by-time coactivity maps. We next identified clusters of contiguous bins, for which the uncorrected *P* value was significant (α = 0.05) and the *t-*statistic was positive. For each cluster, we then computed the sum of all *t* values (*t*_cluster-empirical_) and compared these empirical cluster statistics against 2,001 surrogate statistics. To obtain each of the surrogate statistics, we swapped a random subset of the set A and set B coactivity maps (when comparing the coactivity maps against baseline coactivity maps) or randomly reassigned the coactivity maps to the two sets A and B (when comparing the coactivity maps against coactivity maps of non-associative cell pairs). We then estimated cluster statistics exactly as for the empirical data and kept the maximum cluster statistic (*t*_max-cluster-surrogate_). For each empirical cluster statistic (*t*_cluster-empirical_), we finally tested whether it exceeded the 95th percentile of all maximum surrogate cluster statistics (*t*_max-cluster-surrogate_). If so, the empirical cluster was considered significant.

To further describe the data underlying the coactivity maps in Fig. 7, Supplementary Fig. 16 shows the number of object cell–place cell–ripple channel combinations contributing to the coactivity maps (which can vary between the bins in the coactivity maps); the total number of ripples underlying the coactivity maps (which can also vary between the different bins in the coactivity maps); the individual coactivity *z*-scores underlying the maxima in the coactivity maps; and the brain regions of the object cells and place cells contributing to the maxima in the coactivity maps. To validate our method of using *z*-scores to compute coactivations (Fig. 7), we also computed the coactivations using Pearson correlations (between the activity vectors of object cells and place cells) and obtained qualitatively identical results (Supplementary Fig. 18).

In our main results (Fig. 7c–h), we separately considered early and late ripples (that is, ripples 1 to *n* / 2 and ripples *n* / 2 + 1 to *n*, respectively, where *n* is the total ripple number) and computed the coactivity maps separately for the group of early ripples and the group of late ripples. To understand whether this distinction mainly followed a distinction between ripples occurring before the initial formation and ripples occurring after the initial formation of associative memories, we performed a separate analysis in which we estimated the trial in which the participant exhibited the strongest improvement in memory performance, separately for each object. We identified the object-specific trial of strongest memory improvement by (1) estimating the memory performance on each trial; (2) smoothing these performance values with a running average of three trials (to attenuate the effect of potential outliers); (3) iteratively computing a two-sample *t*-test between the memory performance values from trials (*i* + 1):*n* and those from trials 1:*i*, where *i* is the current trial index and *n* is the total number of trials; and (4) identifying the trial with the largest *t-*statistic (where the *t-*statistics of the first and last trial were not considered, to exclude the possibility that they were selected as the trial with the largest *t-*statistic). We considered all ripples occurring before or in the trial with the largest *t-*statistic as ripples occurring ‘before initial memory formation’. All other ripples (occurring after the trial with the largest *t-*statistic) were considered as ripples occurring ‘after initial memory formation’. The results for ripples occurring before initial memory formation and those occurring after initial memory formation were very similar to the results for early versus late ripples (Supplementary Fig. 22), suggesting that the significant object cell–place cell coactivations during late ripples were at least partly dependent on an initial formation of the associative memories.

To understand whether the main coactivity results (Fig. 7e,h) were driven by ripples occurring during movement or during non-movement, we categorized each timepoint of the task according to whether or not the subject was moving and analyzed the data separately for both conditions. We defined movement periods as those periods in which the participant’s movement speed was above 0.001 virtual units per second (vu s^−1^). The movement profile of two example trials is shown in Supplementary Fig. 23. All other periods were defined as non-movement (rest) periods. We observed that the cellular coactivations were mainly driven by ripples occurring during non-movement periods (Supplementary Fig. 23).

### Reporting summary

Further information on research design is available in the Nature Portfolio Reporting Summary linked to this article.

## Online content

Any methods, additional references, Nature Portfolio reporting summaries, source data, extended data, supplementary information, acknowledgements, peer review information; details of author contributions and competing interests; and statements of data and code availability are available at 10.1038/s41593-023-01550-x.

### Supplementary information

Supplementary Information Supplementary Tables 1–5, Figs. 1–24 and References.

Reporting Summary

## Data Availability

Data to recreate the figures are available at https://github.com/NeuroLuke/KunzNatureNeuroscience2024. Raw data are not publicly available because they could compromise research participant privacy, but they are available upon reasonable request from the corresponding author. Researchers requesting the data will have to sign an agreement that they will not try to de-identify the data and that they will use the data for scientific purposes only. Any additional information required to reanalyze the data reported in this paper is available from the corresponding author upon reasonable request.

## Code availability

All custom MATLAB code generated during this study for data analysis are available at https://github.com/NeuroLuke/KunzNatureNeuroscience2024.
