# Supplementary material for: Ripple-locked coactivity of stimulus-specific neurons and human associative memory
Source: Nat Neurosci. 2024 Feb 16;27(3):587–99. doi: 10.1038/s41593-023-01550-x (PMC10917673; doi:10.1038/s41593-023-01550-x)
Supplement: Supplementary file 1 — Supplementary Tables 1–5, Figs. 1–24 and References. [file 41593_2023_1550_MOESM1_ESM.pdf]

nature neuroscience

Article https://doi.org/10.1038/s41593-023-01550-x

Ripple-locked coactivity of stimulus-specific neurons and human associative memory

In the format provided by the authors and unedited

Supplementary Information: Ripple-locked coactivity of stimulus-specific neurons and human associative memory

Lukas Kunz,[1,2]* Bernhard P. Staresina,[3,4] Peter C. Reinacher,[5,6] Armin Brandt,[2] Tim A. Guth,[1,2] Andreas Schulze-Bonhage,[2,9] and Joshua Jacobs[7,8,9]

[1]Department of Epileptology, University Hospital Bonn, Bonn, Germany.

[2]Epilepsy Center, Medical Center – University of Freiburg, Faculty of Medicine, University of Freiburg, Freiburg, Germany.

[3]Department of Experimental Psychology, University of Oxford, Oxford, UK.

[4]Oxford Centre for Human Brain Activity, Wellcome Centre for Integrative Neuroimaging, Department of Psychiatry, University of Oxford, Oxford, UK.

[5]Department of Stereotactic and Functional Neurosurgery, Medical Center – University of Freiburg, Faculty of Medicine, University of Freiburg, Freiburg, Germany.

[6]Fraunhofer Institute for Laser Technology, Aachen, Germany.

[7]Department of Biomedical Engineering, Columbia University, New York, NY, USA.

[8]Department of Neurological Surgery, Columbia University Medical Center, New York, NY, USA.

[9]These authors contributed equally to this work.

*Correspondence: lukas.kunz@ukbonn.de.

Supplementary Tables

Table S1. Subject information.

Subject index	Session index	Micro-electrode data (# BF electrodes)	AMY channels (# micro-electrodes)	EC channels (# micro-electrodes)	HC channels (# micro-electrodes)	PHC channels (# micro-electrodes)	TP channels (# micro-electrodes)	# cells	# object cells	# place cells	# of trials
**FR001**	1	N (0)	L; R	-	L; R	L; R	L; R	-	-	-	52
**FR002a**	2	N (0)	R	R	R	R	R	-	-	-	130
**FR002b**	3	N (0)	R	R	R	R	R	-	-	-	152
**FR003**	4	N (0)	L; R	-	L; R	-	L; R	-	-	-	41
**FR004a**	5	N (0)	L	R	L; R	L; R	L; R	-	-	-	160
**FR004b**	6	N (0)	L	R	L; R	L; R	L; R	-	-	-	131
**FR005a**	7	N (0)	R	R	R	-	R	-	-	-	91
**FR005b**	8	N (0)	R	R	R	-	R	-	-	-	85
**FR006a**	9	Y (8)[1]	L (8); R (8)	R (8)	L; R (8)	L (8)	L (8); R (8)	71	2	4	39
**FR006b**	10	Y (8)[1]	L (8); R (8)	R (8)	L; R (8)	L (8)	L (8); R (8)	49	4	8	78
**FR007**	11	Y (4)	R (8)	R (8)	R (8)	R (8)	R	43	1	4	34
**FR008a**	12	Y (3)[2]	L (8)	-	L (8)	-	L	27	4	2	160
**FR008b**	13	Y (3)[2]	L (8)	-	L (8)	-	L	15	0	0	160
**FR009a**	14	Y (6)	L (8); R (8)	-	L (8); R (8)	L; R (8)	L; R (8)	56	5	4	160
**FR009b**	15	Y (6)	L (8); R (8)	-	L (8); R (8)	L; R (8)	L; R (8)	47	4	5	160
**FR010**	16	Y (6)	L (8); R (8)	-	L (8); R (16)	L (8)	L; R	30	0	2	54
**FR011**	17	Y (6)	L (8); R (8)	L (8)	L (8); R	L (8); R	R (8)	52	3	4	98
**FR012a**	18	N (0)	L	-	L	-	L	-	-	-	151
**FR012b**	19	N (0)	L	-	L	-	L	-	-	-	81
**FR013**	20	Y (3)	-	-	R (8)	R (8)	R (8)	12	0	0	36
**FR014**	21	Y (4)	L (8)	L (8)	L (8)	L (8)	L	53	2	3	67
**FR015b**	22	N (0)	R	-	R	R	R	-	-	-	113
**FR016**	23	N (0)	R	L; R	L; R	R	R	-	-	-	126
**FR017**	24	Y (6)	R (8)	-	L (8); R (8)	R (8)	L (8); R (8)	55	11	12	160
**FR018**	25	N (0)	-	-	L	-	-	-	-	-	84
**FR019**	26	N (0)	R	-	R	R	R	-	-	-	82
**FR020**	27	Y (4)	R (8)	R (8)	R (16)	-	R	33	2	3	102
**FR021**	28	Y (6)	L; R (8)	-	L (8); R (8)	L; R (8)	L (8); R (8)	42	3	3	54
**FR022**	29	Y (6)	L (8); R (8)	-	L (16); R (8)	-	L (8); R	50	5	2	102

**FR023a**	30	Y (6)	L (8); R (8)	L (8); R	L (8); R (8)	L (8)	L	48	6	7	160
**FR023b**	31	Y (6)	L (8); R (8)	L (8); R	L (8); R (8)	L (8)	L	42	0	3	160
**FR024**	32	Y (6)[3]	-	R (8)	R (8)	R (8)	R (8)	56	19	10	94
**FR025a**	33	Y (2)	L; R (8)	R (8)	L; R	L	L; R	18	9	2	111
**FR025b**	34	Y (2)	L; R (8)	R (8)	L; R	L	L; R	17	5	1	106
**FR025c**	35	Y (2)	L; R (8)	R (8)	L; R	L	L; R	10	3	2	108
**FR026**	36	Y (6)	L (8); R (8)	R (8)	L (8); R (8)	R (8)	L; R	44	5	9	80
**FR027**	37	Y (4)	R (8)	R (8)	R (8)	R (8)	R	5	0	1	117
**FR028a**	38	Y (4)	L (8)	L (8)	L (8)	L (8)	L	46	6	2	111
**FR028b**	39	Y (4)	L (8)	L (8)	L (8)	L (8)	L	38	5	2	33
**FR029**	40	Y (6)	L (8); R (8)	R (8)	L (8); R (8)	R (8)	L; R	62	12	7	112
**FR030**	41	Y (4)	R (8)	R (8)	R (8)	R (8)	R	42	4	7	72

The column "Microelectrode data" states whether microelectrode recordings using Behnke-Fried depth electrodes were performed in a given subject and how many Behnke-Fried depth electrodes were implanted in that subject (in parentheses). The columns "AMY channels", "EC channels", "HC channels", "PHC channels", and "TP channels" state whether depth electrodes and how many microelectrodes (in parentheses) were implanted in that particular region in a given subject. AMY, amygdala; EC, entorhinal cortex; HC, hippocampus; PHC, parahippocampal cortex; TP, temporal pole. #, number; BF electrode, Behnke-Fried depth electrode that includes 8 microelectrodes for single-neuron recordings (plus one reference electrode); L, left; R, right. N, no; Y, yes. Subject indices with letters "b" or "c" indicate second and third sessions performed by this subject, respectively. [1], microelectrodes were also implanted in the left insula; [2], microelectrodes were also implanted in the left fusiform gyrus; [3], microelectrodes were also implanted in the right fusiform gyrus and the right visual cortex.

Table S2. Detailed statistical information for analyses in the main text.

Test	Descriptive and inferential statistics
Fig. 1. Hypothesis and associative object–location memory task	
Average duration of retrieval periods (Fig. 1b)	13.415 ± 0.218 s (mean ± SEM), *n* = 4184 retrieval periods.
Average duration of the re-encoding periods (Fig. 1b)	7.685 ± 0.158 s (mean ± SEM), *n* = 4175 re-encoding periods.
Section: Human hippocampal ripples and object–location memory	
Total number of subjects	*n* = 30.
Number of subjects with single-neuron recordings	*n* = 20.
Total number of sessions	*n* = 41.
Number of sessions with single-neuron recordings	*n* = 27.
Number of trials per session	103 ± 6 (mean ± SEM); range, 33–160.
Duration per session (minutes)	49 ± 3 (mean ± SEM); range, 22–150.
Two-sided paired *t*-test between memory performance during early versus late trials (Fig. 1d, left)	$t(40) = -4.788$, $P < 0.001$ when considering all sessions; $t(26) = -3.926$, $P = 0.001$ when only considering sessions with single-neuron recordings.
Two-sided Pearson correlation between normalized time and average memory performance (Fig. 1d, right)	$r = 0.872$, $P < 0.001$, *n* = 20 time bins.
Total number of hippocampal ripples	*n* = 35948.
Total number of hippocampal bipolar channels	*n* = 62.
Number of hippocampal bipolar channels during sessions with single-neuron recordings	*n* = 43.
Phase locking of hippocampal ripples to hippocampal delta phases (Fig. 2i)	Mean delta phase, 34 ± 68° (circular mean ± circular SD); Rayleigh test: $z = 5.614$, $P = 0.003$; comparison of the empirical Rayleigh *z*-value against surrogate Rayleigh *z*-values based on surrogate ripples with shuffled inter-ripple intervals: $P = 0.017$; *n* = 62 channels; two-sample Kuiper's test to examine whether empirical delta phases were different from surrogate delta phases: $k = 1132988.000$, $P = 0.001$.
Fig. 3. Ripples in the human hippocampus are linked to behavioral state and memory performance in an associative object–location memory task	
ANOVA to examine the relationship between ripple rates and trial phase (Fig. 3a)	$F(4, 244) = 19.942$, $P < 0.001$.
Ripple rates during the different trials phases in Hz (mean ± SEM)	ITI: 0.143 ± 0.010; cue: 0.157 ± 0.011; retrieval: 0.123 ± 0.008; feedback: 0.106 ± 0.007; re-encoding: 0.121 ± 0.008.
ANOVA to examine the relationship between ripple durations and trial phase (Fig. 3a)	$F(4, 236) = 2.463$, $P = 0.046$.
ANOVA to examine the relationship between ripple frequencies and trial phase (Fig. 3a)	$F(4, 236) = 0.562$, $P = 0.690$.
Two-sided one-sample *t*-tests on the channel-wise Pearson correlations between ripple rate and memory performance per trial phase	ITI: $t(60) = -0.469$, $P = 1$; cue: $t(60) = 2.763$, $P = 0.038$; retrieval: $t(60) = -0.495$, $P = 1$; feedback: $t(59) = 1.807$, $P = 0.380$; re-

	encoding: $t(60) = -4.181$, $P < 0.001$. P-values are Bonferroni corrected for five tests.
Two-sided one-sample t-tests on the channel-wise Pearson correlations between ripple duration and memory performance per trial phase	ITI: $t(60) = -1.434$, $P = 0.784$; cue: $t(59) = 1.224$, $P = 1$; retrieval: $t(60) = 1.292$, $P = 1$; feedback: $t(54) = 2.491$, $P = 0.079$; re-encoding: $t(60) = -1.321$, $P = 0.958$. P-values are Bonferroni corrected for five tests.
Two-sided cluster-based permutation tests to investigate whether time-resolved ripple rates differed between good- and bad-memory performance trials	ITI: no significant clusters; cue: $t_{cluster} = 1023.281$, $P = 0.011$; retrieval: no significant clusters; feedback: no significant clusters; re-encoding: no significant clusters.
Two-sided one-sample t-tests on the channel-wise Pearson correlations between ripple rate and trial index per trial phase	ITI: $t(60) = 0.208$, $P = 1$; cue: $t(60) = -1.947$, $P = 0.281$; retrieval: $t(60) = 0.612$, $P = 1$; feedback: $t(59) = -3.178$, $P = 0.012$; re-encoding: $t(60) = 2.193$, $P = 0.161$. P-values are Bonferroni corrected for five tests.
Section: Neural signature of hippocampal ripples across the human MTL	
One-sided cluster-based permutation test on the z-scored cross-correlation values against 0 to show that ripple events in extrahippocampal MTL regions were coupled to hippocampal ripples (Fig. 4a)	$t_{cluster} = 1897.513$, $P < 0.001$, $n = 296$ extrahippocampal–intrahippocampal channel pairs.
Two-sided cluster-based permutation test on normalized LFP power against 0 across all extrahippocampal MTL channels ipsilateral to hippocampal ripple channels, positive direction (Fig. 4b, left)	$t_{cluster} = 22317.700$, $P = 0.018$, $n = 178$ ipsilateral channels.
Two-sided cluster-based permutation test on normalized LFP power against 0 across all extrahippocampal MTL channels contralateral to hippocampal ripple channels, positive direction (Fig. 4b, right)	$t_{cluster} = 70525.690$, $P < 0.001$, $n = 118$ contralateral channels.
Two-sided cluster-based permutation test on normalized LFP power against 0 across all extrahippocampal MTL channels ipsilateral to hippocampal ripple channels, negative direction (Fig. 4b, left)	$t_{cluster} = -151182.988$, $P < 0.001$, $n = 178$ ipsilateral channels.
Two-sided cluster-based permutation test on normalized LFP power against 0 across all extrahippocampal MTL channels contralateral to hippocampal ripple channels, negative direction (Fig. 4b, right)	$t_{cluster} = -196032.061$, $P < 0.001$, $n = 118$ contralateral channels.
Total number of neurons	$n = 1063$.
Number of neurons in different brain regions	Amygdala: $n = 340$; entorhinal cortex: $n = 214$; fusiform gyrus: $n = 24$; hippocampus: $n = 213$; insula: $n = 2$; parahippocampal cortex: $n = 126$; temporal pole: $n = 135$; visual cortex: $n = 9$.
Number of neuron–ripple-channel combinations	$n = 1716$.
One-sided cluster-based permutation test on z-scored single-neuron firing rates during hippocampal ripples against 0 (Fig. 4d)	$t_{cluster} = 4202.806$, $P < 0.001$, $n = 1716$ neuron–ripple-channel combinations.

Section: Neurons in the human MTL are tuned to objects and locations	
Number and prevalence of object cells	120; 11.289% of all cells.
Two-sided binomial test of object-cell prevalence against 5% chance	$P < 0.001$.
Two-sided Pearson correlation between the preferred-object tuning curve from the first data half and the preferred-object tuning curve from the second data half to demonstrate temporal stability of object-cell tuning (Fig. 5e)	Pearson's $r = 0.368 \pm 0.035$ (mean ± SEM); one-sample two-sided *t*-test of correlation values against 0: $t(118) = 10.387$, $P < 0.001$.
Number of pure object cells (Fig. 5f)	97; 80.833% of all object cells.
Number and prevalence of place cells	109; 10.254%.
Two-sided binomial test of place-cell prevalence against 5% chance	$P < 0.001$.
Number of place cells whose place fields have some contact to the edge of the corresponding firing-rate maps.	104 (of 109).
Fraction of spatial bins of the place fields of place cells that are next to the edge of the corresponding firing-rate maps (Fig. 6e)	18.327 ± 1.266% (mean ± SEM).
Number of objects inside place fields (Fig. 6f)	1.505 ± 0.089 (mean ± SEM).
Two-sided two-sample Kolmogorov-Smirnov test to examine whether the number of objects per place field is increased as compared to surrogate place fields (Fig. 6f)	$D = 0.053$, $P = 0.915$.
Two-sided paired *t*-test to compare firing rates inside place fields with firing rates outside place fields (Fig. 6g)	$t(108) = 12.350$, $P < 0.001$.
Two-sided Pearson correlation between the firing-rate map from the first data half and the firing-rate map from the second data half to demonstrate temporal stability of place-cell tuning (Fig. 6h)	$r = 0.147 \pm 0.021$ (mean ± SEM); two-sided one-sample *t*-test of correlation values against 0: $t(108) = 7.080$, $P < 0.001$.
Section: Ripple-locked cellular coactivity and object–location memory	
Total number of combinations between object cells, place cells, and ripple channels	$n = 1104$.
One-sided cluster-based permutation test on the coactivity maps of associative cell pairs versus 0, retrieval (Fig. 7c, left)	$t_{cluster} = 1806.271$, $P < 0.001$.
One-sided cluster-based permutation test on the coactivity maps of associative cell pairs versus baseline coactivity maps, retrieval (Fig. 7c, middle)	$t_{cluster} = 390.563$, $P = 0.178$.
One-sided cluster-based permutation test on the coactivity maps of associative cell pairs versus the coactivity maps of non-associative cell pairs, retrieval (Fig. 7c, right)	$t_{cluster} = 1753.851$, $P < 0.001$.
One-sided cluster-based permutation test on the coactivity maps of associative cell pairs versus 0, retrieval, early ripples (Fig. 7d, left)	$t_{cluster} = 911.469$, $P = 0.038$ (Bonferroni corrected for performing this analysis on both early and late ripples).
One-sided cluster-based permutation test on the coactivity maps of associative cell pairs versus	$t_{cluster} = 202.215$, $P = 0.884$ (Bonferroni corrected for performing this analysis on both early and late ripples).

baseline coactivity maps, retrieval, early ripples (Fig. 7d, middle)	
One-sided cluster-based permutation test on the coactivity maps of associative cell pairs versus the coactivity maps of non-associative cell pairs, retrieval, early ripples (Fig. 7d, right)	$t_{cluster}$ = 1170.103, $P = 0.025$ (Bonferroni corrected for performing this analysis on both early and late ripples).
One-sided cluster-based permutation test on the coactivity maps of associative cell pairs versus 0, retrieval, late ripples (Fig. 7e, left)	$t_{cluster}$ = 1688.581, $P = 0.004$ (Bonferroni corrected for performing this analysis on both early and late ripples).
One-sided cluster-based permutation test on the coactivity maps of associative cell pairs versus baseline coactivity maps, retrieval, late ripples (Fig. 7e, middle)	$t_{cluster}$ = 1378.822, $P = 0.004$ (Bonferroni corrected for performing this analysis on both early and late ripples).
One-sided cluster-based permutation test on the coactivity maps of associative cell pairs versus the coactivity maps of non-associative cell pairs, retrieval, late ripples (Fig. 7e, right)	$t_{cluster}$ = 1571.685, $P = 0.006$ (Bonferroni corrected for performing this analysis on both early and late ripples).
One-sided cluster-based permutation test on the coactivity maps of associative cell pairs versus 0, re-encoding (Fig. 7f, left)	$t_{cluster}$ = 3202.072, $P < 0.001$.
One-sided cluster-based permutation test on the coactivity maps of associative cell pairs versus baseline coactivity maps, re-encoding (Fig. 7f, middle)	$t_{cluster}$ = 622.631, $P = 0.064$.
One-sided cluster-based permutation test on the coactivity maps of associative cell pairs versus the coactivity maps of non-associative cell pairs, re-encoding (Fig. 7f, right)	$t_{cluster}$ = 2903.404, $P < 0.001$.
One-sided cluster-based permutation test on the coactivity maps of associative cell pairs versus 0, re-encoding, early ripples (Fig. 7g, left)	$t_{cluster}$ = 304.802, $P = 0.599$ (Bonferroni corrected for performing this analysis on both early and late ripples).
One-sided cluster-based permutation test on the coactivity maps of associative cell pairs versus baseline coactivity maps, re-encoding, early ripples (Fig. 7g, middle)	$t_{cluster}$ = 113.665, $P = 1$ (Bonferroni corrected for performing this analysis on both early and late ripples).
One-sided cluster-based permutation test on the coactivity maps of associative cell pairs versus the coactivity maps of non-associative cell pairs, re-encoding, early ripples (Fig. 7g, right)	$t_{cluster}$ = 504.950, $P = 0.235$ (Bonferroni corrected for performing this analysis on both early and late ripples).
One-sided cluster-based permutation test on the coactivity maps of associative cell pairs versus 0, re-encoding, late ripples (Fig. 7h, left)	$t_{cluster}$ = 2670.873, $P < 0.001$ (Bonferroni corrected for performing this analysis on both early and late ripples).
One-sided cluster-based permutation test on the coactivity maps of associative cell pairs versus baseline coactivity maps, re-encoding, late ripples (Fig. 7h, middle)	$t_{cluster}$ = 947.243, $P = 0.040$ (Bonferroni corrected for performing this analysis on both early and late ripples).
One-sided cluster-based permutation test on the coactivity maps of associative cell pairs versus the coactivity maps of non-associative cell pairs, re-encoding, late ripples (Fig. 7h, right)	$t_{cluster}$ = 2231.542, $P < 0.001$ (Bonferroni corrected for performing this analysis on both early and late ripples).

Table S3. Linear mixed model to analyze the prevalence of interictal epileptic discharges (IEDs; dependent variable) as a function of behavior.

Predictor	*t*	DF	*P*
Memory performance	-0.031	32133	0.975
Trial index	2.398	32133	0.017*
Cue	-5.247	32133	<0.001***
Retrieval	1.781	32133	0.075
Feedback	-2.134	32133	0.033*
Re-encoding	-1.787	32133	0.074
Memory performance : trial index	1.458	32133	0.145
Memory performance : cue	-0.004	32133	0.996
Memory performance : retrieval	0.407	32133	0.684
Memory performance : feedback	1.329	32133	0.184
Memory performance : re-encoding	1.628	32133	0.103
Trial index : cue	0.093	32133	0.926
Trial index : retrieval	-1.802	32133	0.071
Trial index : feedback	0.826	32133	0.409
Trial index : re-encoding	-0.837	32133	0.403
Memory performance : trial index : cue	-0.711	32133	0.477
Memory performance : trial index : retrieval	-0.285	32133	0.776
Memory performance : trial index : feedback	-1.029	32133	0.304
Memory performance : trial index : re-encoding	-0.690	32133	0.490

*$P < 0.05$; ***$P < 0.001$. DF, degrees of freedom; :, interaction.

Model formula: IED prevalence ~ 1 + memory performance * trial index + memory performance * trial phase + trial index * trial phase + memory performance : trial index : trial phase + (1 | channel index).

Table S4. Linear mixed model to analyze ripple rates (dependent variable) as a function of behavior.

**Predictor**	***t***	**DF**	***P***
Memory performance	-0.106	32133	0.915
Trial index	0.343	32133	0.732
Cue	3.351	32133	<0.001***
Retrieval	-4.815	32133	<0.001***
Feedback	-9.416	32133	<0.001***
Re-encoding	-5.808	32133	<0.001***
Memory performance : trial index	1.351	32133	0.177
Memory performance : cue	2.599	32133	0.009**
Memory performance : retrieval	0.250	32133	0.803
Memory performance : feedback	0.583	32133	0.560
Memory performance : re-encoding	-2.754	32133	0.006**
Trial index : cue	-3.483	32133	<0.001***
Trial index : retrieval	-0.801	32133	0.423
Trial index : feedback	-2.824	32133	0.005**
Trial index : re-encoding	1.259	32133	0.208
Memory performance : trial index : cue	-1.327	32133	0.185
Memory performance : trial index : retrieval	-1.672	32133	0.094
Memory performance : trial index : feedback	-1.333	32133	0.183
Memory performance : trial index : re-encoding	-0.173	32133	0.862

**$P < 0.01$; ***$P < 0.001$. DF, degrees of freedom; :, interaction.

Model formula: ripple rate ~ 1 + memory performance * trial index + memory performance * trial phase + trial index * trial phase + memory performance : trial index : trial phase + (1 | channel index).

Table S5. Linear mixed model to analyze ripple rates (dependent variable) as a function of behavior and artifacts.

Predictor	*t*	DF	*P*
Artifact prevalence	-22.317	32132	<0.001***
Memory performance	-0.119	32132	0.905
Trial index	0.628	32132	0.530
Cue	2.685	32132	0.007**
Retrieval	-4.696	32132	<0.001***
Feedback	-9.772	32132	<0.001***
Re-encoding	-6.166	32132	<0.001***
Memory performance : trial index	1.564	32132	0.118
Memory performance : cue	2.613	32132	0.009**
Memory performance : retrieval	0.308	32132	0.758
Memory performance : feedback	0.775	32132	0.438
Memory performance : re-encoding	-2.568	32132	0.010*
Trial index : cue	-3.495	32132	<0.001***
Trial index : retrieval	-1.021	32132	0.307
Trial index : feedback	-2.747	32132	0.006**
Trial index : re-encoding	1.149	32132	0.251
Memory performance : trial index : cue	-1.438	32132	0.150
Memory performance : trial index : retrieval	-1.722	32132	0.085
Memory performance : trial index : feedback	-1.464	32132	0.143
Memory performance : trial index : re-encoding	-0.268	32132	0.788

*$P < 0.05$; **$P < 0.01$; ***$P < 0.001$. DF, degrees of freedom; :, interaction.

Model formula: ripple rate ~ 1 + artifact prevalence + memory performance * trial index + memory performance * trial phase + trial index * trial phase + memory performance : trial index : trial phase + (1 | channel index).

Supplementary Figures

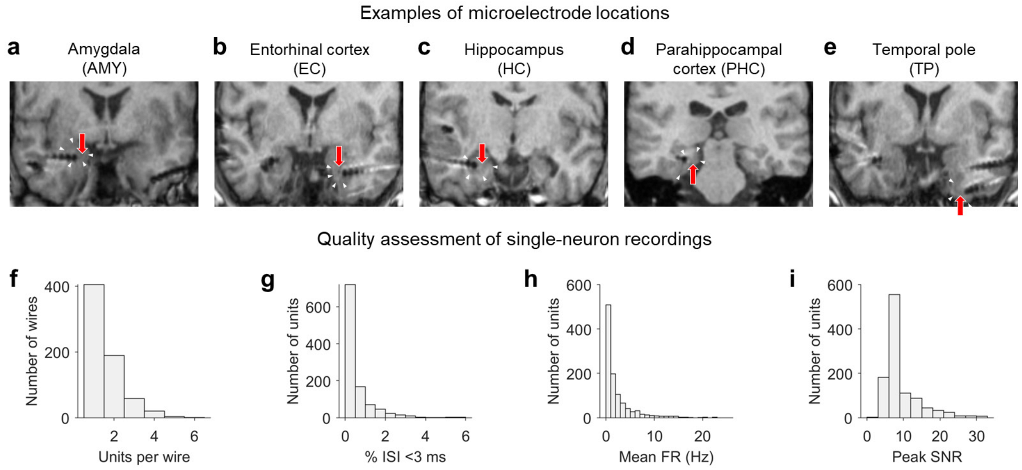

Fig. S1. Single-neuron recordings: anatomical locations and quality assessment. a–e, Example microelectrodes in amygdala (AMY), entorhinal cortex (EC), hippocampus (HC), parahippocampal cortex (PHC), and temporal pole (TP). Electrode contacts of macroelectrodes appear as dark circles on the MRI scans. Red arrows point at putative microelectrode locations, which protrude 3–5 mm from the tip of the depth electrode (often not visible on MRI scans). White triangles indicate the borders of the different brain regions. **f**, Histogram of units per wire. On average, 1.570 ± 0.032 (mean ± SEM) units per wire were recorded (only considering wires with at least one unit). **g**, Histogram of the percentages of inter-spike intervals (ISIs) that were shorter than 3 ms. On average, units exhibited 0.507 ± 0.024% (mean ± SEM) ISIs that were shorter than 3 ms. **h**, Histogram of mean firing rates (FRs). On average, units exhibited mean FRs of 2.399 ± 0.108 (mean ± SEM) Hz, which is comparable to previous human single-neuron studies (e.g., ref. [1]). **i**, Histogram of the mean waveform peak signal-to-noise ratio (SNR) of each unit. On average, the SNR of the mean waveform peak was 9.181 ± 0.160 (mean ± SEM).

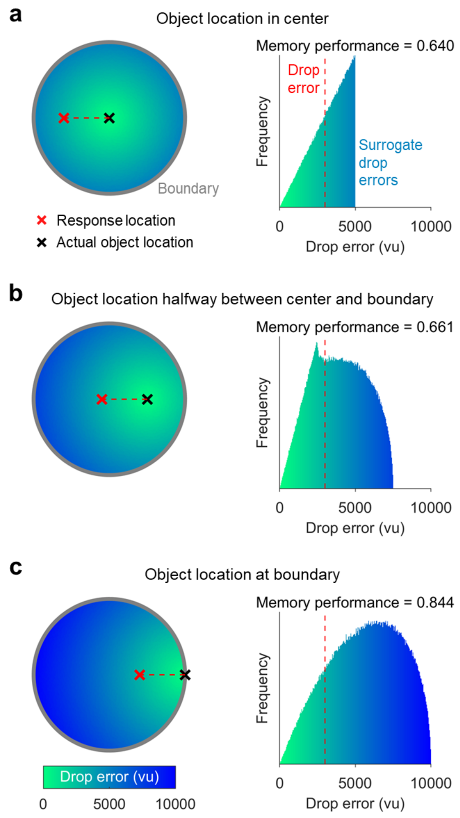

Fig. S2. Illustration of converting drop errors into memory performance values. a, The drop error (dashed red line) is the Euclidean distance between the response location (red X) and the correct object location (black X). The largest possible drop error differs depending on the proximity of the correct object location to the environment center. To compute a normalized memory performance value that accounts for the distribution of possible responses for each object, 10^7 locations were randomly drawn from inside the arena to compute surrogate drop errors. This memory performance value was obtained by computing how often the empirical drop error was smaller than the surrogate drop errors, divided by the number of surrogate drop errors (thus ranging between 0 and 1). A memory performance value of 1 is the best possible response; a memory performance value of 0 is the worst possible response. **b**, Same as in **a** for an object that is located halfway between the center and the boundary of the arena. **c**, Same as in **a** but for an object that has its correct location at the boundary of the arena. Across all panels, these illustrations show that the same absolute drop error of 3,000 virtual units (vu) leads to a memory performance of 0.64 if the object is located in the center; a memory performance of 0.661 if it is located halfway between the center and the boundary; and to a memory performance of 0.844 if the object is located at the boundary. Note the histograms of surrogate drop errors on the right-hand side, showing that the distributions of surrogate drop errors differ depending on the correct object location.

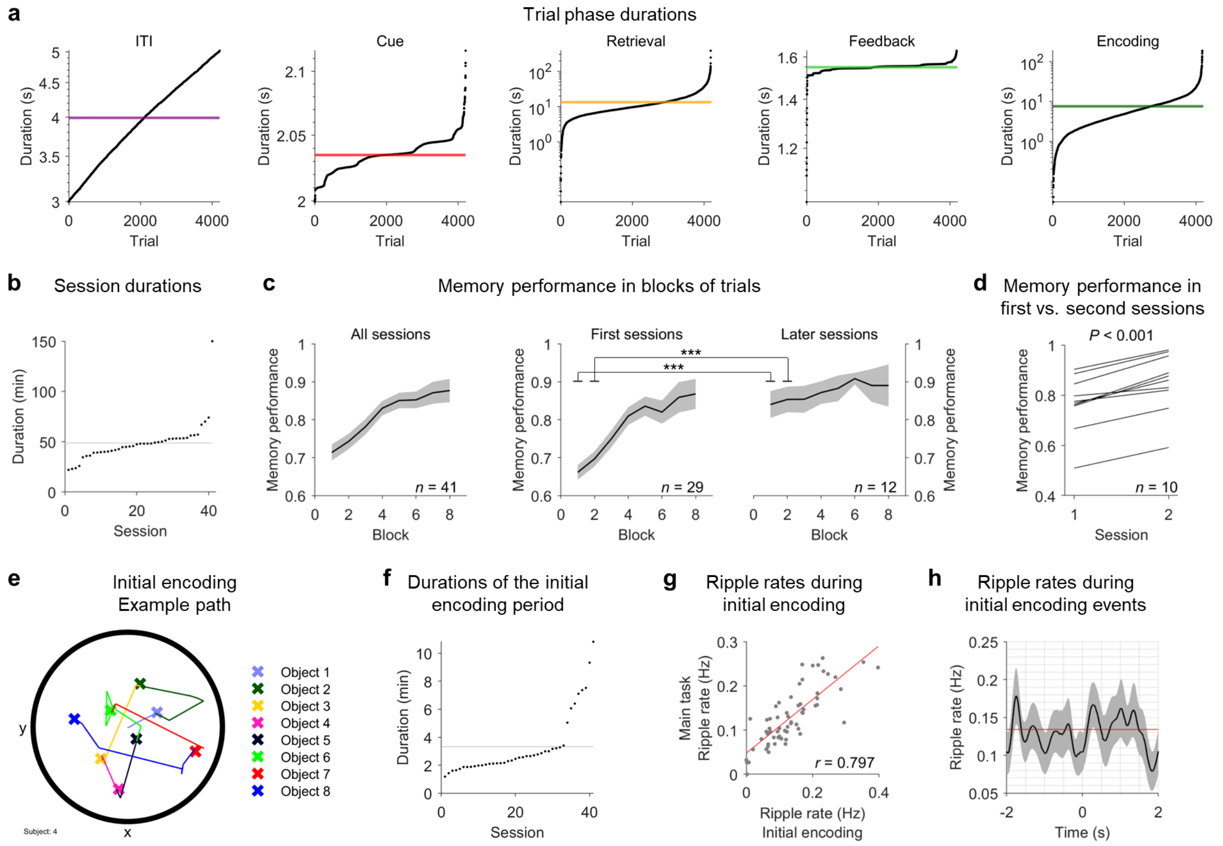

Fig. S3. Additional behavioral results. a, Duration of each trial phase, pooled across all trials from all sessions. Each dot is the duration of one trial phase. Y-axis is on a log scale. **b**, Duration of each session, sorted in ascending order. Each dot is one session. **c**, Left: Memory performance per block of trials, where each block comprises 20 trials. Black line, mean across sessions; gray shading, mean ± SEM. Middle and right: Memory performance per block of trials, separately for first (middle) and later sessions (right). For the first and second block of trials, memory performance was higher in later sessions than in first sessions [two-sided two-sample *t*-test for the first block: $t(39) = -4.743$, $P < 0.001$; for the second block: $t(39) = -4.179$, $P = 0.001$; *P*-values are Bonferroni corrected for eight comparisons]. **d**, Memory performance was overall higher in second sessions as compared to first sessions [two-sided paired *t*-test: $t(9) = -9.007$, $P < 0.001$]. Each line connects the memory performance from the first and second session of a given subject. **e**, An example subject's navigation path during the initial encoding period. Each X indicates the location of one of the eight objects and the subject's navigation path toward that object is shown as a line with the same color. **f**, Duration of each initial encoding period, sorted in ascending order. Each dot is one initial encoding period. Gray line, mean. **g**, Correlation between ripple rates during the initial encoding period versus the main task (Pearson's $r = 0.797$, $P < 0.001$). Ripple rates were not significantly different between the initial encoding period and the main task (two-sided paired *t*-test: $t(61) = 0.760$, $P = 0.450$). **h**, Time-resolved ripple rates locked to the time points (time 0) when subjects collected the objects from their correct locations during the initial encoding period (8 events per initial encoding period), averaged across all channels. This shows no change in ripple rates around these events, similar to the observation of no clear ripple-rate changes at the end of the re-encoding periods (Fig. 3). Black line, mean across sessions ($n = 41$); gray shading, mean ± SEM across sessions; red line, average ripple rate during the initial encoding period.

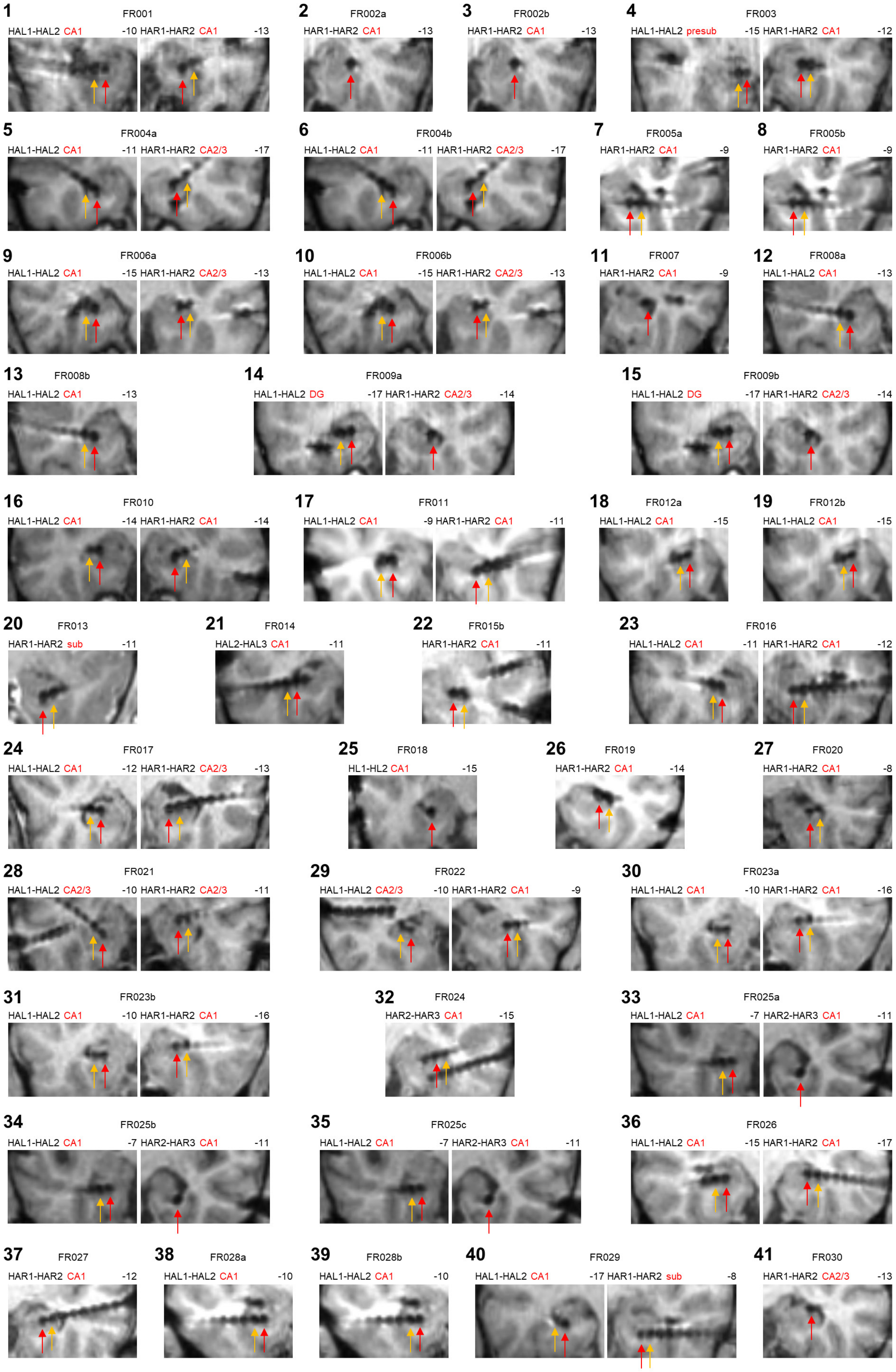

Fig. S4. Depiction of the hippocampal macroelectrode channels from all 41 sessions. The locations of the hippocampal electrodes are presented on coronal slices of the post-operative MRI scans. Red arrow, first electrode channel contributing to the

bipolar channel; yellow arrow, second electrode channel contributing to the bipolar channel. Black bold large numbers indicate the session numbers. The numbers to the right and above the images indicate the y-coordinate of the coronal slice in MNI space. Red labels, potential hippocampal subregions based on visual inspection following ref. [2]. CA1, cornu ammonis region 1; CA2/3, cornu ammonis region 2 or 3; DG, dentate gyrus; presub, presubiculum; sub, subiculum. HAL, hippocampus anterior left; HAR, hippocampus anterior right; HL, hippocampus left.

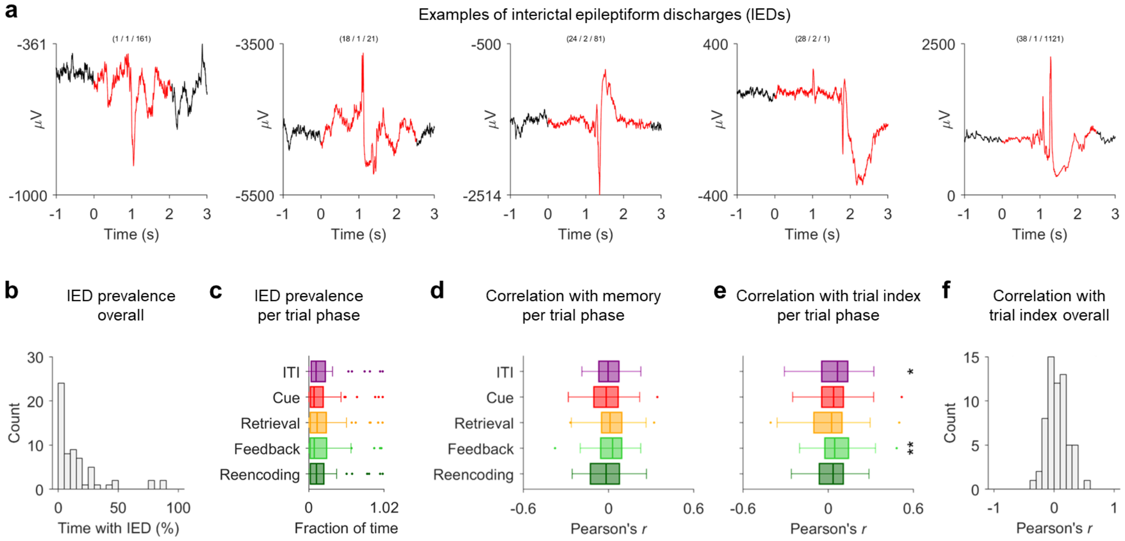

Fig. S5. Interictal epileptiform discharges (IEDs). a, Between epileptic seizures, epilepsy patients exhibit interictal epileptiform discharges (IEDs), which are pathological bursts of neuronal activity. IEDs are readily visible in intracranial EEG recordings based on their high amplitudes, sharp amplitude changes, and power increases across a broad frequency range[3,4]. IEDs complicate the detection of ripples in human epilepsy patients. Following previously established procedures for the automated detection of IEDs[3,5,6], we therefore identified IEDs in our dataset. The figure shows examples of IEDs from five different sessions. Around each IED, an additional time period of ±1 s was excluded[3]. Black, local field potential (not filtered); red, local field potential with an IED and thus excluded from subsequent ripple detection. **b**, Histogram showing the prevalence of IEDs in each session. On average, 16.591 ± 2.692% (mean ± SEM; n = 62 channels) of the data was designated as belonging to an IED. We additionally excluded a small amount of the data due to ripple-like phenomena in the grand-average signal, which are presumably muscle or other artifacts, leading to 17.380 ± 2.668% (mean ± SEM; n = 62 channels) of the data being excluded before ripple detection. As expected, we observed that a higher prevalence of IEDs was correlated with lower ripple rates across channels, both when computing ripple rates relative to the entire data and relative to data periods without artifacts (Spearman's *rho* = -0.740, $P < 0.001$ and Spearman's *rho* = -0.452, $P < 0.001$, respectively; n = 62 channels), potentially indicating that the detected ripples were indeed physiologic and that IEDs lead to a reduction of such physiological ripples[7]. **c**, Previous studies showed that IEDs lead to transitory cognitive impairments and that they impede the encoding and retrieval of associative memories[7,8]. We therefore analyzed the relationship between IEDs and behavior in our associative object–location memory task. The figure shows the prevalence of IEDs in different trial phases (n = 62 channels). A repeated measures ANOVA showed that the prevalence of IEDs in a given trial was modulated by trial phase [repeated measures ANOVA: $F(4, 244) = 3.705$, $P = 0.006$]. Pair-wise, two-sided post-hoc comparisons with correction for multiple comparisons showed that the prevalence of IEDs was significantly reduced during the cue period as compared to the ITI period ($P_{\text{Tukey-Kramer}} = 0.012$) and the retrieval period ($P_{\text{Tukey-Kramer}} < 0.001$). **d**, Correlation between the prevalence of IEDs and memory performance, separately for each trial phase (n = 62 channels). Correlations were computed separately for each channel and then averaged across channels. Correlation coefficients were not significantly different from 0 (one-sample t-tests across channel-wise correlation coefficients: all $t < 0.900$, all $P = 1$, Bonferroni corrected for five tests). We thus did not observe significant relationships between IED prevalence and memory performance, which replicates a previous report with a similar paradigm and which may be due to the self-paced nature of this task[9]. **e**, We found instead that IED prevalence slightly increased with time, which was most clearly visible for the ITI period and the feedback period. The figure shows the correlation between the prevalence of IEDs and trial index, separately for each trial phase (n = 62 channels). Correlations were computed separately for each channel and then averaged across channels. Correlation coefficients were significantly above 0 for ITI [one-sample t-test across channel-wise correlation coefficients: $t(60) = 2.985$, $P = 0.021$, Bonferroni corrected for five tests] and feedback [$t(57) = 3.339$, $P = 0.007$, Bonferroni corrected for five tests]. **f**, The positive relationship between trial index and IED prevalence was also visible irrespective of trial phase. The figure shows the correlation between the prevalence of IEDs and trial index, irrespective of trial phase. Correlation coefficients were significantly above 0, indicating that the prevalence of IEDs increased over the course of a session [one-sample t-test of channel-wise correlation coefficients between trial index and IED prevalence versus 0: $t(61) = 2.207$, $P = 0.031$]. We also used a linear mixed model to investigate the relationship between IEDs and behavior. This linear mixed model used IED prevalence in a given trial phase as dependent variable and trial-wise memory performance, trial index, and trial phase as fixed effects (Table S3). Channel index was included as a random effect. In line with the above-mentioned results, the linear mixed model showed that memory performance was not related to IED prevalence and that trial index was positively correlated with IED prevalence. As compared to the ITI period, the cue period and the feedback period showed a lower prevalence of IEDs. Together, these results indicate that IEDs are modulated by our associative object–location memory task, but that they are not directly related to memory performance in this task. Box plots show: center line, median; box limits, upper and lower quartiles; whiskers, minimum and maximum; points, outliers. *$P_{\text{corr.}} < 0.05$; **$P_{\text{corr.}} < 0.01$.

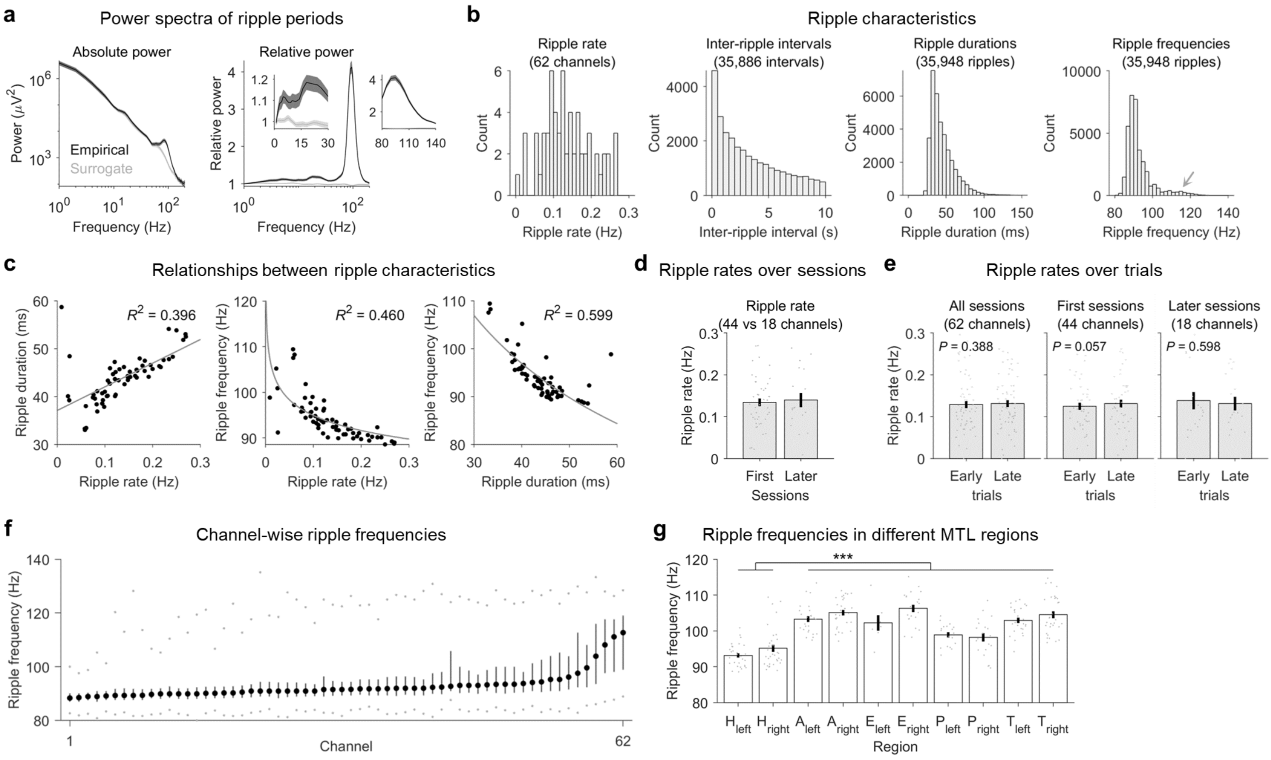

Fig. S6. Characteristics of hippocampal ripples. **a**, Raw and relative power spectrum of hippocampal ripples and hippocampal surrogate ripples (x-axis ranges from 1 to 200 Hz). Relative power was estimated by dividing the power values during the ripples by the mean power values averaged across the entire experiment (separately for each frequency). As expected, raw and relative power spectra of ripple periods showed marked peaks at about 93 Hz. Note the left small inset showing increased low gamma power (around 20 to 30 Hz) during ripple events, similar to previous results in rodents that showed that this increased gamma power reflects the power envelope of overlapping ripples[10]. We also note that we recorded ripples solely from the anterior human hippocampus because this was the part of the hippocampus with the most frequent electrode coverage, given that the patients' implantation schemes were determined by clinical needs. The human anterior hippocampus corresponds to the rodent ventral hippocampus, which poses a challenge when trying to directly compare our results to studies on hippocampal sharp-wave ripples in rodents, which typically record sharp-wave ripples from the dorsal hippocampus. Prior rodent studies showed that ripples in the ventral segment of the hippocampus are often isolated from ripples in the intermediate and dorsal segments of the hippocampus[11,12]. Accordingly, ripples in different parts of the hippocampus may play different functional roles and may process different types of information. Indeed, it has been shown that sharp-wave ripples in the dorsal hippocampus of the rat are strongly enhanced by novel and rewarding experiences, whereas ventral hippocampal sharp-wave ripples are not modulated by novelty and reward[11]. Concordantly, ripples in the dorsal and ventral hippocampus activate distinct and opposing patterns of spiking activity in the nucleus accumbens, whereby only those neurons of the nucleus accumbens are tuned to task- and reward-related information whose activity is coupled to ripples in the dorsal hippocampus[11]. These results point at differences between sharp-wave ripples in the dorsal versus ventral rodent hippocampus. Building on these findings in rodents, future studies in humans should thus investigate whether ripples in the anterior versus posterior human hippocampus have similar or different physiological and functional properties. **b**, Different ripple characteristics including ripple rates, inter-ripple intervals, ripple durations, and ripple frequencies. The identified ripples occurred at a rate of 0.136 ± 0.008 ripples/s (mean ± SEM; n = 62 channels; 0.159 ± 0.007 ripples/s when only considering artifact-free time periods) and exhibited an average inter-ripple interval of 7.382 ± 0.084 s (mean ± SEM; n = 35886 inter-ripple intervals). The ripples had an average duration of 45.210 ± 0.086 ms (mean ± SEM; n = 35948 ripples) and an average frequency of 92.603 ± 0.036 Hz (mean ± SEM; n = 35948 ripples). These ripple characteristics are comparable to previous studies using similar ripple-detection algorithms (e.g., refs. [3,5,6]). Note that there may be a small group of ripples with slightly higher frequencies at ~115 Hz (gray arrow), but the vast majority occurred at a frequency between 80–100 Hz. **c**, Pairwise relations between channel-wise ripple rates, ripple durations, and ripple frequencies. **d**, Ripple rates during first sessions were not different from ripple rates during later sessions [two-sample t-test: $t(60) = -0.307$, $P = 0.760$, n = 44 channels in first sessions, n = 18 channels in second or third sessions]. Gray dots, data from single channels. **e**, Left: ripple rates during early trials of a session were not different from ripple rates during late trials of a session [two-sided paired t-test: $t(61) = -0.869$, $P = 0.388$, n = 62 channels]. Middle, ripple rates during early trials of first sessions tended to be lower than during late trials of first sessions [two-sided paired t-test: $t(43) = -2.264$, $P = 0.057$, Bonferroni corrected for performing this analysis twice for both first and later sessions, n = 44 channels]. Right: ripple rates during early trials of later sessions were not different from ripple rates during late trials of later sessions [two-sided paired t-test: $t(17) = 1.071$, $P = 0.598$, Bonferroni corrected for performing this analysis twice for both first and later sessions, n = 18 channels]. **f**, Ripple frequency per hippocampal channel (n = 62). Large black dots, median; black vertical lines, inter-quartile range; gray dots, maximum and minimum. **g**, Ripple frequency per channel in different medial temporal lobe regions. Ripple frequencies were significantly lower

in the hippocampus than in the other regions [two-sided unpaired t-test: $t(238) = -11.947$, $P < 0.001$, $n = 240$ channels]. For a comparison of ripple frequencies between the hippocampus and neocortex, see ref. [13]. We note that it is of ongoing debate whether the periods of elevated power around 80–140 Hz in this and other human studies (e.g., refs. [1,2,5,7,14–17]) are the human homolog of ripples in rodents[18]. Here, we simply followed the nomenclature of these prior studies in humans to label the identified events as "ripples," although it currently remains unclear to what extent they are gamma oscillations, epsilon oscillations, high gamma, or broadband gamma activity and to what extent they are contaminated with pathological ripples. An increasing number of human studies suggests that the events referred to as human ripples are involved in cognitive functions that have been traditionally assigned to ripples in rodents and other animals[19]. This suggests functional similarities of these analytically defined electrophysiological phenomena across species[18], but a number of factors complicate cross-species comparisons. For example, whereas the frequency band of ripples in rodents (about 150–250 Hz) is largely different from the frequency bands of gamma (about 30–90 Hz) and epsilon (about 90–150 Hz) oscillations[20], the ripple band in human studies (about 80–140 Hz) overlaps with the frequency ranges of gamma and epsilon oscillations. It could thus be the case that the transient high-frequency events that we referred to as ripples are actually pronounced gamma or epsilon oscillations. Furthermore, if human ripples indeed occur at frequencies of about 80–140 Hz, currently employed ripple-detection algorithms in human studies may actually detect a mixture of human ripples, gamma oscillations, and epsilon oscillations. Our ripples may also be contaminated or even be identical with (non-oscillatory) high gamma (at a broad range of frequencies above 80 Hz)[21] or broadband gamma activity[22] despite our efforts to mitigate this contamination by requiring ripple events to exhibit at least three oscillatory cycles and to show a global peak between 80 and 140 Hz in the relative power spectrum. For example, the ripples that we detected during the cue period and for which we found a positive relationship to memory performance in the subsequent retrieval period (Fig. 3b) might also constitute increased power of gamma or epsilon oscillations or increased high or broadband gamma activity (elicited by the visual presentation of the objects), for which a positive relationship with successful recall has been demonstrated before[23–27]. In addition, it is still unclear how physiological ripples can exactly be distinguished from pathological ripples in intracranial EEG recordings of human epilepsy patients[28]. In this study, we used a conservative rejection of IEDs and required ripple candidates to pass several criteria in order to increase the likelihood of only including physiological ripples in our analyses. Nevertheless, despite our best efforts these putatively physiological ripples may still have contained some pathological ripples. Future studies may identify more precise markers to differentiate between physiological and pathological ripples and thus enable the investigation of strictly physiological ripples in human epilepsy patients. For an extensive discussion of the above-mentioned and additional issues regarding ripples in humans, see ref. [18]. Bar plots show: bar, mean; error bar, ±SEM; gray dots, individual data points. A, amygdala; E, entorhinal cortex; H, hippocampus; P, parahippocampal cortex; T, temporal pole. ***$P < 0.001$.

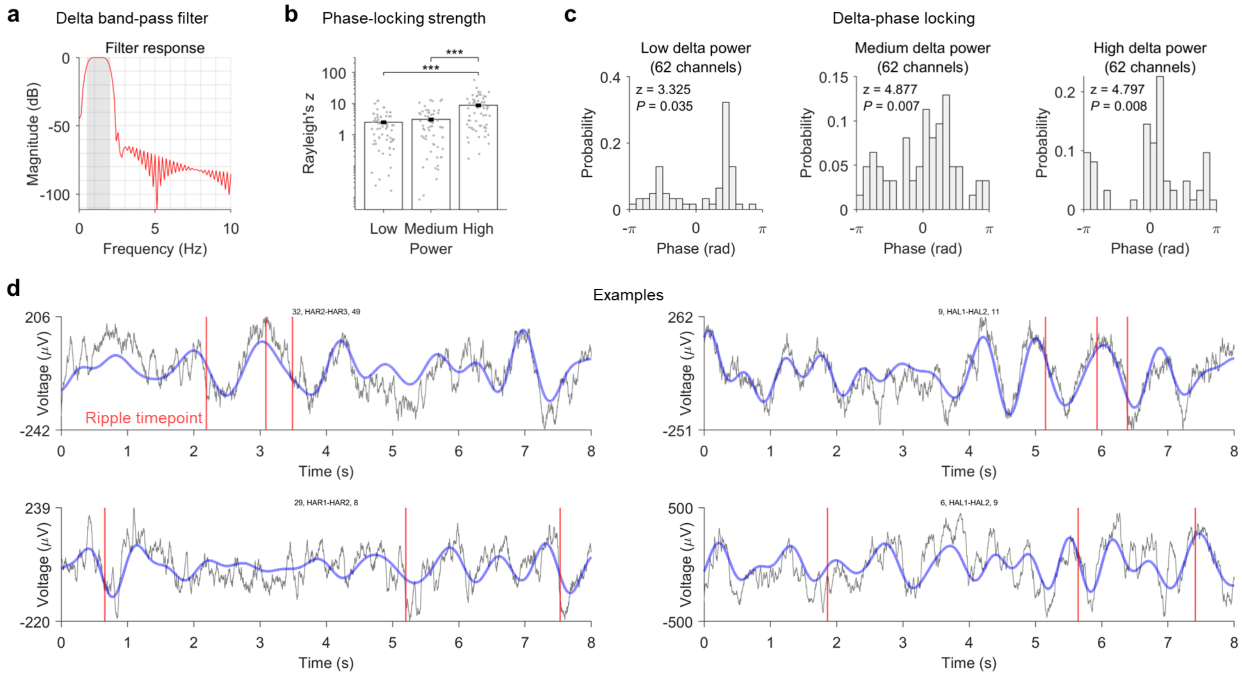

Fig. S7. Delta-phase locking of hippocampal ripples. **a**, Filter response (red line) of the band-pass filter for filtering the hippocampal signals in the delta-frequency range (0.5–2 Hz; gray area). Above 3 Hz, the signal is attenuated by a factor of more than 1000. **b**, Strength of phase locking of hippocampal ripples to delta activity, separately for delta phases associated with low, medium, and high delta power (n = 62 channels). Gray dots represent the Rayleigh z value for each hippocampal channel, quantifying how strongly the delta phases at hippocampal ripple peaks are clustered. Y axis is log-scaled. Phase locking is higher for high versus low delta power [two-sided paired t-test: $t(61) = 5.175$, $P < 0.001$, Bonferroni-corrected for three comparisons] and for high versus medium delta power [two-sided paired t-test: $t(61) = 5.036$, $P < 0.001$, Bonferroni-corrected for three comparisons]. Bar plots show: bar, mean; error bar, ±SEM; gray dots, individual data points. **c**, Preferred delta phases of hippocampal ripples (one preferred phase per channel, averaged across ripples), separately for low delta power (left), medium delta power (middle), and high delta power (right). Statistics (z- and P-values) are from Rayleigh tests. **d**, Examples of the relationship between hippocampal ripples and delta activity from four different hippocampal channels. These examples suggest that delta oscillations were present in this data and that ripples were indeed phase-locked to delta oscillations. We note though that slow-frequency components that are correlated with ripples may also come from superimposed waveforms rather than from genuine delta oscillations. Gray line, raw signal (not filtered); blue trace, band-pass filtered signal (0.5–2 Hz); red vertical lines, timepoints of ripple peaks. ***$P < 0.001$.

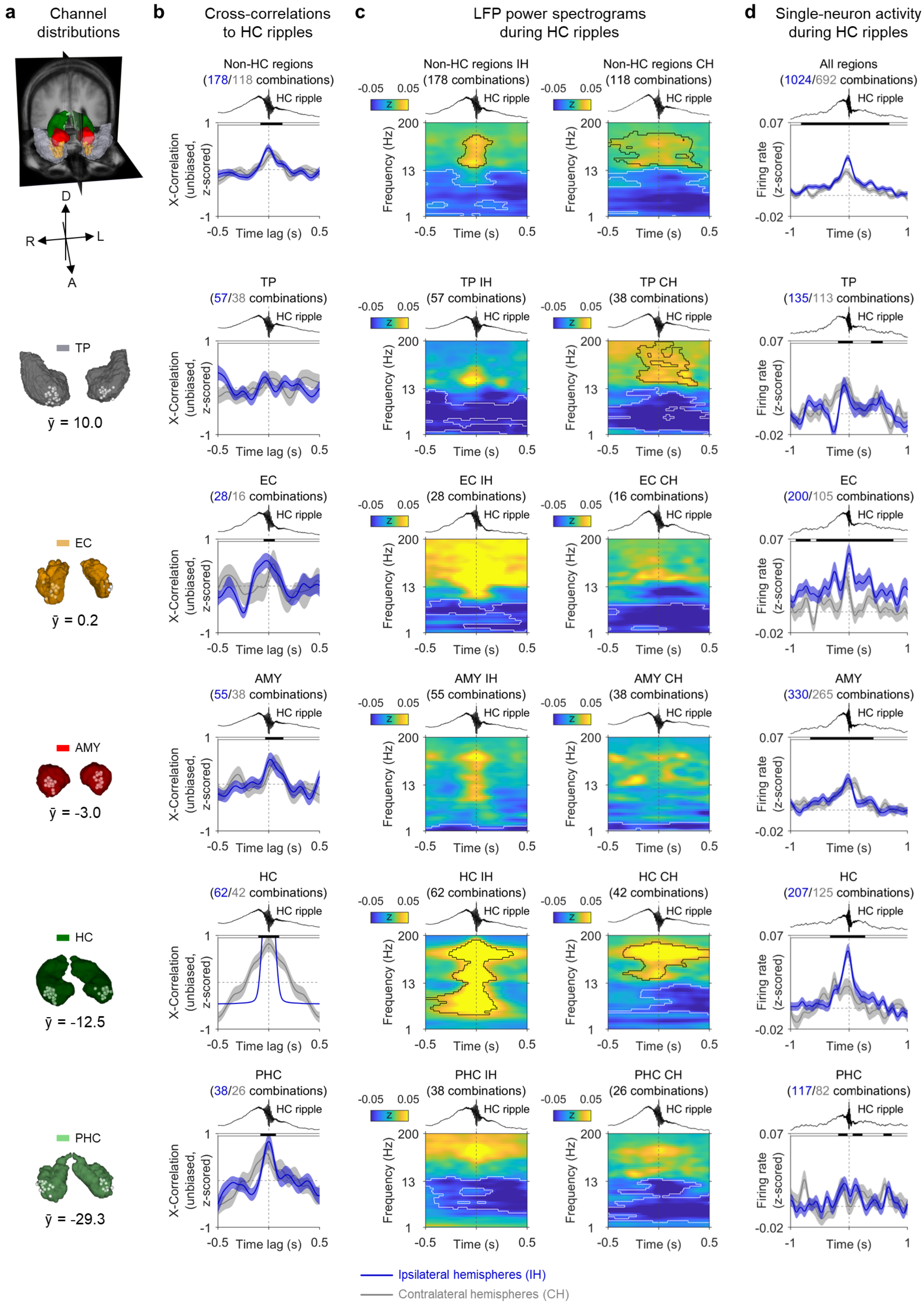

Fig. S8. Hippocampal ripples are associated with changes in LFP power and firing rates across the human MTL. a, Probabilistic visualizations of regions of interest including temporal pole (TP), entorhinal cortex (EC), amygdala (AMY), hippocampus (HC), and parahippocampal cortex (PHC), listed according to their anterior–to–posterior position in the human brain. White dots, locations of bipolar electrode contacts pooled across subjects; ȳ, average MNI y-value of the contacts in a given region of interest. **b**, Cross-correlations between hippocampal ripples and ripples in the different MTL regions. Blue and gray numbers indicate the number of channel pairs from ipsilateral and contralateral hemispheres, respectively. Time 0 indicates the peak of the hippocampal ripples; cross-correlation maxima at positive time lags indicate that the ripples from a particular region of interest numerically precede hippocampal ripples. Cross-correlations are smoothed with a Gaussian kernel of 0.2-s duration and are normalized by *z*-scoring cross-correlation values over the displayed time lags of ±0.5 s. Shaded region, mean ± SEM across channel pairs. Black shadings at top indicate *z*-scored cross-correlations from both ipsilateral and contralateral channel pairs being significantly above 0 (one-sided cluster-based permutation tests across the entire depicted time window: *P*

< 0.05). The blue line in the subpanel for hippocampal channels is the smoothed temporal autocorrelation and is only shown for the sake of completeness. **c**, Time-frequency resolved LFP power (*z*-scored relative to the entire experiment) in the different MTL regions during hippocampal ripples, both for ipsilateral channel pairs (left column) and contralateral channel pairs (right column). Power values are smoothed over time with a Gaussian kernel of 0.2-s duration. Time 0 indicates the peak of the hippocampal ripples. Black contours, significantly increased power; white contours, significantly decreased power (two-sided cluster-based permutation tests across the entire depicted time window: $P < 0.025$). **d**, Neuronal firing rates (*z*-scored relative to the entire experiment) in hippocampal and extrahippocampal regions (recorded using microelectrodes) during hippocampal ripples (recorded using macroelectrodes). Firing rates are smoothed over time with a Gaussian kernel of 0.2-s duration. Shaded region, mean ± SEM across neuron–ripple-channel combinations. Blue and gray numbers indicate the counts of ipsilateral and contralateral neuron–ripple-channel pairs, respectively. Black shadings at top indicate firing rates from both ipsilateral and contralateral neuron–ripple-channel combinations significantly above 0 (one-sided cluster-based permutation tests across the entire depicted time window: $P < 0.05$). CH, contralateral hemispheres; IH, ipsilateral hemispheres. AMY, amygdala; EC, entorhinal cortex; HC, hippocampus; PHC, parahippocampal cortex; TP, temporal pole. A, anterior; D, dorsal; L, left; R, right. X-Correlation, cross-correlation. Together with Fig. 4 and Fig. S9, these results show that hippocampal ripples did not only coincide with highly specific coactivations of object cells and place cells, but that they were also associated with broad increases in neural activity across the human MTL. Specifically, we observed that hippocampal ripples occurred simultaneously with ripples in extrahippocampal MTL regions; that they were associated with elevated high-frequency power and decreased low-frequency power in these regions; and that they were coupled to increased single-neuron spiking across the human MTL. Such brain states of increased excitation may provide a basis for establishing and activating connections between previously unconnected neurons. Hippocampal ripples may thus support various cognitive functions that rely on interactions between separate and widespread neural representations. These findings of wide-ranging neural changes during hippocampal ripples align with previous reports in both animals and humans. In rodents, hippocampal sharp-wave ripples are accompanied by widespread cortical and subcortical activations[29–31] and coincide with transient brain-wide increases in functional connectivity[32]. In humans, hippocampal ripples are coupled with ripples in many cortical areas[14,15,33] and are accompanied by increased high-frequency broadband activity in widely distributed neocortical sites[16]. They coincide with memory-specific high-frequency broadband activity in high-order visual areas during memory retrieval[2] and support the reinstatement of memory-specific single-neuron sequences in the temporal cortex[1]. These studies support our conclusion that ripples in the hippocampus provide a time window for increased activation and excitation across the brain, which may support associative memory by establishing and reactivating connections between separate neural elements.

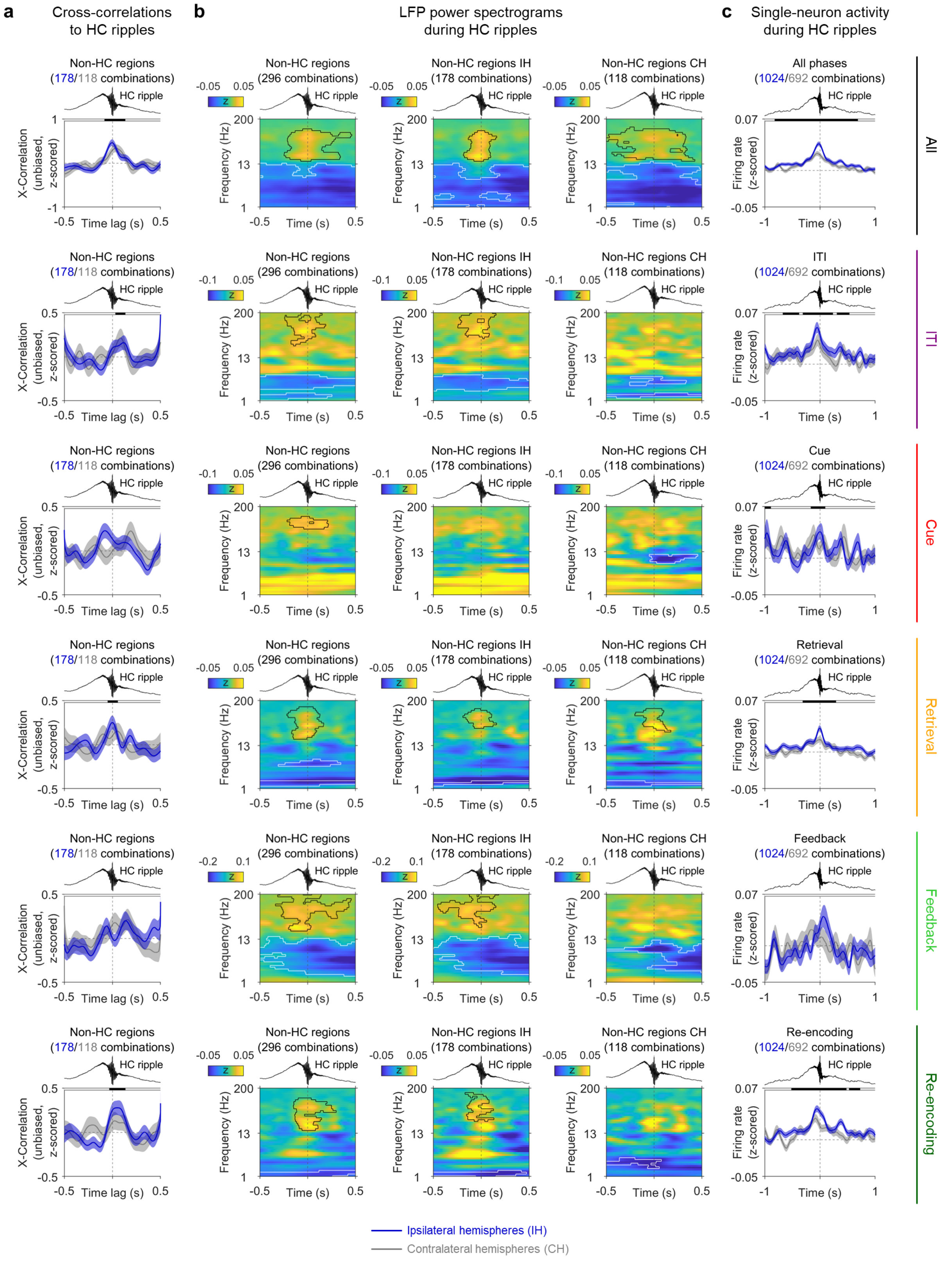

Fig. S9. Hippocampal ripples are associated with changes in LFP power and firing rates in the human MTL across different trial phases. a, Cross-correlations between hippocampal ripples and ripples in non-hippocampal MTL regions (amygdala, entorhinal cortex, parahippocampal cortex, temporal pole). Blue and gray numbers indicate the number of channel pairs from ipsilateral and contralateral hemispheres, respectively. Time 0 indicates the peak of the hippocampal ripples; cross-correlation maxima at positive time lags indicate that the ripples from a particular region of interest numerically precede hippocampal ripples. Cross-correlations are smoothed with a Gaussian kernel of 0.2-s duration and are normalized by *z*-scoring cross-correlation values over the displayed time lags of ±0.5 s. Shaded region, mean ± SEM across channel pairs. Black shadings at top indicate *z*-scored cross-correlations from both ipsilateral and contralateral channel pairs being significantly above 0 (one-sided cluster-based permutation tests across the entire depicted time window: $P < 0.05$). **b**, Time-frequency resolved LFP power (*z*-scored relative to the entire experiment and baseline-corrected relative to -2.5 to -0.5s before the ripple peak) in non-

hippocampal MTL regions during hippocampal ripples, showing the results for all channel pairs (left column), ipsilateral channel pairs (middle column), and contralateral channel pairs (right column). Power values are smoothed over time with a Gaussian kernel of 0.2-s duration. Time 0 indicates the peak of the hippocampal ripples. Black contours, significantly increased power; white contours, significantly decreased power (two-sided cluster-based permutation tests across the entire depicted time window: $P < 0.025$). **c**, Neuronal firing rates (z-scored relative to the entire experiment) in hippocampal and extrahippocampal regions (recorded using microelectrodes) during hippocampal ripples (recorded using macroelectrodes). Firing rates are smoothed over time with a Gaussian kernel of 0.2-s duration. Shaded region, mean ± SEM across neuron–ripple-channel combinations. Blue and gray numbers indicate the counts of ipsilateral and contralateral neuron–ripple-channel pairs, respectively. Black shadings at top indicate firing rates from both ipsilateral and contralateral neuron–ripple-channel combinations significantly above 0 (one-sided cluster-based permutation tests across the entire depicted time window: $P < 0.05$). Different rows of the figure show the results from ripples that occurred in specific trial phases (all trial phases; ITI; cue; retrieval; feedback; re-encoding). CH, contralateral hemispheres; IH, ipsilateral hemispheres; ITI, inter-trial interval; X-Correlation, cross-correlation.

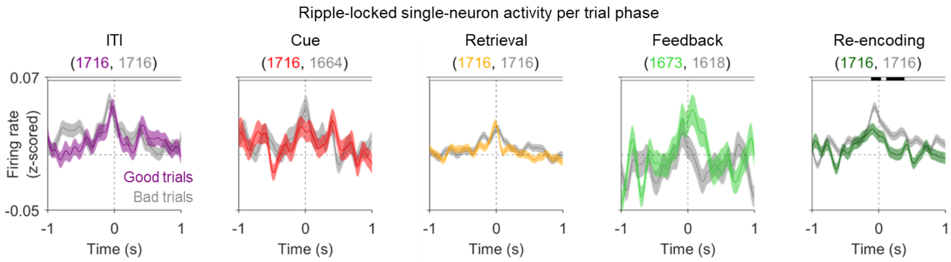

Fig. S10. Neuronal firing rates during hippocampal ripples occurring in specific trial phases. Our analyses in the main text showed that hippocampal ripples were associated with a state of increased neural activations across the human MTL (Fig. 4), including a general increase of single-neuron firing during hippocampal ripples (Fig. 4d). To further understand the functional relevance of this recruitment of neuronal activity during hippocampal ripples, we asked whether ripple-related single-neuron firing would differ as a function of trial phase and the subjects' memory performance (using a median split of each subject's memory performance values across trials). We found that ripple-locked firing rates during re-encoding periods following inferior memory responses were significantly increased as compared to re-encoding periods after better memory responses (cluster-based permutation test: $t_{cluster1} = -357.511$, $P = 0.002$, 0.113–0.385 s relative to the ripple peaks; $t_{cluster2} = -206.887$, $P = 0.023$, -0.115–0.033 s; $n = 1716$ neuron–ripple-channel combinations). In the other trial phases (ITI, cue, retrieval, and feedback), ripple-locked firing rates exhibited similar increases during trials with better as compared to inferior memory performance. Together with the increased ripple rates during re-encoding periods following inferior memory retrieval (Fig. 3), this elevated single-neuron firing supports the idea that neuronal resources are broadly recruited in situations following inferior memory performance—potentially to newly establish or update existing memory representations. Firing rates are smoothed with a Gaussian kernel of 0.2-s duration and *z*-scored relative to the entire experiment. Colored lines and shadings (mean ± SEM) indicate firing rates during ripples from trials with good memory performance; gray lines and shadings (mean ± SEM) indicate firing rates during ripples from trials with bad memory performance. Time 0, hippocampal ripple peak. Black shadings at the top of the subpanels indicate significantly different firing rates during ripples from good-performance trials versus firing rates during ripples from bad-performance trials (two-sided cluster-based permutation tests across the entire depicted time window: $P < 0.025$). Colored numbers indicate the number of neuron–ripple-channel combinations contributing to the data. ITI, inter-trial interval.

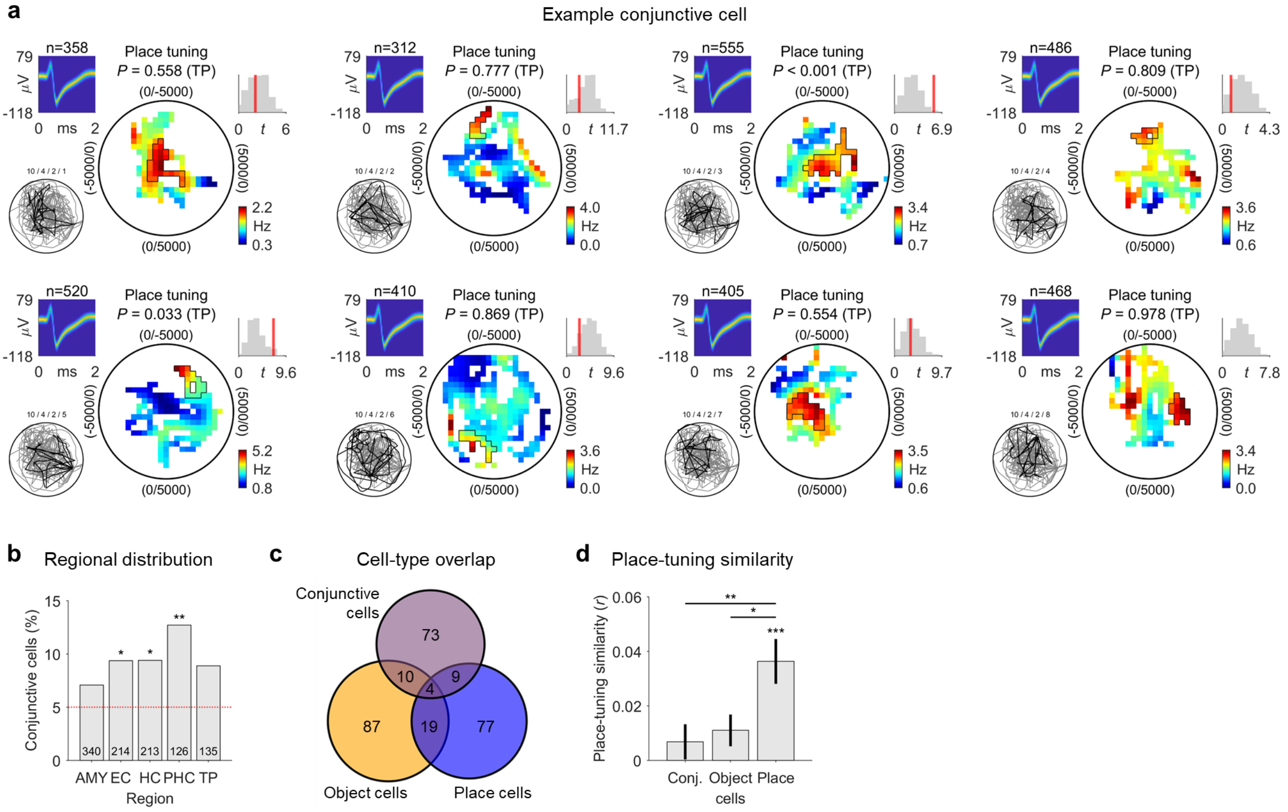

Fig. S11. Conjunctive cells. **a**, Example conjunctive cell. The eight different panels show the cell's place tuning when only considering data from trials with a particular object. This example conjunctive cell exhibited significant place tuning at a Bonferroni-corrected alpha-level of α = 0.05/8 only during trials with object #3 (first row, third column). For each panel, from left to right: action potentials as density plot; entire (gray line) and object-specific (black line) navigation path of the subject through the environment; smoothed firing-rate map (unvisited areas are shown in white); empirical *t*-statistic from a two-sided two-sample *t*-test (red line) and surrogate *t*-statistics (gray histogram); color bar, firing rate. Candidate place fields are outlined in black. **b**, Distribution of conjunctive cells across brain regions; red line, 5% chance level. Conjunctive cells were significantly prevalent in the entorhinal cortex, hippocampus, and parahippocampal cortex (two-sided binomial tests versus chance with Bonferroni correction for multiple comparisons: $P_{corr.} = 0.035$, $P_{corr.} = 0.034$, and $P_{corr.} = 0.003$, respectively). *$P_{corr.} < 0.05$; **$P_{corr.} < 0.01$. **c**, Overlap between object cells, place cells, and conjunctive cells. The large majority of object and place cells were not also conjunctive cells. **d**, Similarity of place tuning across trials with the eight different objects, which we estimated using pairwise Pearson correlations between the firing-rate maps from trials with different objects. Place tuning was significantly similar (and thus stable) across trials with different objects for place cells [two-sided one-sample *t*-test: $t(108) = 4.417$, $P < 0.001$], but not for conjunctive cells [$t(95) = 1.056$, $P = 0.294$] and also not for object cells [$t(119) = 1.888$, $P = 0.061$]. Place-tuning similarity was significantly higher in place cells than in conjunctive cells [two-sided two-sample *t*-test: $t(203) = 2.772$, $P = 0.006$] and also significantly higher in place cells than in object cells [two-sided two-sample *t*-test: $t(227) = 2.550$, $P = 0.011$]. These results underline the general spatial coding of place cells (being stable across time irrespective of the current object) and confirm that conjunctive cells (as well as object cells) do not exhibit such general spatial coding, as expected. In the main text we discussed two broader hypotheses on the possible neural implementation of associative memories (the "conjunctive hypothesis" and the "coactivity hypothesis"). Both hypotheses align with the common view that the hippocampus holds a crucial function in binding the separate elements of associative memories together[33–35]. In addition to these two hypotheses, associative memory presumably rests upon multiple other neural mechanisms. In particular, several recent studies indicate that the lateral entorhinal cortex holds a crucial function in associative memory, as rats with lesions to the lateral entorhinal cortex are unable to recognize object–context associations[36]. Furthermore, over the course of associative learning, oscillatory coupling at frequencies between 20–40 Hz evolves between the lateral entorhinal cortex and the dorsal hippocampus[37], suggesting that interregional (entorhinal–hippocampal) neural communication contributes to associative memory[38,39]. Specifically, fan cells of the lateral entorhinal cortex may be important for associative memory as inhibiting them optogenetically impairs the learning of new associative memories[40,41]. These studies provide strong evidence that cells and oscillations in the lateral entorhinal cortex are key components of the neural substrate of associative memories. Some of our object and place cells may have been located in the lateral entorhinal cortex and this brain region may thus have contributed to our coactivity results. *$P < 0.05$; **$P < 0.01$; ***$P < 0.001$. Conj., conjunctive. AMY, amygdala; EC, entorhinal cortex; HC, hippocampus; PHC, parahippocampal cortex; TP, temporal pole.

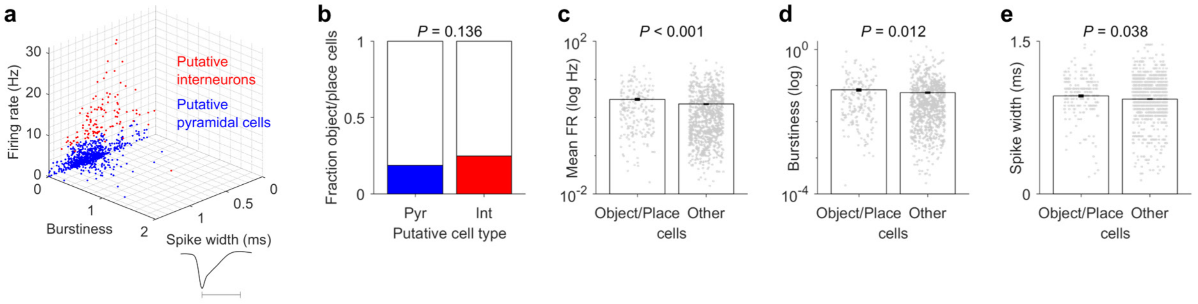

Fig. S12. Putative pyramidal cells and interneurons and their relationship to object and place cells. a, Separation of putative pyramidal cells (blue dots; $n = 946$) and putative interneurons (red dots; $n = 117$) using *k*-means clustering based on the three parameters mean firing rate, burstiness, and spike width[42,43]. Note though that the cells showed a continuum of values rather than forming two clearly distinct classes. Mean firing rate was estimated as the total number of spikes divided by the total duration of the recording. Burstiness was calculated by dividing the total number of spikes that occurred at less than 10 ms of each other by to the total number of spikes that occurred at greater than or equal to 10 ms of each other. Spike width was estimated as the duration between the global minimum of the average waveform of the cell and its maximum after the global minimum (for an illustration, see the extent of the gray line in the inset below "Spike width"). **b**, Cells identified as object or place cells (Figs. 5 and 6; $n = 206$) were similarly prevalent among putative pyramidal cells ("Pyr") and putative interneurons ("Int") (two-sided Fisher's exact test: $P = 0.136$). **c**, Cells identified as object or place cells exhibited higher firing rates than other cells [object/place cells: 3 ± 0.259 Hz (mean ± SEM); other cells: 2.254 ± 0.118 Hz; two-sided Wilcoxon rank sum test: $z = 4.250$, $P < 0.001$]. **d**, Cells identified as object or place cells exhibited higher burstiness than other cells [object/place cells: 0.076 ± 0.008; other cells: 0.064 ± 0.004; two-sided Wilcoxon rank sum test: $z = 2.505$, $P = 0.012$]. **e**, Cells identified as object or place cells exhibited longer spike widths than other cells [object/place cells: 0.962 ± 0.014 ms; other cells: 0.931 ± 0.008 ms; two-sided Wilcoxon rank sum test: $z = 2.078$, $P = 0.038$]. Bar plots in **c–e** show: bars, means; black vertical lines, ±SEM; gray dots, individual cells. FR, firing rate.

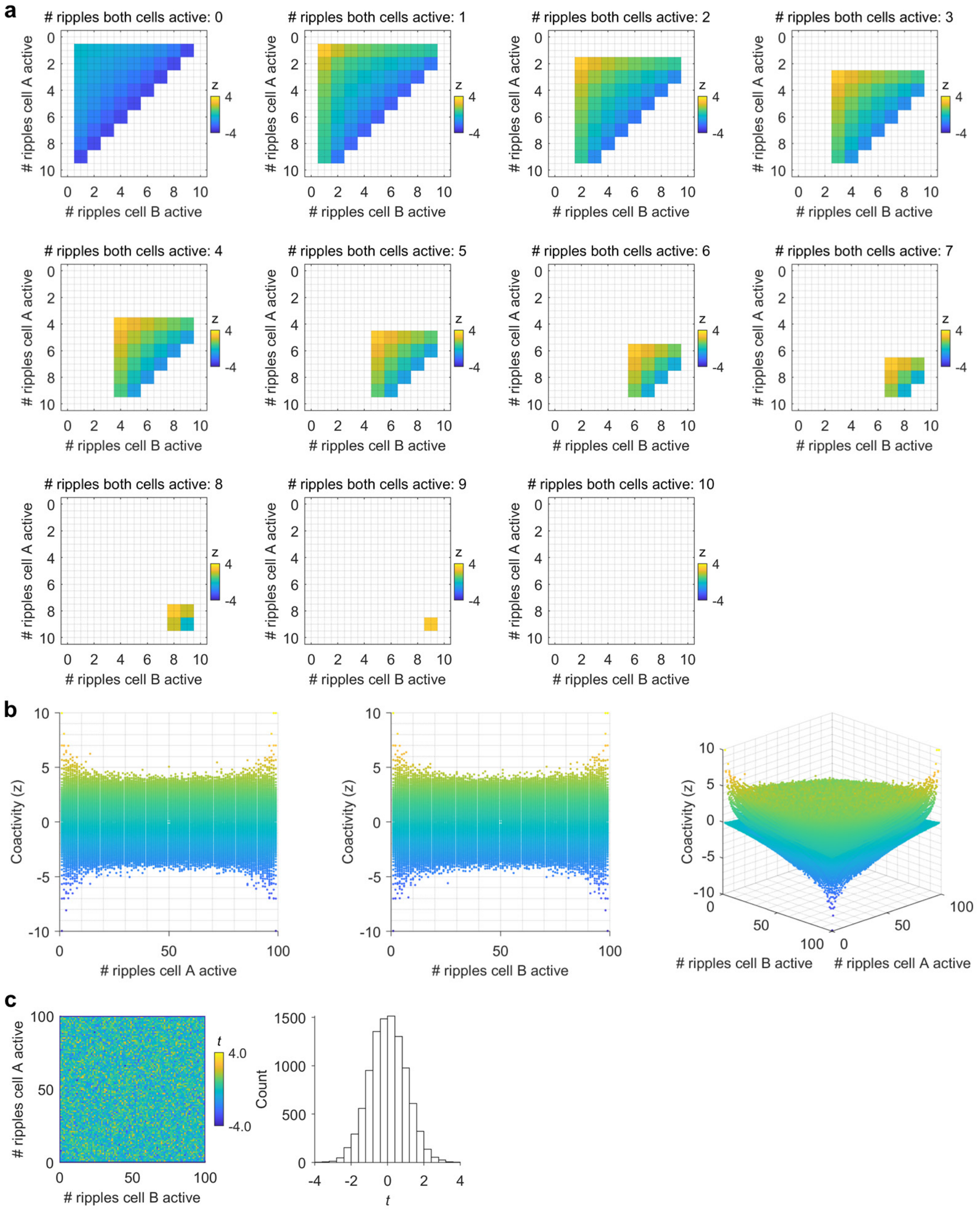

Fig. S13. Illustration of the coactivity *z*-score. a, Coactivity *z*-scores between two cells (cell A and cell B) in a hypothetical situation with ten ripples. The different subplots display the coactivity *z*-scores as a function of the number of ripples in which cell A is active (y-axis), the number of ripples in which cell B is active (x-axis), and the number of ripples in which both cells are active (title). High coactivity *z*-scores are achieved when the number of ripples in which both cells are active matches the number of ripples in which cell A (irrespective of cell B) or cell B (irrespective of cell A) is active. The coactivity *z*-value is not defined (white areas) for situations (i) in which cell A and/or cell B are not active during any ripple; (ii) in which cell A and/or cell B are active during all ripples; and (iii) in which the activity combination is logically impossible. **b**, Relationship between the coactivity *z*-score and the number of ripples during which cell A is active and during which cell B is active (in a hypothetical scenario with 100 ripples). For each combination of "# ripples cell A active" and "# ripples cell B active," 1000 coactivity *z*-scores were estimated by randomly drawing the ripple-related activations of cell A and cell B. This simulation illustrates that there is no systematic relationship between a cell's activity level and the resulting coactivity *z*-score. **c**, Left: For each combination of "# ripples cell A active" and "# ripples cell B active," we performed a one-sample *t*-test to identify whether the 1000 coactivity *z*-scores were systematically different from 0. The randomness of the *t*-map shows that there is no systematic relationship between a cell's activity level and the resulting coactivity *z*-score. 4.7% of the *t*-values were significant at $P < 0.05$, which is at chance level (5%). Right: histogram of all *t*-values from the *t*-map on the left side, showing that the *t*-values are not systematically shifted toward positive or negative values. #, number.

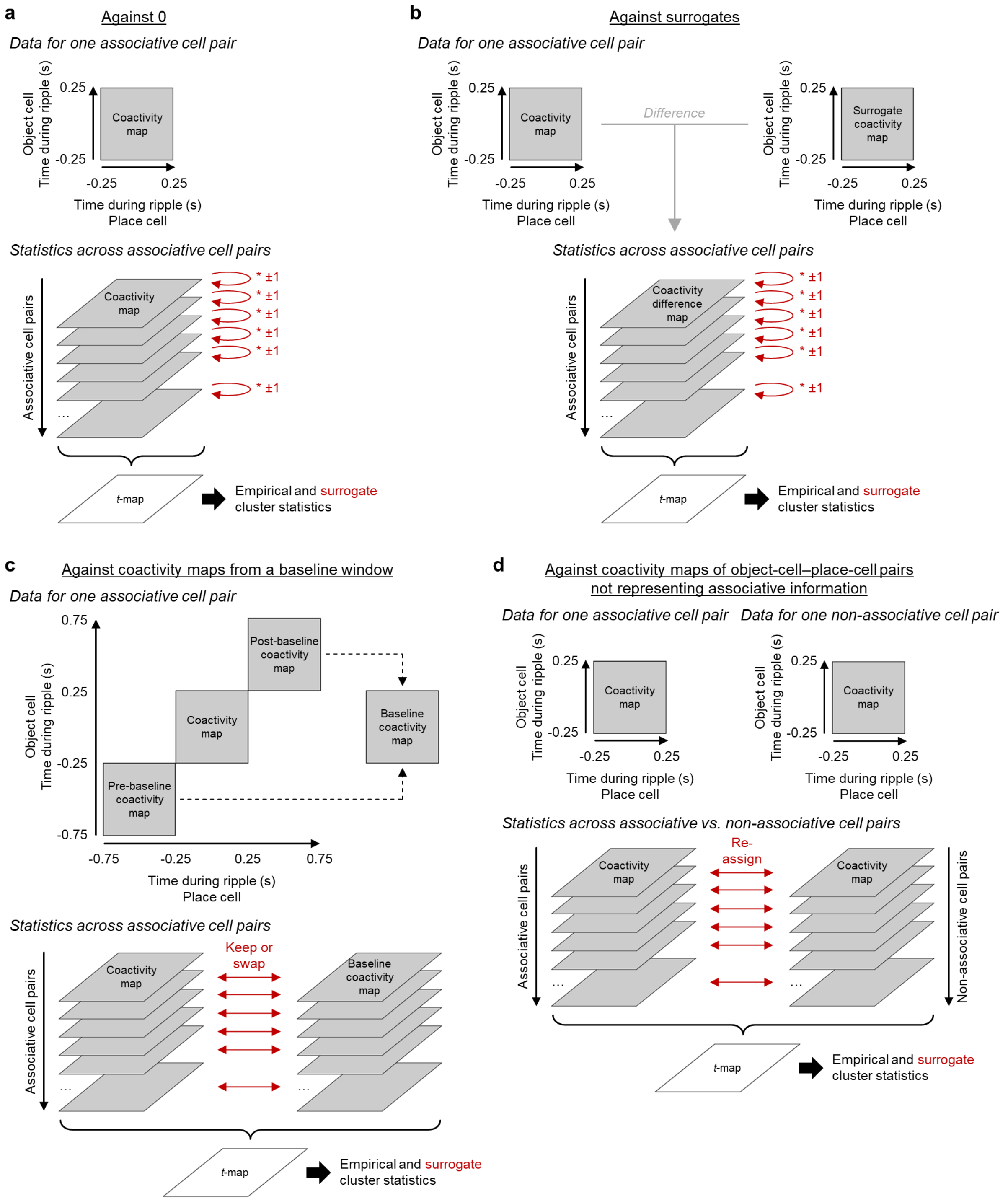

Fig. S14. Illustration of the cluster-based permutation tests to evaluate ripple-locked coactivity. a, Cluster-based permutation test of the coactivity maps against chance (i.e., 0). To identify empirical clusters, a one-sample *t*-test against 0 was performed for each time-by-time bin. Contiguous clusters of significant bins were identified, and the corresponding *t*-values were summed, resulting in empirical cluster statistics. To create surrogate data, a randomly selected subset of the coactivity maps was multiplied by -1. Performing the same steps as for the empirical data, surrogate cluster statistics were then created based on the surrogate data. **b**, Variant of the cluster-based permutation test against chance, where a surrogate coactivity map was subtracted from each corresponding empirical coactivity map. The surrogate coactivity of a given cell pair was estimated by circularly shifting the ripple-locked activity levels of the object cell relative to the ripple-locked activity levels of the place cell by a random number of ripples before calculating the two-dimensional coactivity map. The statistics across associative cell pairs were performed as in panel a. **c**, Cluster-based permutation test of the coactivity maps against coactivity maps from a baseline window. The pre-baseline and post-baseline windows spanned 0.75 to 0.25 s before and 0.25 to 0.75 s after the ripple peaks, respectively. Baseline coactivity maps were then estimated by averaging the pre-baseline and the post-baseline coactivity maps. To identify empirical cluster statistics, a two-sample *t*-test between the coactivity maps and the baseline coactivity maps was performed for each time-by-time bin. Contiguous clusters of significant bins were identified, and the corresponding *t*-values were summed up, resulting in empirical cluster statistics. To create surrogate data, a random subset of corresponding coactivity maps and baseline coactivity maps was swapped. Performing the same steps as for the empirical data, surrogate cluster statistics

were then created based on the surrogate data. **d**, Cluster-based permutation test of the coactivity maps of associative cell pairs versus non-associative cell pairs (for which the location of the preferred object of the object cell was not located inside the place field of the place cell). To identify empirical clusters, a two-sample *t*-test between the coactivity maps of associative cell pairs and those of non-associative cell pairs was performed for each time-by-time bin. Contiguous clusters of significant time bins were identified, and the corresponding *t*-values were summed up, resulting in empirical cluster statistics. To create surrogate data, the coactivity maps of associative and non-associative cell pairs were randomly reassigned to the two cell groups. Performing the same steps as for the empirical data, surrogate cluster statistics were then created based on the surrogate data. In all different tests, empirical clusters were considered significant if their cluster statistics exceeded the 95th percentile of all surrogate cluster statistics. Red arrows and numbers indicate how surrogate data were created.

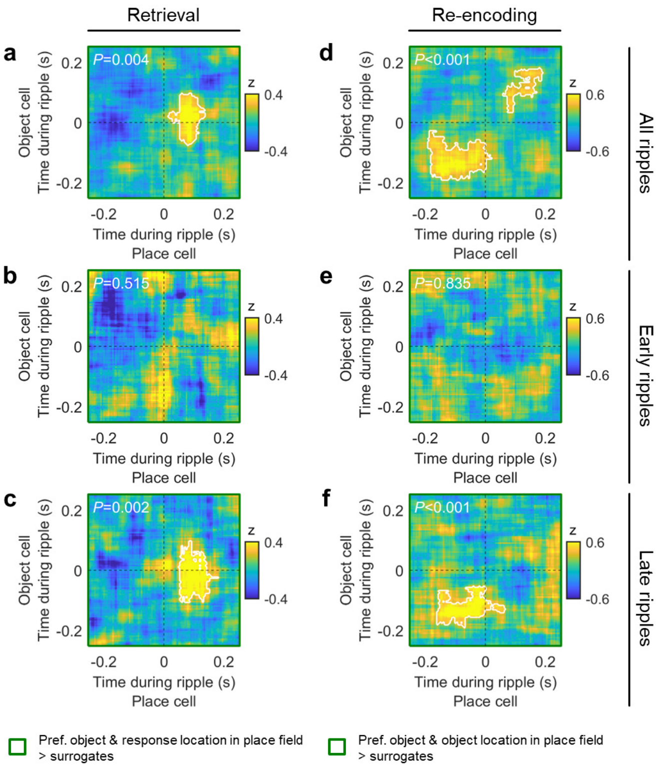

Fig. S15. Coactivity of object cells and place cells during hippocampal ripples: comparison against surrogate coactivity maps. a–f, Same as the left panels in Fig. 7c–h, with the difference that the coactivity maps are contrasted against surrogate coactivity maps instead of against 0. For each cell pair, one surrogate coactivity map was estimated by circularly shifting the spiking activity of the object cell relative to the spiking activity of the place cell by a random number of ripples. White lines in **a–f** delineate significant clusters based on one-sided cluster-based permutation tests, whose *P*-values are stated in the upper left corners of the coactivity maps. >, larger than; pref., preferred.

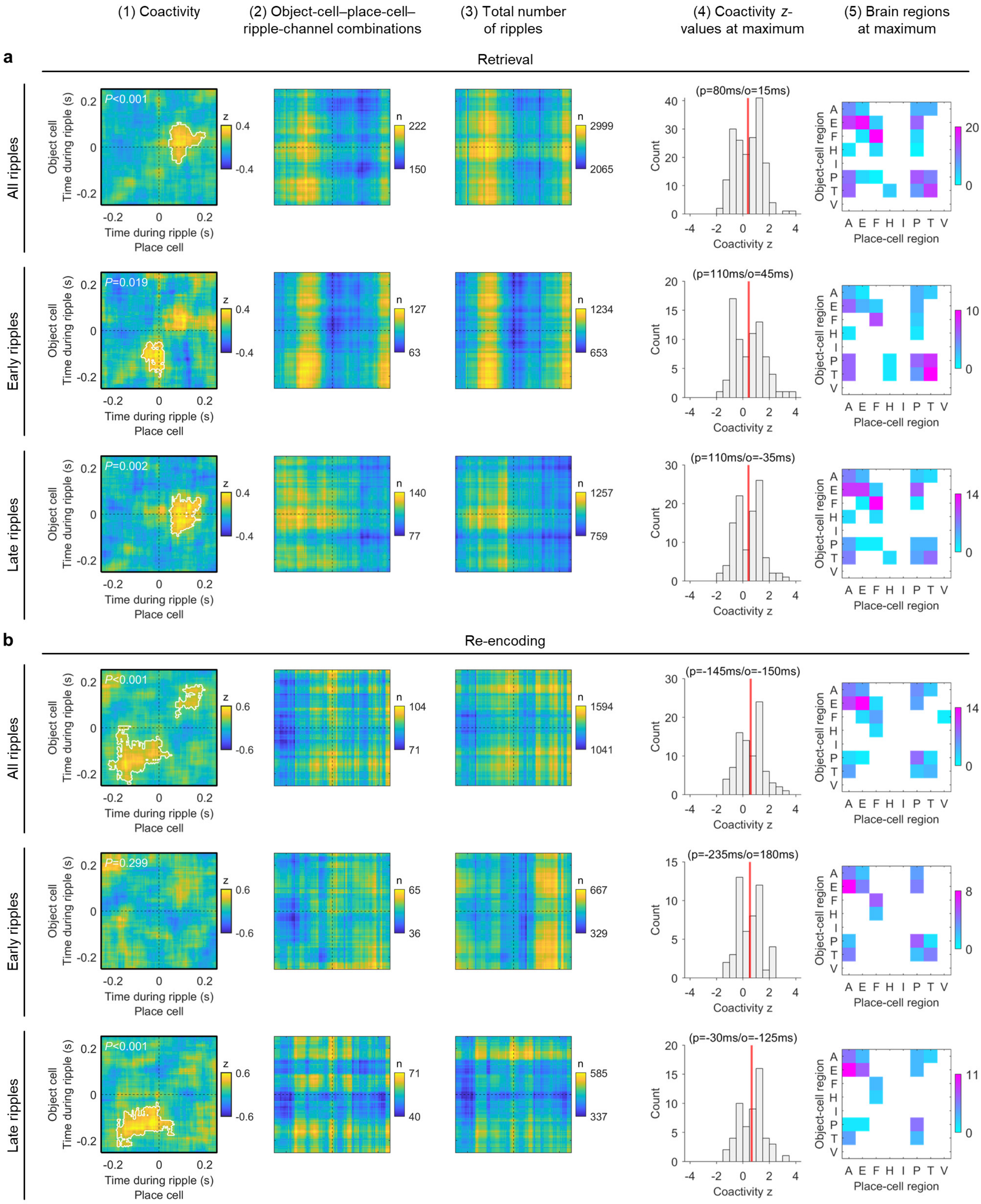

Fig. S16. Coactivity of object cells and place cells during hippocampal ripples: additional information. Columns from left to right: (**1**) Coactivity maps reproduced from Fig. 7c–h, left column. White lines in **a** and **b** delineate significant clusters based on one-sided cluster-based permutation tests, which control for multiple comparisons and whose *P*-values are stated in the upper left corners of the coactivity maps. (**2**) Number of object-cell–place-cell–ripple-channel combinations underlying the coactivity maps in the first column. (**3**) Number of ripples underlying the coactivity maps in the first column, pooled across object-cell–place-cell–ripple-channel combinations. (**4**) Histograms of individual coactivity *z*-values underlying the global maxima in the coactivity maps in the first column. Red line, mean; numbers above the histograms indicate the temporal coordinates of the global maxima in the coactivity maps (p, temporal coordinate for the place-cell time axis; o, temporal coordinate for the object-cell time axis). (**5**) Brain regions of the object cells and place cells contributing the coactivity *z*-values of the global peaks of the coactivity maps in the first column; color bars, number of object-cell–place-cell combinations. **a**, Data for retrieval. **b**, Data for

re-encoding. A, amygdala; E, entorhinal cortex; F, fusiform gyrus; H, hippocampus; I, insula; P, parahippocampal cortex; T, temporal pole; V, visual cortex.

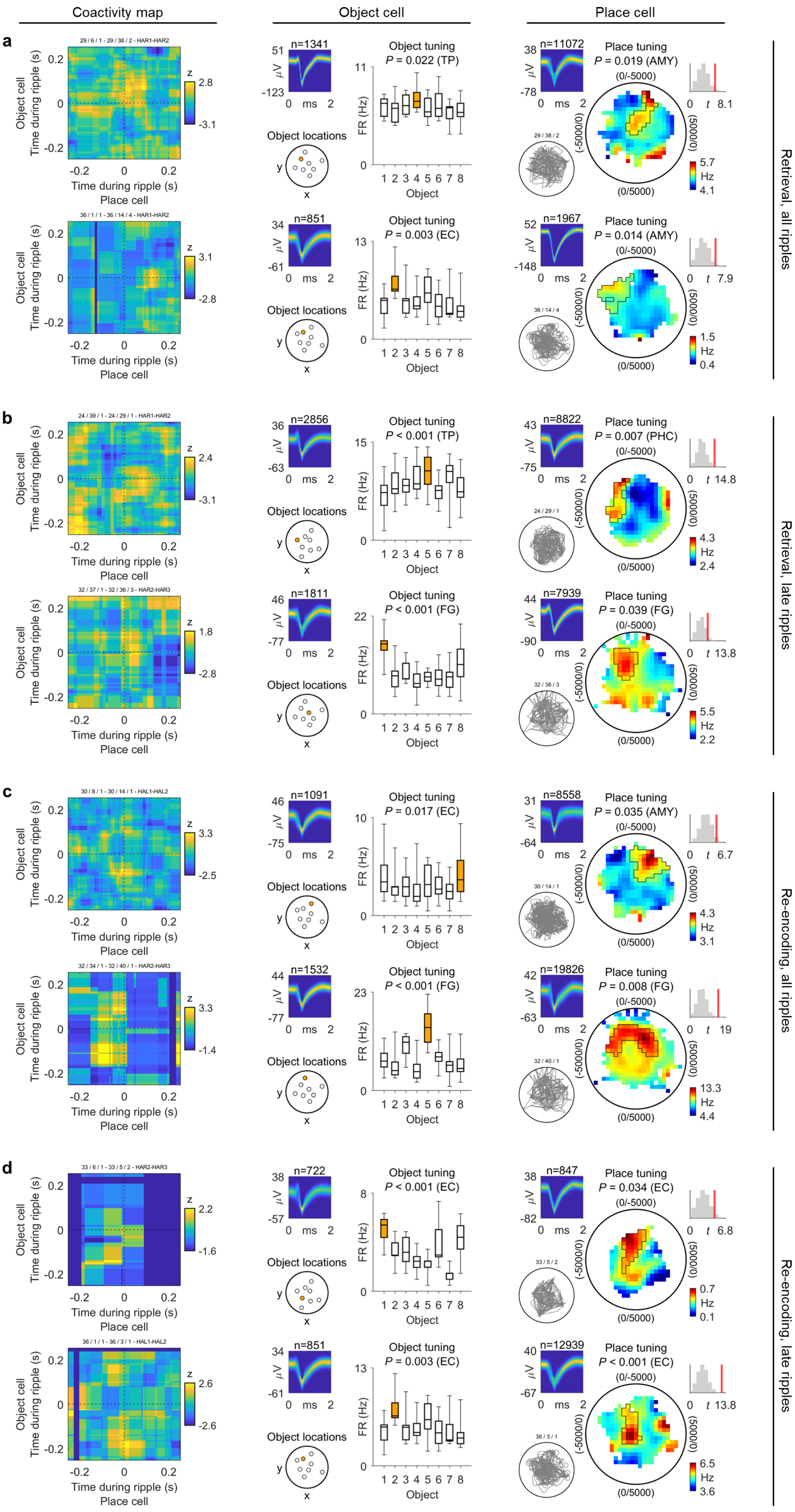
Coactivity map
Object cell
Place cell
a
b
c
d
Retrieval, all ripples
Retrieval, late ripples
Re-encoding, all ripples
Re-encoding, late ripples
Object cell Time during ripple (s)
Time during ripple (s)
Place cell
Object locations
Object tuning
P = 0.022 (TP)
P = 0.003 (EC)
P < 0.001 (TP)
P < 0.001 (FG)
P = 0.017 (EC)
P < 0.001 (FG)
P < 0.001 (EC)
P = 0.003 (EC)
FR (Hz)
Object
Place tuning
P = 0.019 (AMY)
P = 0.014 (AMY)
P = 0.007 (PHC)
P = 0.039 (FG)
P = 0.035 (AMY)
P = 0.008 (FG)
P = 0.034 (EC)
P < 0.001 (EC)
(0/-5000)
(0/5000)
(-5000/0)
(5000/0)
n=1341
n=851
n=2856
n=1811
n=1091
n=1532
n=722
n=851
n=11072
n=1967
n=8822
n=7939
n=8558
n=19826
n=847
n=12939

Fig. S17. Coactivity of object cells and place cells during hippocampal ripples: individual examples. a, Both rows show an individual example of the coactivity of an object-cell–place-cell–ripple-channel combination, from ripples during the retrieval periods of the associative object–location memory task. Left column, coactivity map between a given object cell and a given place cell during the ripples of a given ripple channel. Middle column, information about the object cell including a density plot of the spike waveforms (upper left subpanel; number above subpanel indicates spike count), locations of the objects including the location of the preferred object (orange dot in the lower left subpanel), and absolute firing rates in response to the different objects during the cue periods (right subpanel). Box plots show: center line, median; box limits, upper and lower quartiles; whiskers, minimum and maximum. Object-cell statistics were derived based on n = 1341, 851, 2856, 1811, 1091, 1532, 722, and 851 action potentials, respectively. Right column, information about the place cell including a density plot of the spike waveforms (upper left subpanel), the subject's navigation path through the virtual environment (lower left subpanel), the cell's firing-rate map (middle subpanel), and the comparison between the cell's empirical test statistic and its surrogate test statistics (upper right subpanel). Color bar, firing rate. Place-cell statistics were derived based on n = 11072, 1967, 8822, 7939, 8558, 19826, 847, and 12939 action potentials, respectively. Object- and place-cell statistics were estimated using one-sided permutation tests. **b**, Same as in **a**, but for late retrieval-related ripples. **c**, Same as in **a**, but for ripples occurring during the re-encoding periods. **d**, Same as in **a**, but for late re-encoding-related ripples. AMY, amygdala; EC, entorhinal cortex; FG, fusiform gyrus; PHC, parahippocampal cortex; TP, temporal pole.

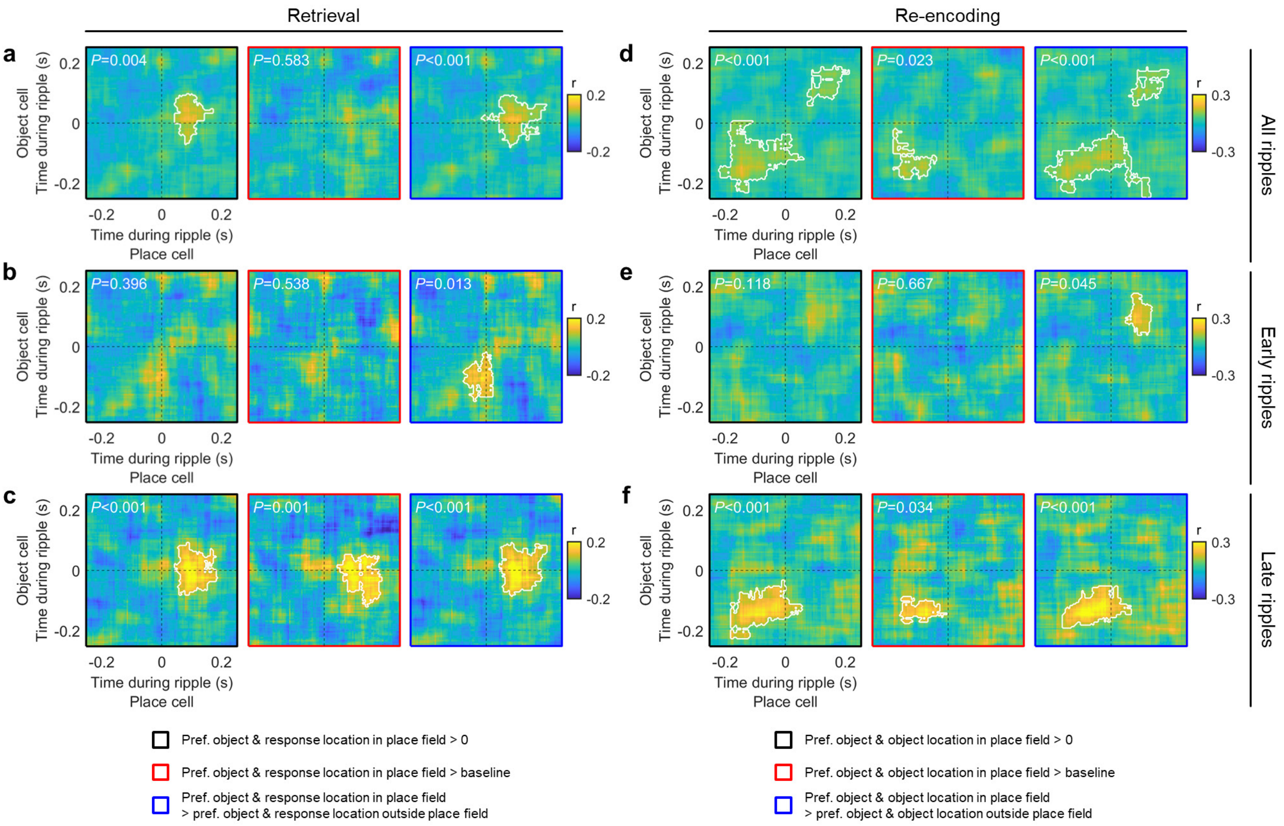

Fig. S18. Coactivity of object cells and place cells during hippocampal ripples: Pearson correlations. a–f, Same as Fig. 7c–h, with the difference that coactivity was estimated with Pearson correlations. White lines in **a–f** delineate significant clusters based on one-sided cluster-based permutation tests, with *P*-values stated at the upper left. >, larger than; pref., preferred.

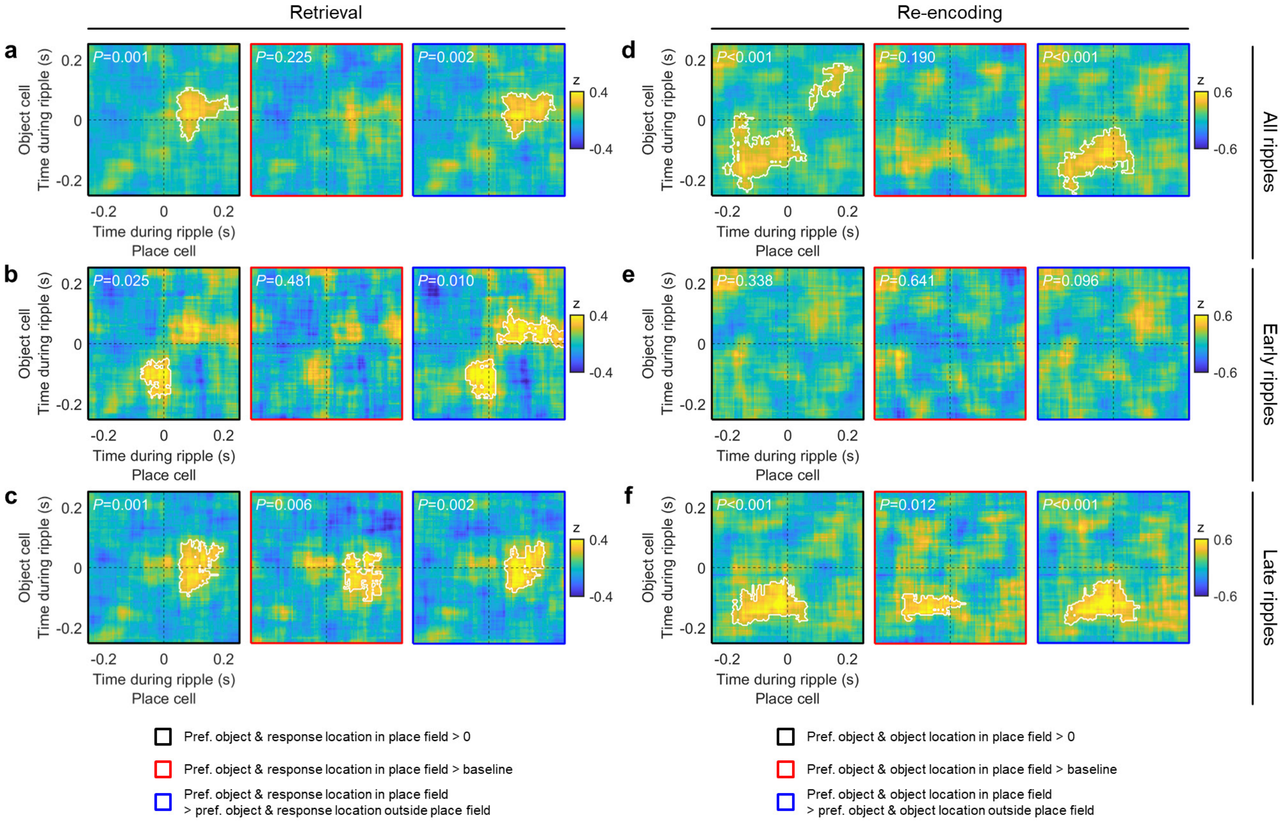

Fig. S19. Coactivity of object cells and place cells during hippocampal ripples: first sessions only. a–f, Same as Fig. 7c–h, with the difference that coactivity was estimated using data from first sessions only. White lines in **a–f** delineate significant clusters based on one-sided cluster-based permutation tests, with *P*-values stated at the upper left. >, larger than; pref., preferred.

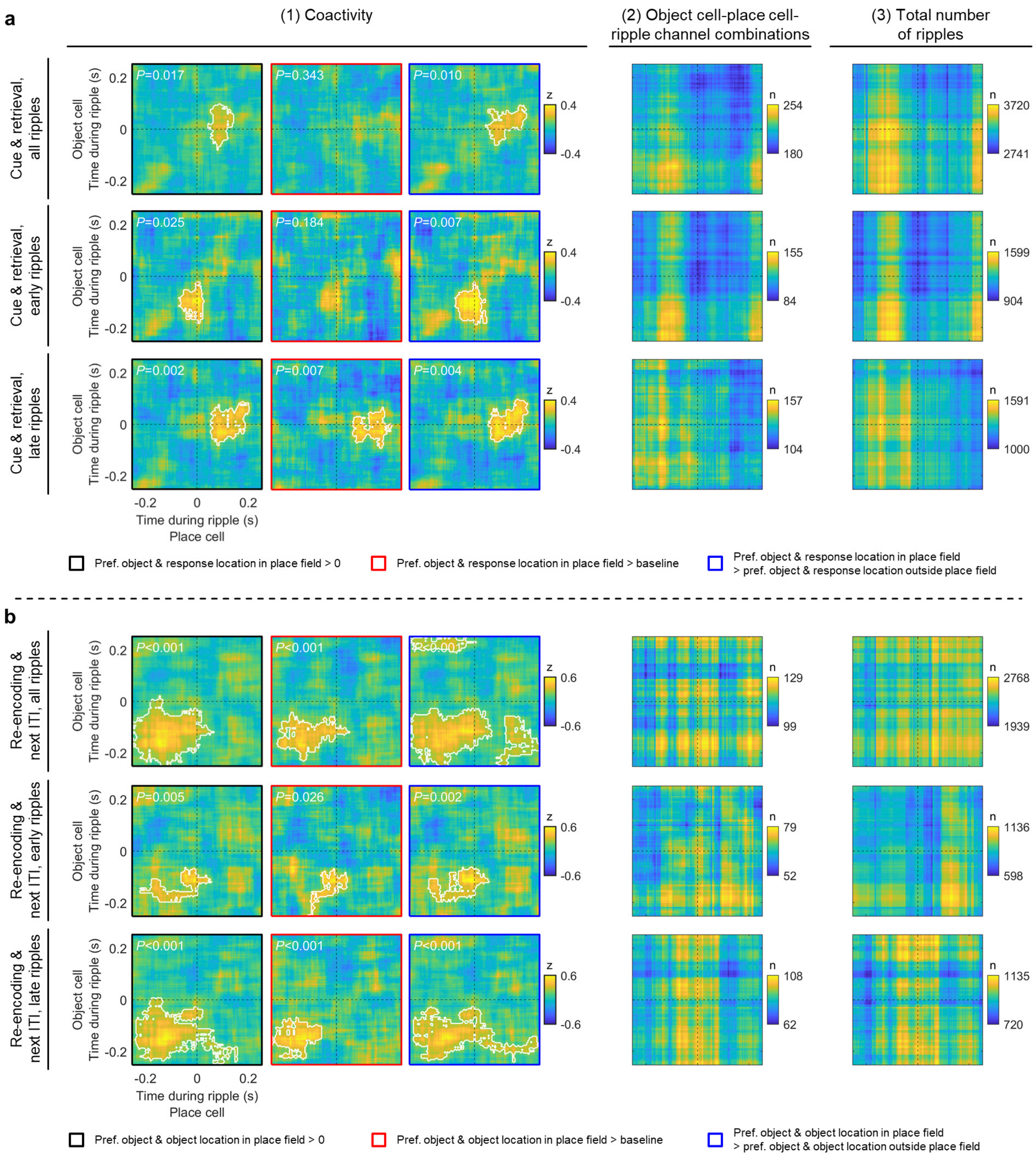

Fig. S20. Coactivity of object cells and place cells during hippocampal ripples: adding cue periods and inter-trial intervals (ITI). a1, Coactivity maps estimated using ripples during cue and retrieval phases, considering only trials in which the subject is asked to remember the location of the preferred object of the object cell and in which the subject's response location is inside the place field of the place cell. Left, comparison of the coactivity maps against chance (i.e., 0). Middle, comparison against baseline coactivity maps. Right, comparison against coactivity maps estimated using ripples from trials in which the subject is asked to remember the location of the preferred object of the object cell and in which the subject's response location is outside the place field of the place cell. Top row, analysis using all ripples; middle row, analysis using the early ripples from a given session; bottom row, analysis using the late ripples from a given session. **a2**, Number of object-cell–place-cell–ripple-channel combinations underlying the coactivity maps in **a1**. **a3**, Number of ripples underlying the coactivity maps in **a1**, pooled across object-cell–place-cell–ripple-channel combinations. **b**, Same as in **a** for the combination of re-encoding and subsequent ITI ("next ITI") phases, considering only trials in which the subject is asked to re-encode the correct location of the preferred object of the object cell and in which the object's correct location is inside the place field of the place cell. Left, comparison of the coactivity maps against chance (i.e., 0). Middle, comparison against baseline coactivity maps. Right, comparison against coactivity maps estimated using ripples from trials in which the subject is asked to re-encode the location of the preferred object of the object cell and in which the object's correct location is outside the place field of the place cell. White lines in **a** and **b** delineate significant clusters based on one-sided cluster-based permutation tests, which control for multiple comparisons and whose *P*-values are stated at the upper left. >, larger than; pref., preferred.

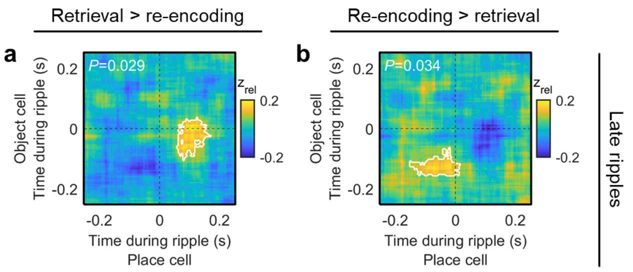

Fig. S21. Coactivity of object cells and place cells during hippocampal ripples: temporal shift between retrieval and re-encoding. a, Direct comparison of the coactivity maps from retrieval versus the coactivity maps from re-encoding. This demonstrates that the coactivations are higher for retrieval than for re-encoding at time points during and slightly after the ripple peaks (with regard to object cells and place cells, respectively). The retrieval-related coactivity maps are estimated using late retrieval-related ripples, considering only trials in which the subject is asked to remember the location of the preferred object of the object cell and in which the subject's response location is inside the place field of the place cell (Fig. 7e, left panel). The re-encoding-related coactivity maps are estimated using late re-encoding-related ripples, considering only trials in which the subject is asked to re-encode the correct location of the preferred object of the object cell and in which the object's correct location is inside the place field of the place cell (Fig. 7h, left panel). For this analysis, the coactivity *z*-scores of each cell pair were normalized (z_{rel}) so that a particular coactivity *z*-score was: $z_{rel\text{-}i} = (z_i - \min(z))$ and afterward: $z_{rel\text{-}i} = z_{rel\text{-}i} / \max(z_{rel})$, where *i* is the index of a particular coactivity *z*-score in a given coactivity map. For each cell pair, the normalized coactivity *z*-scores thus ranged between 0 and 1. This was done in order to enable the direct comparisons between retrieval- and re-encoding-related coactivity maps, as re-encoding-related coactivations were generally higher than retrieval-related coactivations (Fig. 7). **b**, Direct comparison of the coactivity maps from re-encoding versus the coactivity maps from retrieval. This demonstrates that the coactivations were higher for re-encoding than for retrieval at time points slightly earlier than the ripple peaks. Same conditions and statistical procedures as in **a**. White lines in **a** and **b** delineate significant clusters based on one-sided cluster-based permutation tests, whose *P*-values are stated in the upper left corners of the coactivity maps.

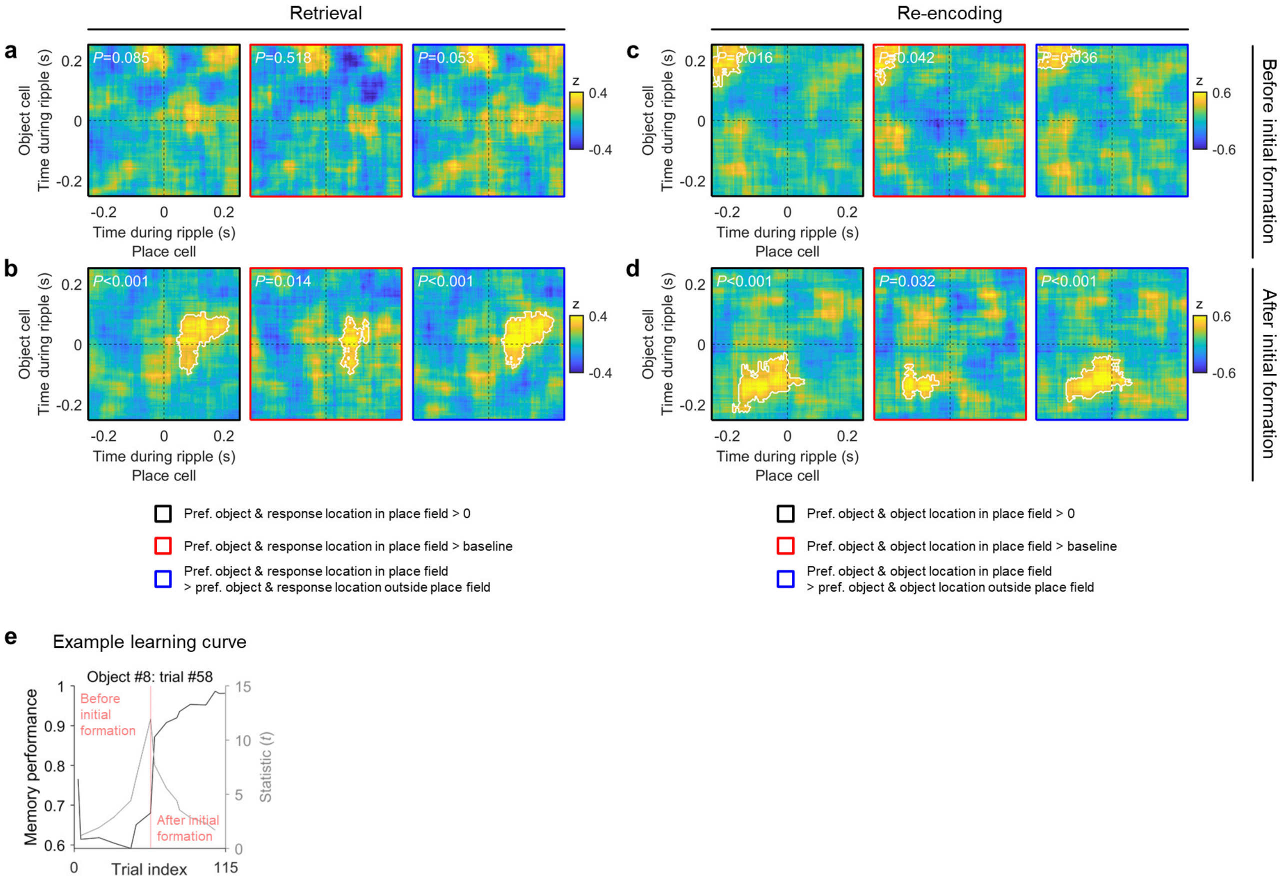

Fig. S22. Coactivity of object cells and place cells during hippocampal ripples: exploratory analysis of the effect of the initial formation of the associative memories. a–d, Same as Fig. 7d,e,g,h, but differentiating between ripples occurring "before initial formation" and ripples occurring "after initial formation" of the associative memories (instead of differentiating between early and late ripples). "Before initial formation" and "after initial formation" are defined by trial i such that the two-sample t-test between the memory performance values from trials i+1 to n and those from trials 1 to i leads to the highest t-statistic (where n is the maximum number of trials). Note though that learning still continued in trials "after initial formation" (Fig. 1d). White lines in **a–d** delineate significant clusters based on one-sided cluster-based permutation tests, whose P-values are stated in the upper left corners of the coactivity maps. >, larger than; pref., preferred. **a**, Coactivity maps estimated using ripples from the retrieval periods occurring "before initial formation," considering only trials in which the subject was asked to remember the location of the preferred object of the object cell and in which the subject's response location was inside the place field of the place cell. Left panel, comparison of the coactivity z-score maps against chance (i.e., 0). Middle panel, comparison of the coactivity z-score maps against baseline coactivity z-score maps. Right panel, comparison of the coactivity z-score maps against coactivity z-score maps estimated using ripples from trials in which the subject was asked to remember the location of the preferred object of the object cell and in which the subject's response location was outside the place field of the place cell. **b**, Same as in **a**, but only considering retrieval-related hippocampal ripples occurring "after initial formation." **c**, Coactivity maps estimated using ripples from the re-encoding periods occurring "before initial formation," considering only trials in which the subject was asked to re-encode the correct location of the preferred object of the object cell and in which the object's correct location was inside the place field of the place cell. Left panel, comparison of the coactivity z-score maps against chance (i.e., 0). Middle panel, comparison of the coactivity z-score maps against baseline coactivity z-score maps. Right panel, comparison of the coactivity z-score maps against coactivity z-score maps estimated using ripples from trials in which the subject was asked to re-encode the location of the preferred object of the object cell and in which the object's correct location was outside the place field of the place cell. **d**, Same as in **c**, but only considering re-encoding-related hippocampal ripples occurring "after initial formation." **e**, Example learning curve for object #8 during an example session. Black line, memory performance for all trials with object #8, showing a strong increase in performance around trial #58. Gray line, t-statistic from a two-sample t-test comparing the memory performance values from trials i+1 to n versus those from trials 1 to i (where n is the total number of trials). Vertical red line at trial #58, trial with the highest t-statistic and thus the strongest improvement in memory performance. Hence, for this object, trials before and during trial #58 are considered trials before initial memory formation, and trials after trial #58 are considered trials after initial memory formation.

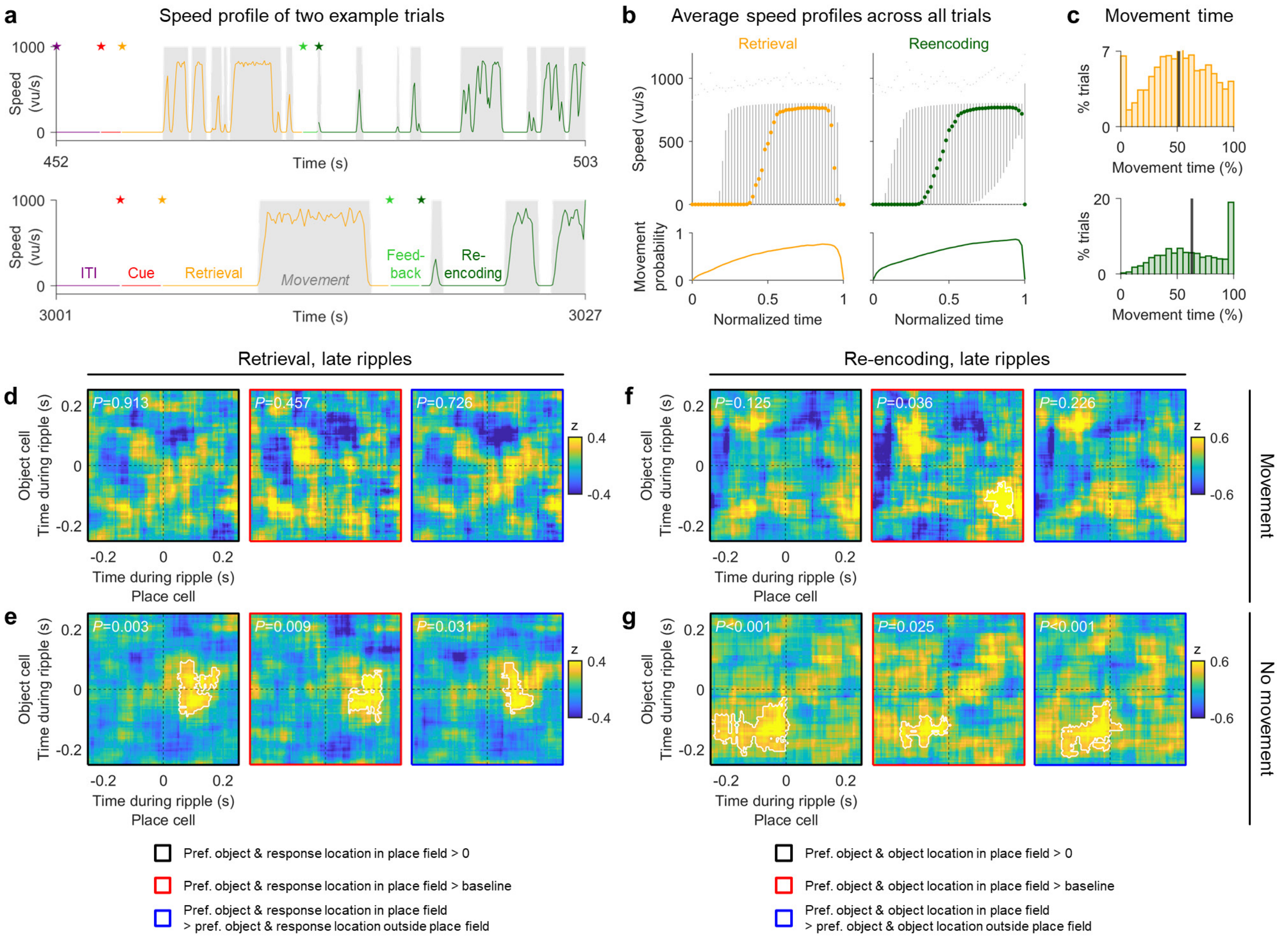

Fig. S23. Coactivity of object cells and place cells during hippocampal ripples: exploratory analysis of the effect of (non)movement. a, Speed profile of two example trials. Note that re-encoding phases typically end with movement. Gray areas, movement periods. Colored stars indicate the onsets of the different trial phases (purple, ITI; red, cue; orange, retrieval; lime green, feedback; dark green, re-encoding). **b**, Average speed profile and average movement probability across all trials (n = 4207). Colored dots in the speed profile plots indicate median per normalized time bin; gray vertical lines span the interquartile ranges; light gray dots indicate minima and maxima. Colored lines in the movement probability plots indicate mean per normalized time bin across all trials. **c**, Histograms of the movement time in each retrieval phase (top) and in each re-encoding phase (bottom), expressed in percent relative to the duration of the corresponding trial phase. **d–g**, Same as Fig. 7e,h, but only considering ripples during movement or non-movement periods. This shows that the coactivity effects were mainly driven by ripples occurring during non-movement periods. **d**, Coactivity of object cells and place cells during late hippocampal ripples from retrieval periods, only considering time periods when the subject was moving. **e**, Coactivity of object cells and place cells during late hippocampal ripples from retrieval periods, only considering time periods when the subject was not moving. **f**, Coactivity of object cells and place cells during late hippocampal ripples from re-encoding periods, only considering time periods when the subject was moving. **g**, Coactivity of object cells and place cells during late hippocampal ripples from re-encoding periods, only considering time periods when the subject was not moving. White lines in **d–g** delineate significant clusters based on one-sided cluster-based permutation tests, whose P-values are stated in the upper left corners of the coactivity maps. >, larger than; pref., preferred.

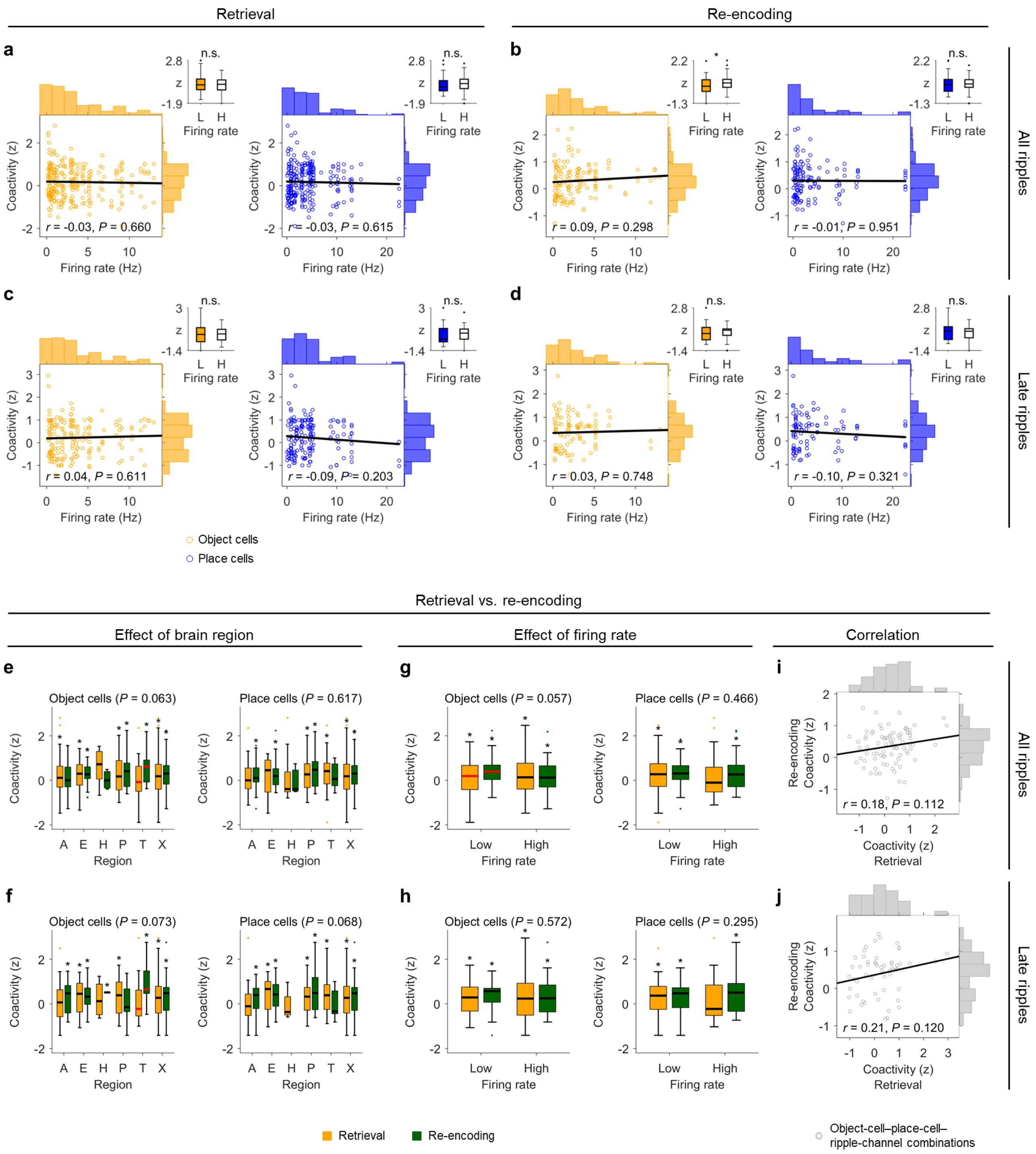

Fig. S24. Coactivity of object cells and place cells during hippocampal ripples: effects of firing rates and brain regions. **a**–**d**, No systematic associations between cellular firing rates and coactivity *z*-scores (see also Fig. S13). **a**, Scatter plots showing the firing rates of all cells and their coactivity *z*-scores during retrieval for both object cells (left; orange) and place cells (right; blue). Coactivity *z*-scores are the mean *z*-scores from the significant cluster in Fig. 7c, left. Each dot in the scatter plot represents one cell and the black line is the linear fit. Statistics (*r*- and *P*-values) are from two-sided Pearson correlations. The inset with the box plots shows coactivity *z*-scores as a function of low (L) vs. high (H) firing rate (median split). Box plots show: center line, median; box limits, upper and lower quartiles; whiskers, minimum and maximum; points, outliers. Statistics are from two-sided unpaired *t*-tests. **b**, Same as in **a** for re-encoding. Coactivity *z*-scores are the mean *z*-scores from the significant cluster in Fig. 7f, left. **c**, Same as in **a** for late ripples during retrieval. Coactivity *z*-scores are the mean *z*-scores from the significant cluster in Fig. 7e, left. **d**, Same as in **a** for late ripples during re-encoding. Coactivity *z*-scores are the mean *z*-scores from the significant cluster in Fig. 7h, left. **e**–**f**, No clear evidence for the possibility that brain regions influenced whether object cells and place cells showed higher coactivity *z*-scores during retrieval or re-encoding. *P*-values in the titles are from the interaction term between the factors brain region and trial phase using two-way ANOVAs. Asterisks, significance of two-sided one-sample *t*-tests versus 0. Box plots show: center line, median; box limits, upper and lower quartiles; whiskers, minimum and maximum; points, outliers. Pairs of box plots with red center lines, significant differences between retrieval and re-encoding based on two-sided unpaired *t*-

tests. **e**, Left: Coactivity *z*-scores as a function of brain region of the object cell and trial phase (retrieval, yellow; re-encoding, green). Two-way ANOVA: no main effect of trial phase, $F(1, 368) < 0.001$, $P = 0.986$; no main effect of brain region, $F(4, 368) = 0.260$, $P = 0.903$; no interaction, $F(4, 368) = 2.250$, $P = 0.063$. Right: Coactivity *z*-scores as a function of brain region of the place cell and trial phase. Two-way ANOVA: no main effect of trial phase, $F(1, 362) < 0.001$, $P = 0.979$; no main effect of brain region, $F(4, 362) = 1.332$, $P = 0.258$; no interaction, $F(4, 362) = 0.664$, $P = 0.617$. Retrieval-related coactivity *z*-scores are the mean *z*-scores from the significant cluster in Fig. 7c, left, and re-encoding-related coactivity *z*-scores are the mean *z*-scores from the significant cluster in Fig. 7f, left. **f**, Left: Coactivity *z*-scores as a function of brain region of the object cell and trial phase, only considering late ripples. Two-way ANOVA: no main effect of trial phase, $F(1, 251) = 2.776$, $P = 0.097$; no main effect of brain region, $F(4, 251) = 0.474$, $P = 0.754$; no interaction, $F(4, 251) = 2.166$, $P = 0.073$. Right: Coactivity *z*-scores as a function of brain region of the place cell and trial phase, only considering late ripples. Two-way ANOVA: no main effect of trial phase, $F(1, 239) = 0.108$, $P = 0.743$; main effect of brain region, $F(3, 239) = 3.002$, $P = 0.031$; no interaction, $F(3, 239) = 2.406$, $P = 0.068$. Retrieval-related coactivity *z*-scores are the mean *z*-scores from the significant cluster in Fig. 7e, left, and re-encoding-related coactivity *z*-scores are the mean *z*-scores from the significant cluster in Fig. 7h, left. **g–h**, No clear evidence for the possibility that overall firing rates influenced whether object cells and place cells showed higher coactivity *z*-scores during retrieval or re-encoding. Display and statistical conventions as in **e–f**. **g**, Left: Coactivity *z*-scores as a function of low versus high firing rate of the object cell and trial phase. Two-way ANOVA: no main effect of trial phase, $F(1, 423) = 1.821$, $P = 0.178$; no main effect of firing rate, $F(1, 423) = 0.730$, $P = 0.393$; no interaction, $F(1, 423) = 3.640$, $P = 0.057$. Right: Coactivity *z*-scores as a function of low versus high firing rate of the place cell and trial phase. Two-way ANOVA: main effect of trial phase, $F(1, 423) = 4.051$, $P = 0.045$; no main effect of firing rate, $F(1, 423) = 0.887$, $P = 0.347$; no interaction, $F(1, 423) = 0.533$, $P = 0.466$. Retrieval-related coactivity *z*-scores are the mean *z*-scores from the significant cluster in Fig. 7c, left, and re-encoding-related coactivity *z*-scores are the mean *z*-scores from the significant cluster in Fig. 7f, left. **h**, Left: Coactivity *z*-scores as a function of low versus high firing rate of the object cell and trial phase, only considering late ripples. Two-way ANOVA: no main effect of trial phase, $F(1, 288) = 2.246$, $P = 0.135$; no main effect of firing rate, $F(1, 288) = 0.436$, $P = 0.510$; no interaction, $F(1, 288) = 0.319$, $P = 0.572$. Right: Coactivity *z*-scores as a function of low versus high firing rate of the place cell and trial phase, only considering late ripples. Two-way ANOVA: main effect of trial phase, $F(1, 288) = 3.930$, $P = 0.048$; no main effect of firing rate, $F(1, 288) = 0.239$, $P = 0.625$; no interaction, $F(1, 288) = 1.101$, $P = 0.295$. Retrieval-related coactivity *z*-scores are the mean *z*-scores from the significant cluster in Fig. 7e, left, and re-encoding-related coactivity *z*-scores are the mean *z*-scores from the significant cluster in Fig. 7h, left. **i–j**, No significant correlations between the coactivity *z*-scores during retrieval as compared to those during re-encoding. Statistics (*r*- and *P*-values) are from two-sided Pearson correlations. Coactivity *z*-scores are the mean *z*-scores from the significant clusters in Fig. 7c and Fig. 7f (**i**) and the mean *z*-scores from the significant clusters in Fig. 7e and Fig. 7h (**j**). Each dot represents one object-cell–place-cell–ripple-channel combination. Only combinations of object cells, place cells, and ripple channels are plotted that exhibited coactivations during both retrieval and re-encoding. Black line, linear fit. A, amygdala; E, entorhinal cortex; H, hippocampus; P, parahippocampal cortex; T, temporal pole; X, all regions combined. n.s., not significant; $*P < 0.05$.

Supplementary References

1. Vaz, A. P., Wittig, J. H., Inati, S. K. & Zaghloul, K. A. Replay of cortical spiking sequences during human memory retrieval. *Science* **367**, 1131–1134 (2020).
2. Norman, Y. *et al.* Hippocampal sharp-wave ripples linked to visual episodic recollection in humans. *Science* **365**, eaax1030 (2019).
3. Staresina, B. P. *et al.* Hierarchical nesting of slow oscillations, spindles and ripples in the human hippocampus during sleep. *Nat. Neurosci.* **18**, 1679–1686 (2015).
4. Guth, T. A. *et al.* Interictal spikes with and without high-frequency oscillation have different single-neuron correlates. *Brain* **144**, 3078–3088 (2021).
5. Chen, Y. Y. *et al.* Stability of ripple events during task engagement in human hippocampus. *Cell Rep.* **35**, 109304 (2021).
6. Ngo, H.-V., Fell, J. & Staresina, B. Sleep spindles mediate hippocampal-neocortical coupling during long-duration ripples. *eLife* **9**, e57011 (2020).
7. Henin, S. *et al.* Spatiotemporal dynamics between interictal epileptiform discharges and ripples during associative memory processing. *Brain* **144**, 1590–1602 (2021).
8. Aarts, J. H., Binnie, C. D., Smit, A. M. & Wilkins, A. J. Selective cognitive impairment during focal and generalized epileptiform EEG activity. *Brain J. Neurol.* **107 (Pt 1)**, 293–308 (1984).
9. Vivekananda, U. *et al.* Spatial and episodic memory tasks promote temporal lobe interictal spikes. *Ann. Neurol.* **86**, 304–309 (2019).
10. Oliva, A., Fernández-Ruiz, A., Fermino de Oliveira, E. & Buzsáki, G. Origin of Gamma Frequency Power during Hippocampal Sharp-Wave Ripples. *Cell Rep.* **25**, 1693-1700.e4 (2018).
11. Sosa, M., Joo, H. R. & Frank, L. M. Dorsal and Ventral Hippocampal Sharp-Wave Ripples Activate Distinct Nucleus Accumbens Networks. *Neuron* **105**, 725-741.e8 (2020).
12. Patel, J., Schomburg, E. W., Berényi, A., Fujisawa, S. & Buzsáki, G. Local Generation and Propagation of Ripples along the Septotemporal Axis of the Hippocampus. *J. Neurosci.* **33**, 17029–17041 (2013).
13. Dickey, C. W. *et al.* Cortical Ripples during NREM Sleep and Waking in Humans. *J. Neurosci.* **42**, 7931–7946 (2022).
14. Axmacher, N., Elger, C. E. & Fell, J. Ripples in the medial temporal lobe are relevant for human memory consolidation. *Brain* **131**, 1806–1817 (2008).
15. Vaz, A. P., Inati, S. K., Brunel, N. & Zaghloul, K. A. Coupled ripple oscillations between the medial temporal lobe and neocortex retrieve human memory. *Science* **363**, 975–978 (2019).
16. Norman, Y., Raccah, O., Liu, S., Parvizi, J. & Malach, R. Hippocampal ripples and their coordinated dialogue with the default mode network during recent and remote recollection. *Neuron* **109**, 2767-2780.e5 (2021).
17. Sakon, J. J. & Kahana, M. J. Hippocampal ripples signal contextually mediated episodic recall. *Proc. Natl. Acad. Sci.* **119**, e2201657119 (2022).
18. Liu, A. A. *et al.* A consensus statement on detection of hippocampal sharp wave ripples and differentiation from other fast oscillations. *Nat. Commun.* **13**, 6000 (2022).
19. Joo, H. R. & Frank, L. M. The hippocampal sharp wave–ripple in memory retrieval for immediate use and consolidation. *Nat. Rev. Neurosci.* **19**, 744–757 (2018).

20. Buzsáki, G. & Silva, F. L. da. High frequency oscillations in the intact brain. *Prog. Neurobiol.* **98**, 241–249 (2012).

21. Ray, S. & Maunsell, J. H. R. Different Origins of Gamma Rhythm and High-Gamma Activity in Macaque Visual Cortex. *PLOS Biol.* **9**, e1000610 (2011).

22. Miller, K. J. Broadband Spectral Change: Evidence for a Macroscale Correlate of Population Firing Rate? *J. Neurosci.* **30**, 6477–6479 (2010).

23. Osipova, D. *et al.* Theta and Gamma Oscillations Predict Encoding and Retrieval of Declarative Memory. *J. Neurosci.* **26**, 7523–7531 (2006).

24. Sederberg, P. B. *et al.* Gamma Oscillations Distinguish True From False Memories. *Psychol. Sci.* **18**, 927–932 (2007).

25. Yamamoto, J., Suh, J., Takeuchi, D. & Tonegawa, S. Successful Execution of Working Memory Linked to Synchronized High-Frequency Gamma Oscillations. *Cell* **157**, 845–857 (2014).

26. Staresina, B. P. *et al.* Hippocampal pattern completion is linked to gamma power increases and alpha power decreases during recollection. *eLife* **5**, e17397 (2016).

27. Treder, M. S. *et al.* The hippocampus as the switchboard between perception and memory. *Proc. Natl. Acad. Sci.* **118**, e2114171118 (2021).

28. Lachner-Piza, D. *et al.* Effects of Spatial Memory Processing on Hippocampal Ripples. *Front. Neurol.* **12**, 237 (2021).

29. Karimi Abadchi, J. *et al.* Spatiotemporal patterns of neocortical activity around hippocampal sharp-wave ripples. *eLife* **9**, e51972 (2020).

30. Pennartz, C. M. A. *et al.* The Ventral Striatum in Off-Line Processing: Ensemble Reactivation during Sleep and Modulation by Hippocampal Ripples. *J. Neurosci.* **24**, 6446–6456 (2004).

31. Gomperts, S. N., Kloosterman, F. & Wilson, M. A. VTA neurons coordinate with the hippocampal reactivation of spatial experience. *eLife* **4**, e05360 (2015).

32. Nitzan, N., Swanson, R., Schmitz, D. & Buzsáki, G. Brain-wide interactions during hippocampal sharp wave ripples. *Proc. Natl. Acad. Sci.* **119**, e2200931119 (2022).

33. Dickey, C. W. *et al.* Widespread ripples synchronize human cortical activity during sleep, waking, and memory recall. *Proc. Natl. Acad. Sci.* **119**, e2107797119 (2022).

34. Olsen, R., Moses, S., Riggs, L. & Ryan, J. The hippocampus supports multiple cognitive processes through relational binding and comparison. *Front. Hum. Neurosci.* **6**, (2012).

35. Yonelinas, A. P., Ranganath, C., Ekstrom, A. D. & Wiltgen, B. J. A contextual binding theory of episodic memory: systems consolidation reconsidered. *Nat. Rev. Neurosci.* **20**, 364–375 (2019).

36. Wilson, D. I. G. *et al.* Lateral entorhinal cortex is critical for novel object-context recognition. *Hippocampus* **23**, 352–366 (2013).

37. Igarashi, K. M., Lu, L., Colgin, L. L., Moser, M.-B. & Moser, E. I. Coordination of entorhinal–hippocampal ensemble activity during associative learning. *Nature* **510**, 143–147 (2014).

38. Staresina, B. P. *et al.* Recollection in the human hippocampal-entorhinal cell circuitry. *Nat. Commun.* **10**, 1503 (2019).

39. Symanski, C. A., Bladon, J. H., Kullberg, E. T., Miller, P. & Jadhav, S. P. Rhythmic coordination and ensemble dynamics in the hippocampal-prefrontal network during odor-place associative memory and decision making. *eLife* **11**, e79545 (2022).

40. Lee, J. Y. *et al.* Dopamine facilitates associative memory encoding in the entorhinal cortex. *Nature* **598**, 321–326 (2021).

41. Vandrey, B. *et al.* Fan Cells in Layer 2 of the Lateral Entorhinal Cortex Are Critical for Episodic-like Memory. *Curr. Biol.* **30**, 169-175.e5 (2020).

42. Viskontas, I. V., Ekstrom, A. D., Wilson, C. L. & Fried, I. Characterizing interneuron and pyramidal cells in the human medial temporal lobe in vivo using extracellular recordings. *Hippocampus* **17**, 49–57 (2007).

43. Ison, M. J. *et al.* Selectivity of pyramidal cells and interneurons in the human medial temporal lobe. *J. Neurophysiol.* **106**, 1713–1721 (2011).
