# Supplementary material for: Ripple-locked coactivity of stimulus-specific neurons and human associative memory
Source: Nat Neurosci. 2024 Feb 16;27(3):587–99. doi: 10.1038/s41593-023-01550-x (PMC10917673; doi:10.1038/s41593-023-01550-x)
Supplement: Supplementary file 2 — Reporting Summary [file 41593_2023_1550_MOESM2_ESM.pdf]

nature portfolio

Corresponding author(s): Lukas Kunz

Last updated by author(s): Dec 6, 2023

Reporting Summary

Nature Portfolio wishes to improve the reproducibility of the work that we publish. This form provides structure for consistency and transparency in reporting. For further information on Nature Portfolio policies, see our Editorial Policies and the Editorial Policy Checklist.

Statistics

For all statistical analyses, confirm that the following items are present in the figure legend, table legend, main text, or Methods section.

n/a	Confirmed	
☐	☒	The exact sample size (n) for each experimental group/condition, given as a discrete number and unit of measurement
☐	☒	A statement on whether measurements were taken from distinct samples or whether the same sample was measured repeatedly
☐	☒	The statistical test(s) used AND whether they are one- or two-sided<br>*Only common tests should be described solely by name; describe more complex techniques in the Methods section.*
☐	☒	A description of all covariates tested
☐	☒	A description of any assumptions or corrections, such as tests of normality and adjustment for multiple comparisons
☐	☒	A full description of the statistical parameters including central tendency (e.g. means) or other basic estimates (e.g. regression coefficient) AND variation (e.g. standard deviation) or associated estimates of uncertainty (e.g. confidence intervals)
☐	☒	For null hypothesis testing, the test statistic (e.g. F, t, r) with confidence intervals, effect sizes, degrees of freedom and P value noted<br>*Give P values as exact values whenever suitable.*
☒	☐	For Bayesian analysis, information on the choice of priors and Markov chain Monte Carlo settings
☐	☒	For hierarchical and complex designs, identification of the appropriate level for tests and full reporting of outcomes
☐	☒	Estimates of effect sizes (e.g. Cohen's d, Pearson's r), indicating how they were calculated

Our web collection on statistics for biologists contains articles on many of the points above.

Software and code

Policy information about availability of computer code

Data collection	The memory task was programmed using Unreal Engine 2 (Epic Games, Cary, NC, USA). Neurophysiological data were collected using a NeuroPort System (Blackrock Microsystems, Salt Lake City, UT, USA) and hybrid depth electrodes (Ad-Tech, Racine, WI, USA). See the Methods section for a detailed description.
Data analysis	Data analyses were carried out in MATLAB 2020b and 2021b (The MathWorks, Inc., Natick, MA, USA), using MATLAB toolboxes and custom MATLAB code. Custom MATLAB code can be downloaded from https://github.com/NeuroLuke/KunzNatureNeuroscience2024. Circular statistics were performed using the CircStat toolbox, version 1.21.0.0 (Berens, 2009). Local field potentials were analyzed using FieldTrip (version 20210614). Spike sorting was done using Wave_Clus 3 (Chaure et al., 2018). MNI coordinates of depth electrodes were determined using PyLocator (v1.0). See the Methods section for a detailed description.

For manuscripts utilizing custom algorithms or software that are central to the research but not yet described in published literature, software must be made available to editors and reviewers. We strongly encourage code deposition in a community repository (e.g. GitHub). See the Nature Portfolio guidelines for submitting code & software for further information.

Data

Policy information about availability of data

All manuscripts must include a data availability statement. This statement should provide the following information, where applicable:

- Accession codes, unique identifiers, or web links for publicly available datasets
- A description of any restrictions on data availability
- For clinical datasets or third party data, please ensure that the statement adheres to our policy

Data to recreate the figures can be downloaded from https://github.com/NeuroLuke/KunzNatureNeuroscience2024. Raw data are not publicly available because they could compromise research participant privacy, but are available upon request from the corresponding author, Lukas Kunz. Any additional information required to reanalyze the data reported in this paper is available from the corresponding author upon request. Requests will typically be answered within one week. Researchers requesting the data will have to sign an agreement that they will not try to de-identify the data and that they will use the data for scientific purposes only.

Human research participants

Policy information about studies involving human research participants and Sex and Gender in Research.

Reporting on sex and gender	The final sample of participants comprised 30 subjects (16 female and 14 male). The findings of this study, which are about the neural mechanisms underlying associative memory in humans, presumably apply equally to all individuals and sex/gender was therefore not considered in the study design. Sex/gender was determined based on the self-reported data of the subjects.
Population characteristics	We tested N = 35 human subjects, who were epilepsy patients undergoing treatment for pharmacologically intractable epilepsy at the Freiburg Epilepsy Center, Freiburg im Breisgau, Germany. Of those, 5 patients had to be excluded because of technical issues (n = 1); no hippocampal electrode contacts (n = 2); hippocampal channels that were close to the resection border of a previous surgery (n = 1); and a very low number of ripples (n = 1). This resulted in a final sample of n = 30 subjects (16 female; age range, 19–61 years; mean age ± SEM, 36 ± 2 years), contributing a total of n = 41 experimental sessions with intracranial EEG recordings including the left and/or right hippocampus (n = 62 hippocampal bipolar channels). For 20 of these 30 subjects, additional single-neuron recordings from various MTL regions were available (n = 27 sessions; n = 43 hippocampal bipolar channels). Further subject information is presented in Table S1.
Recruitment	Subjects undergoing invasive electrophysiological recordings for clinical purposes were recruited and consented to participate in this study. Subjects who were capable of and willing to perform the task were recruited. There may be effects of self-selection bias or other biases, but they are unlikely to affect the study results, as this study is about basic neural mechanisms underlying associative memory in humans.
Ethics oversight	Experimental procedures were approved by the Ethics Committee of the University of Freiburg, Freiburg im Breisgau, Germany, and all participants provided written informed consent.

Note that full information on the approval of the study protocol must also be provided in the manuscript.

Field-specific reporting

Please select the one below that is the best fit for your research. If you are not sure, read the appropriate sections before making your selection.

☒ Life sciences ☐ Behavioural & social sciences ☐ Ecological, evolutionary & environmental sciences

For a reference copy of the document with all sections, see nature.com/documents/nr-reporting-summary-flat.pdf

Life sciences study design

All studies must disclose on these points even when the disclosure is negative.

Sample size	Our single-neuron analyses were based on 1063 neurons recorded across 27 sessions from 20 subjects. Analyses of hippocampal ripples were based on 62 hippocampal channels across 41 sessions from 30 subjects. No statistical method was used to predetermine sample size. Instead, sample sizes were determined based on typical sample sizes in the field that are deemed sufficient for statistical analyses (e.g., Norman et al., Science, 2019; Qasim et al., Nature Neuroscience, 2019; Kutter et al., Neuron, 2018; Rutishauser et al., Nature, 2010). See the Methods for details.
Data exclusions	No data were excluded. See also "Population characteristics" above.
Replication	All analyses were performed on the entire available data and significant effects replicated across the underlying samples. No separate replication study was performed.
Randomization	All subjects were in the same experimental group and no randomization of the subjects was required.

Blinding	Subjects were not aware of the goals of the study. There was no subjective measurement or decision that the investigator needed to make during the experiment. All data were analyzed off-line. Data collection and analyses were not performed blind to the conditions of the experiments as conditional information was required for further analyses.

Reporting for specific materials, systems and methods

We require information from authors about some types of materials, experimental systems and methods used in many studies. Here, indicate whether each material, system or method listed is relevant to your study. If you are not sure if a list item applies to your research, read the appropriate section before selecting a response.

Materials & experimental systems

n/a	Involved in the study
☒	☐ Antibodies
☒	☐ Eukaryotic cell lines
☒	☐ Palaeontology and archaeology
☒	☐ Animals and other organisms
☒	☐ Clinical data
☒	☐ Dual use research of concern

Methods

n/a	Involved in the study
☒	☐ ChIP-seq
☒	☐ Flow cytometry
☐	☒ MRI-based neuroimaging

Magnetic resonance imaging

Experimental design

Design type	MRI were acquired purely for clinical purposes to indicate electrode placement, and were not a part of the experiment.
Design specifications	MRI were acquired purely for clinical purposes to indicate electrode placement, and were not a part of the experiment.
Behavioral performance measures	MRI were acquired purely for clinical purposes to indicate electrode placement, and were not a part of the experiment.

Acquisition

Imaging type(s)	Structural MRI.
Field strength	3T before electrode implantation; 1.5T after electrode implantation.
Sequence & imaging parameters	Pre-implant 3D T1-weighted MPRAGE (Siemens Prisma, Germany): TR 2,000 ms; TE 2.26 ms; flip angle 12; 1 mm isotropic resolution; 256 x 256 x 160 matrix.<br>Post-implant 3D T1-weighted MPRAGE (Siemens Avanto, Germany): TR 1,300 ms; TE 2.33 ms; flip angle 15; 0.5 x 0.5 x 1 mm resolution; 512 x 512 x 176 matrix.
Area of acquisition	Whole brain.

Diffusion MRI ☐ Used ☒ Not used

Preprocessing

Preprocessing software	SPM (https://www.fil.ion.ucl.ac.uk/spm/).
Normalization	Normalization was performed using SPM.
Normalization template	Normalization was performed using the SPM template.
Noise and artifact removal	No noise or artifact removal was used.
Volume censoring	No volume censoring was used.

Statistical modeling & inference

Model type and settings	No statistical modeling was used as MRI scans were only acquired for clinical purposes to indicate electrode placement.
Effect(s) tested	No effects were tested as MRI scans were only acquired for clinical purposes to indicate electrode placement.

Specify type of analysis: ☒ Whole brain ☐ ROI-based ☐ Both

Statistic type for inference
(See Eklund et al. 2016)

No statistical analyses were performed.

Correction

No statistical analyses were performed and no correction was applied.

Models & analysis

n/a	Involved in the study
☒	☐ Functional and/or effective connectivity
☒	☐ Graph analysis
☒	☐ Multivariate modeling or predictive analysis
